# Single-allele nanoscale mapping of regulatory variants

**DOI:** 10.1038/s41588-026-02776-8

**Published:** 2026-10-02

**Authors:** Joseph C. Hamley, Weijiao Zhang, Daniel Willmott, Lucia Y. Chen, Vassilena Sharlandjieva, Hangpeng Li, James L. T. Dalgleish, Nicholas Denny, Gaurav Agarwal, Lance Hentges, Bora Ozcan, Roman M. Doll, Ye Wei, Simone G. Riva, Samvida S. Venkatesh, Marta Arachi, Devika Agarwal, Evgeny E. Akkuratov, Matthew Baxter, Tatjana Sauka-Spengler, Calliope A. Dendrou, Thomas A. Milne, Jim R. Hughes, James O. J. Davies

**Affiliations:** 1 MRC Molecular Haematology Unit, MRC Weatherall Institute of Molecular Medicine, Radcliffe Department of Medicine, University of Oxford, Oxford, UK; 2 National Institute of Health Research Blood and Transplant Research Unit in Precision Cellular Therapeutics, Oxford, UK; 3 MRC WIMM Centre for Computational Biology, MRC Weatherall Institute of Molecular Medicine, University of Oxford, Oxford, UK; 4 Division of Hematology/Oncology, Boston Children’s Hospital, Harvard Medical School, Boston, MA USA; 5 Kennedy Institute of Rheumatology, University of Oxford, Oxford, UK; 6 Oxford National Institute of Health Research Biomedical Research Centre, University of Oxford, Oxford, UK

**Keywords:** Functional genomics, Genomics, Gene regulation

## Abstract

Millions of genetic variants are linked to human disease but identifying underlying mechanisms is challenging because most variants are noncausal and lie within the noncoding genome. We developed a Micro Capture-C variant-to-function platform (MCCv) based on analysis of single-allele chromatin structure. This can identify changes in nanoscale chromatin architecture and link variants in *cis*-regulatory elements to target genes. Furthermore, MCCv can phase other heterozygous variants within a locus to link regulatory variants to allelically imbalanced gene expression and directly read out variant effects on chromatin interactions after genome editing. With this approach, we investigated 405 *cis*-regulatory elements linked to immune-mediated inflammatory disease in CD4^+^ T cells. We uncovered a previously undescribed gain-of-function mechanism, which increases risk of autoimmunity through creation of a neo-CTCF motif that blocks super-enhancer contacts with the *SESN3* promoter. We showed that *SESN3* regulates mammalian target of rapamycin by sensing tryptophan and demonstrated its role in autoimmunity using mouse models.

## Abstract (web summary)

This study uses high-resolution, allele-specific chromatin architecture to identify causal gene-regulatory mechanisms in inflammatory diseases and uncovers a mechanism by which a neo-CTCF site promotes autoimmunity.

## Main

Over 10 million people have been genotyped and the vast majority of variants identified are in noncoding regions, irrespective of the trait or disease being studied^1–4^. Although we have been able to interpret protein-coding sequences since seminal studies in the 1960s, interpretation of the vast noncoding genome, which makes up >95% of the total sequence, remains extremely challenging^5^. The noncoding genome contains *cis*-regulatory elements, which control when and where genes are expressed, and >90% of the variants linked to common disease^6^. In addition, noncoding variants have been shown to cause inherited disease^7^ and drive malignancy^8^. Although the data are routinely generated by whole-genome sequencing, the noncoding sequences are largely unused in clinical genetics due to our limited ability to interpret them^9^. This therefore remains a huge untapped resource for identification of new pathways and therapeutic targets for treating human disease^10^.

Most functional noncoding variants dysregulate the intricate process of gene expression, through alteration of *cis*-regulatory sequences^11,12^. One of the key difficulties is that the noncoding genome is not read linearly: *cis*-regulatory elements are distributed unpredictably and can lie over a million basepairs away from the genes that they control, often within the introns of other genes^13^. Distal *cis*-regulatory elements, termed enhancers, which are bound by clusters of transcription factors, are believed to come into close physical proximity with the regulatory sequences at the promoter, which lie near to the transcription start site. The specificity of these interactions is modulated by CTCF-bound elements, which can both limit and promote the physical connection between enhancers and promoters through cohesin-mediated loop extrusion^14,15^. Thus, chromatin structure is a key determinant of gene expression^16^. A further challenge is that many genetic associations are in linkage with tens to hundreds of other variants, meaning that <5% of disease-associated variants are functionally active^17^. As a result, although the number of disease-associated variants has increased exponentially^2^, the number of validated causal genes and mechanistic insights has not kept pace^10^.

At present, chromosome conformation capture (3C) techniques are the best available methods to map sequence-specific connectivity between *cis*-regulatory elements and the genes to which they are linked^18^. Although 3C methods have been used to map the three-dimensional (3D) structure of the genome to ever-increasing resolutions and throughput^19–22^, a lack of sensitivity has precluded their use to assay the functionality of variant alleles directly. Importantly, most 3C methods are restriction-enzyme based and only generate data from the sequence-specific cut-sites. As a result, resolutions <500 bp cannot be achieved, precluding single-allele-resolution data being generated for most variants^19^.

**Fig. 1:**
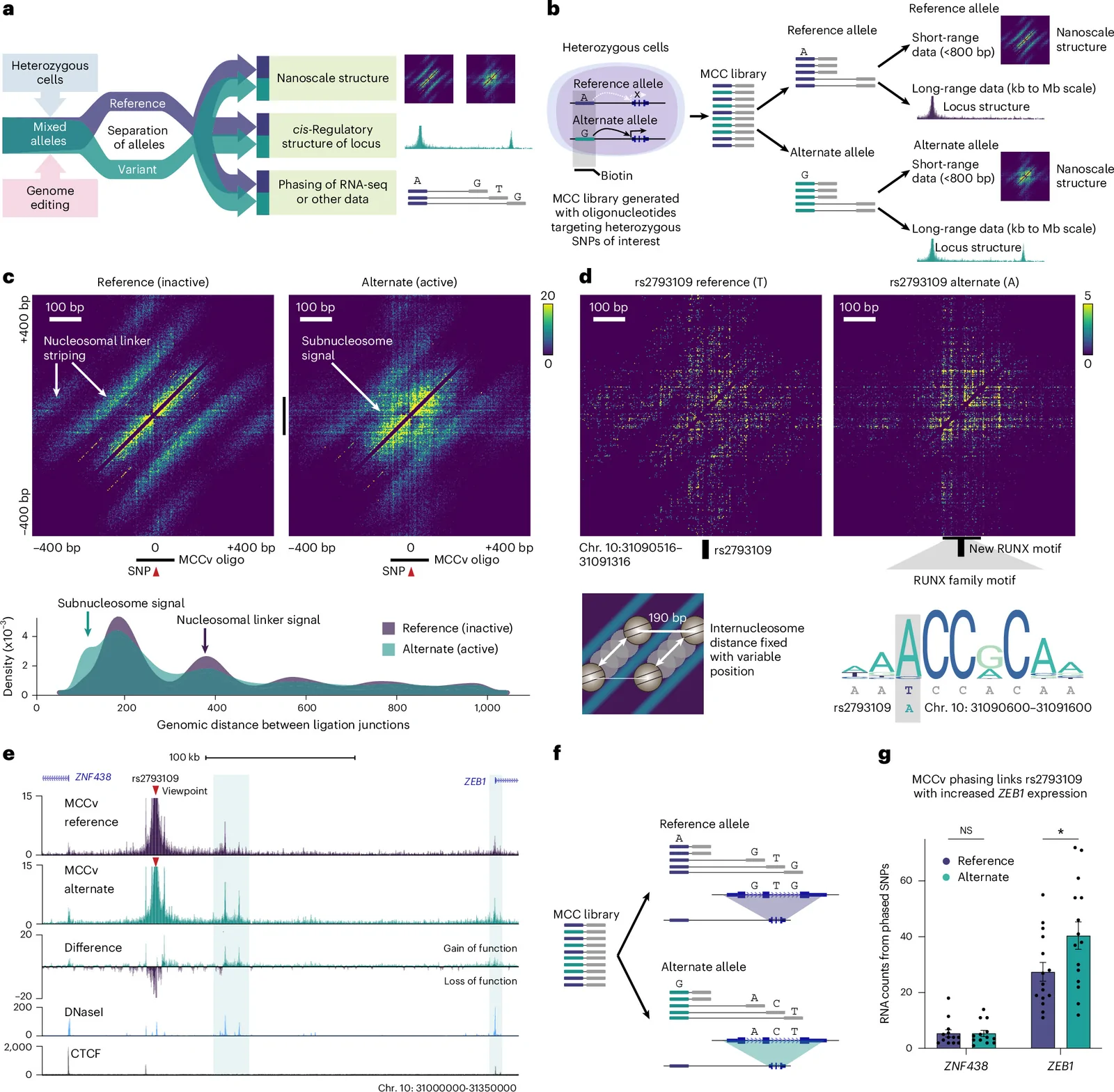
Development of the MCCv platform. **a**, Schematic of the MCCv platform including: nanoscale chromatin structure, the regulatory landscape of the locus and phasing of other alleles in the locus. **b**, Schematic of the MCCv method. Oligonucleotide probes are designed over the variant of interest in heterozygous cells. Ultra-short-range ligation junctions (<800 bp) define the nanoscale topology at the variant. Long-range junctions (1 kb to 1 Mb) define the locus structure. **c**, Metaplots of MCCv nanoscale chromatin architecture heatmaps for inactive (reference) and active (alternate) alleles from 41 gain-of-function enhancer variants (Jurkat cells, six replicates, 1-bp bin size). The matrix represents the contact frequency between short-range ligation junctions (<800 bp) to visualize nanoscale chromatin architecture from single alleles. Below is a kernel density estimate (KDE) plot of genomic distances between two basepairs that make up the ultra-short-range ligation junctions for both reference and alternate alleles. **d**, Individual 2D nanoscale chromatin architecture heatmap for reference (left) and alternate (right) alleles of rs2793109 (1-bp bin size, *n* = 6, merged *β* = 0.75). The schematic is for nucleosome sliding with a fixed internucleosomal distance in the inactive alleles. At the nucleosome-depleted region, contacts between potential transcription factors in the alternate allele may lead to regions of high signal adjacent to transcription factor-binding sites. The underlying *RUNX* family motif is shown with the effect of the rs2793109 alternate allele highlighted. **e**, MCCv from rs2793109. A difference plot (reference minus alternate), DNase I sequencing and CTCF ChIP–seq are shown underneath. **f**, Using MCCv to phase other heterozygous variants within a locus. **g**, TT-seq skew from MCCv-phased variants in introns of *ZNF438* (*n* = 13) and *ZEB1* (*n* = 15) (mean ± s.e.m., Wilcoxon’s signed-rank test, paired, two-sided, two-stage step-up procedure for multiple-test correction: *ZNF438 q* = 0.435; *ZEB1 q* = 0.0002). NS, not significant; oligo, oligonucleotide. Source data

To directly identify functional variants and their associated genes, we developed a Micro Capture-C (MCC)-based variant-to-function platform (MCCv) (Fig. 1a). This generates the highest-resolution 3C data to date, allowing functional variants to be directly identified through fine-scale mapping of chromatin structure at the level of individual transcription factor-binding sites while simultaneously identifying target genes using long-range interactions^23,24^. To minimize experimental noise and sensitively resolve functional changes, MCCv delineates single-allele interactions in heterozygous or genome-edited cells (Fig. 1b). In addition the platform enables integration of MCC data with other high-throughput functional genomics assays including RNA sequencing (RNA-seq) through locus-specific phasing.

We applied the MCCv platform to systematically interrogate autoimmune disease loci identified by genome-wide association studies (GWASs), focusing on noncoding variation within *cis*-regulatory elements in human CD4^+^ T cells. This analysis uncovered a gain-of-function mechanism at the *SESN3* locus, which increases the risk of autoimmunity through creation of a neo-CTCF motif that blocks super-enhancer–promoter contacts. The MCCv platform allows causal variants, genes and pathways to be identified from noncoding human genetics.

## Results

### Direct identification of functional variants using basepair-resolution, single-allele chromatin structure

**Extended Data Fig. 1:**
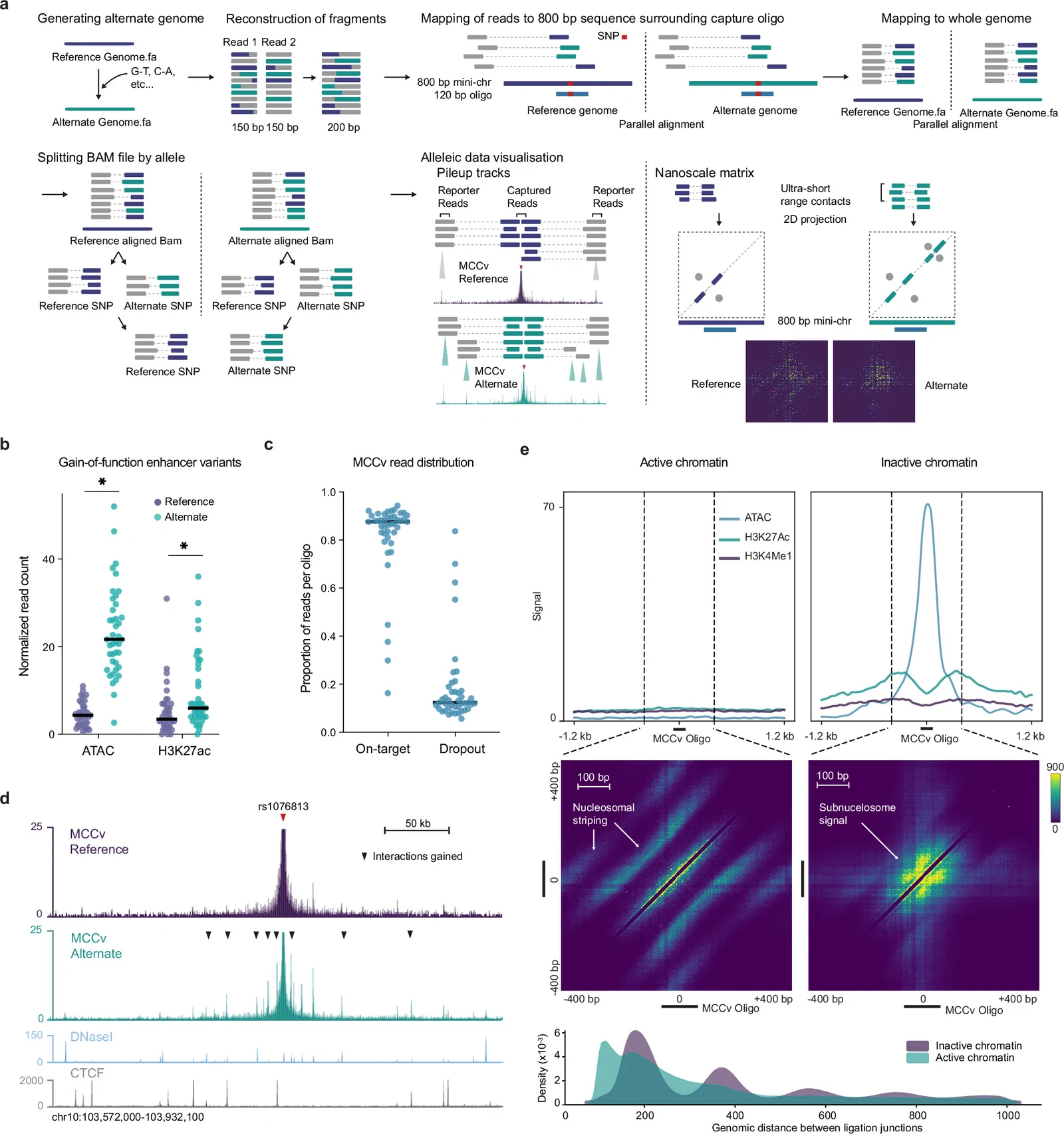
Optimisation and validation of MCCv at gain-of-function enhancers. **a**, Schematic overview of the MCCv bioinformatics suite. **b**, Skew data for ATAC and H3K27ac ChIP sequencing at the selected 42 enhancer elements demonstrating potential gain-of-function activity at the enhancer (n = 42 variants, read counts averaged across 3 independent donors, per variant reference vs. alternate comparison – Wilcoxon sign-rank test, two-sided, two-stage step-up procedure for multiple test correction, q < 0.000001 (ATAC) and q = 0.000029 (H3K27ac)). **c**, Quantification of allelic dropout post MCCv single-allele analysis. Each SNP is shown as an individual datapoint (n = 41 variants with read counts averaged across 6 independent MCC libraries, on-target median = 87.6%, dropout median = 12.3%). **d**, MCCv from the rs1076813 SNP. DNase-seq and CTCF ChIP-seq tracks for this locus are shown underneath. The interactions gained in the alternate allele are highlighted with black triangles. **e**, Control capture of independent sites corresponding to inactive (n = 81) and active chromatin (n = 92). Chromatin signature defined by ATAC-seq and H3K27ac/H3K4me1 ChIP-seq metaplots (2.4 kb region centred around the oligo). Nanoscale metaplots are 1 bp bin size and mirrored on the diagonal. Kernel estimate density (KDE) plot shown underneath for the genomic distance between the two sites which form an ultra-short unique ligation junction, at both inactive chromatin (purple) and active chromatin (green) sites. Source data

We present a method capable of analyzing short-range contact data (<800 bp) from individual alleles to identify variants that alter local chromatin topology by mapping the nanoscale chromatin architecture at basepair resolution. We discovered that it is possible to use the short-range contact data that are usually discarded in conventional 3C analysis to produce Hi-C-like matrices of chromatin structure at basepair resolution. These data allow nucleosome positioning to be delineated and enable contacts between transcription factor motifs to be resolved within nucleosome-depleted regions at individual *cis*-regulatory elements. The assay was optimized to generate deep datasets from primary cells and we developed a specific analysis pipeline to generate data in an allele-specific manner while mitigating mapping bias (Fig. 1a,b and Extended Data Fig. 1a).

We initially used MCCv to analyze 42 heterozygous gain-of-function variants in putative enhancers in Jurkat cells identified by skew-in assay of transposase-accessible chromatin sequencing (ATAC–seq) and H3K27ac chromatin immunoprecipitation sequencing (ChIP–seq)^25,26^. The selected variants exhibited greater accessibility on the alternate allele and were used to benchmark the MCCv platform at likely functional sites where mapping bias is expected to underestimate effect sizes (Extended Data Fig. 1b). In contrast to conventional restriction-enzyme-based 3C methods, MCCv allows the viewpoints to be precisely directed to the variant of interest, resulting in 86.7% of reads being used in allelic analysis, which is critical to maintaining resolution (Extended Data Fig. 1c,d).

Using the short-range nanoscale interactions from both the reference and the alternate alleles, we were able to identify changes in structure between active and inactive chromatin states. Meta-analysis of all sites showed that the inactive reference alleles display nucleosomal patterning. In contrast, active alternate alleles showed specific nanoscale structures, potentially due to contacts between transcription factors within the nucleosome-depleted region (Fig. 1c). Similar MCCv nanoscale patterns were able to distinguish distinct inactive and active enhancers (Extended Data Fig. 1e).

**Extended Data Fig. 2:**
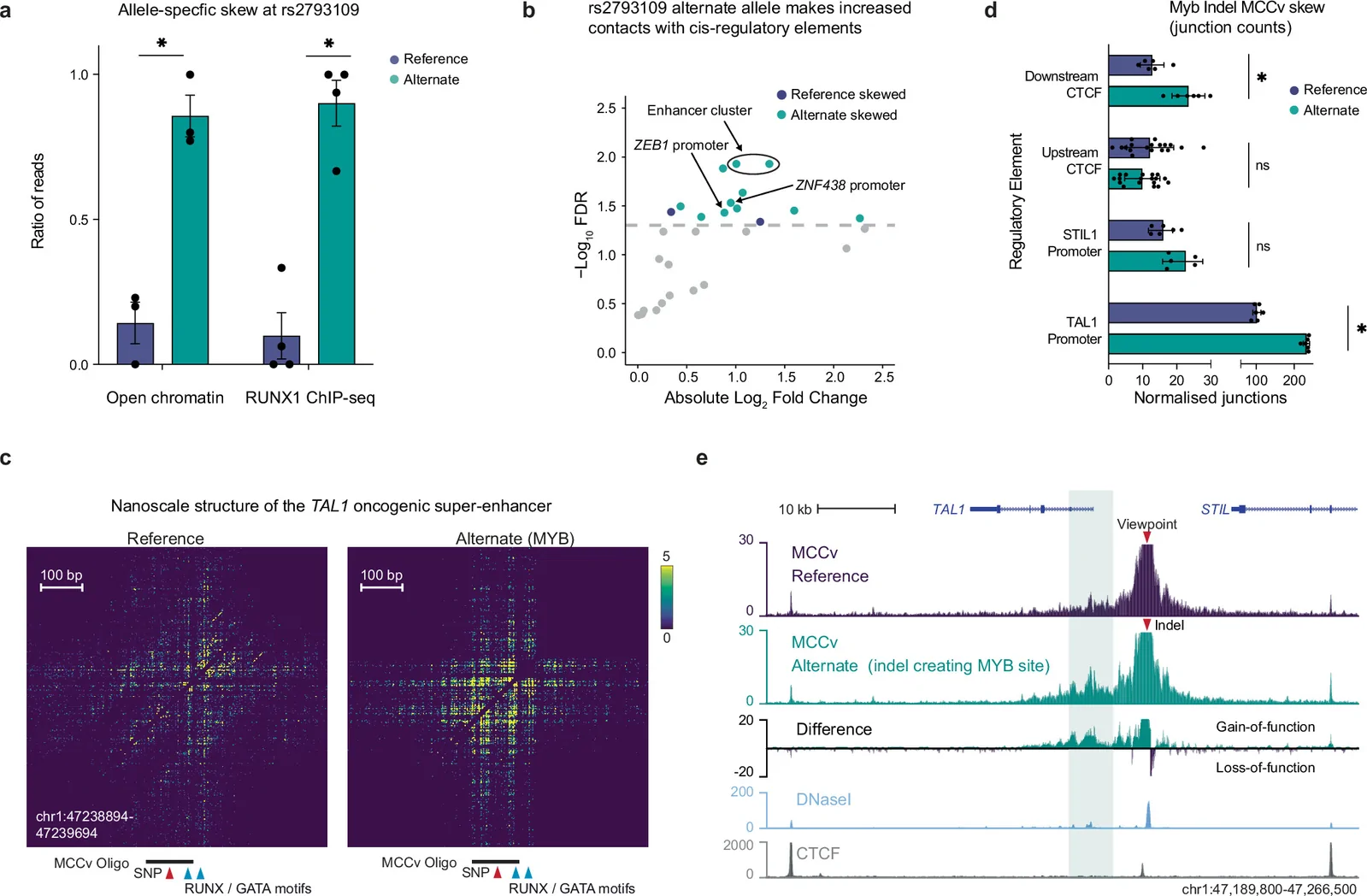
MCCv at an oncogenic super-enhancer. **a**, Quantification of ATAC and RUNX1 ChIP-seq skew at rs2793109 (mean ± s.e.m., paired Student’s t-test, two-sided, two-stage step-up procedure for multiple test correction; ATAC – n = 3 biological replicates, q = 0.039; RUNX1 ChIP-seq – n = 4 biological replicates, q = 0.030). **b**, Quantification of MCCv junction count differences between reference and alternate alleles at MCCv peaks for the rs2793109 variant. Statistically significant variants highlighted in purple (reference) or green (alternate) depending on direction of skew (n = 6, paired Student’s t-test, two-sided, local FDR estimation (FDRtools)). **c**, Quantification of differential MCCv contacts between reference and alternate alleles of oncogenic super-enhancer at different regulatory elements within the locus (Downstream CTCF q = 0.008, Upstream CTCF q = 0.028, *STIL1* Promoter q = 0.042, *TAL1* Promoter = 0.000002) (n = 6 independent MCC libraries, Wilcoxon sign-rank test, two-sided, two-stage step-up procedure for multiple test correction, mean ± s.d.). **d**, MCCv from the *TAL1* oncogenic super-enhancer. Jurkat DNaseI-seq and CTCF ChIP-seq for the locus are shown underneath. **e**, Nanoscale heatmaps for the oncogenic *TAL1* super-enhancer variant. Alternate allele map, which includes the MYB-motif indel, shows increased nanoscale contacts. Heatmap data is mirrored on the diagonal. A variety of other transcription factor motifs present near nanoscale contacts present on the reference map are indicated with blue triangles (1 bp bin size, n = 6 independent MCC libraries merged, merged β = 1.02). Source data

Analysis of rs2793109, which is associated with rheumatoid arthritis, showed that the alternate disease-associated allele creates a complex fine-scale pattern of short-range contacts, which is likely caused by the creation of a RUNX1 motif (Fig. 1d and Extended Data Fig. 2a). MCCv data simultaneously delineated allele-specific, genome-wide contact data from the variant of interest. This identified allele-specific changes in long-range interactions with enhancers and two gene promoters within the locus (*ZNF438* and *ZEB1*) (Fig. 1e and Extended Data Fig. 2b).

The MCCv platform can also be used to phase the variant at the viewpoint with other heterozygous single-nucleotide polymorphisms (SNPs) across the locus (Fig. 1f). Using this approach, we phased 13 and 15 heterozygous intronic SNPs with high confidence in the gene bodies of *ZNF438* and *ZEB1*, respectively. This allowed us to analyze transient transcriptome sequencing (TT-seq), which has high intronic coverage, in an allele-specific manner. This demonstrated that the alternate risk allele is associated with an increased expression of *ZEB1* but not *ZNF438* (Fig. 1g).

To demonstrate that the MCCv platform can also interrogate other types of pathogenic noncoding mutations, we included a well-characterized oncogenic insertion, which creates a MYB-binding site upstream of the *TAL1* oncogene^8^. MCCv showed that addition of MYB binding causes marked changes in the nanoscale allele-specific maps (Extended Data Fig. 2c). In addition, we showed a specific increase in enhancer–promoter interactions from the MYB-binding site compared to the reference allele (Extended Data Fig. 2d,e).

In conclusion, we found that single-allele analysis of chromatin architecture with MCCv, using both short-range nanoscale maps of the *cis*-regulatory element and long-range enhancer–promoter contacts, minimizes experimental noise through simultaneous capture of variant alleles in the cell population and enables noncoding variants that alter chromatin architecture to be identified.

### Identification of disease-associated genes in human CD4^+^ T cells

**Fig. 2:**
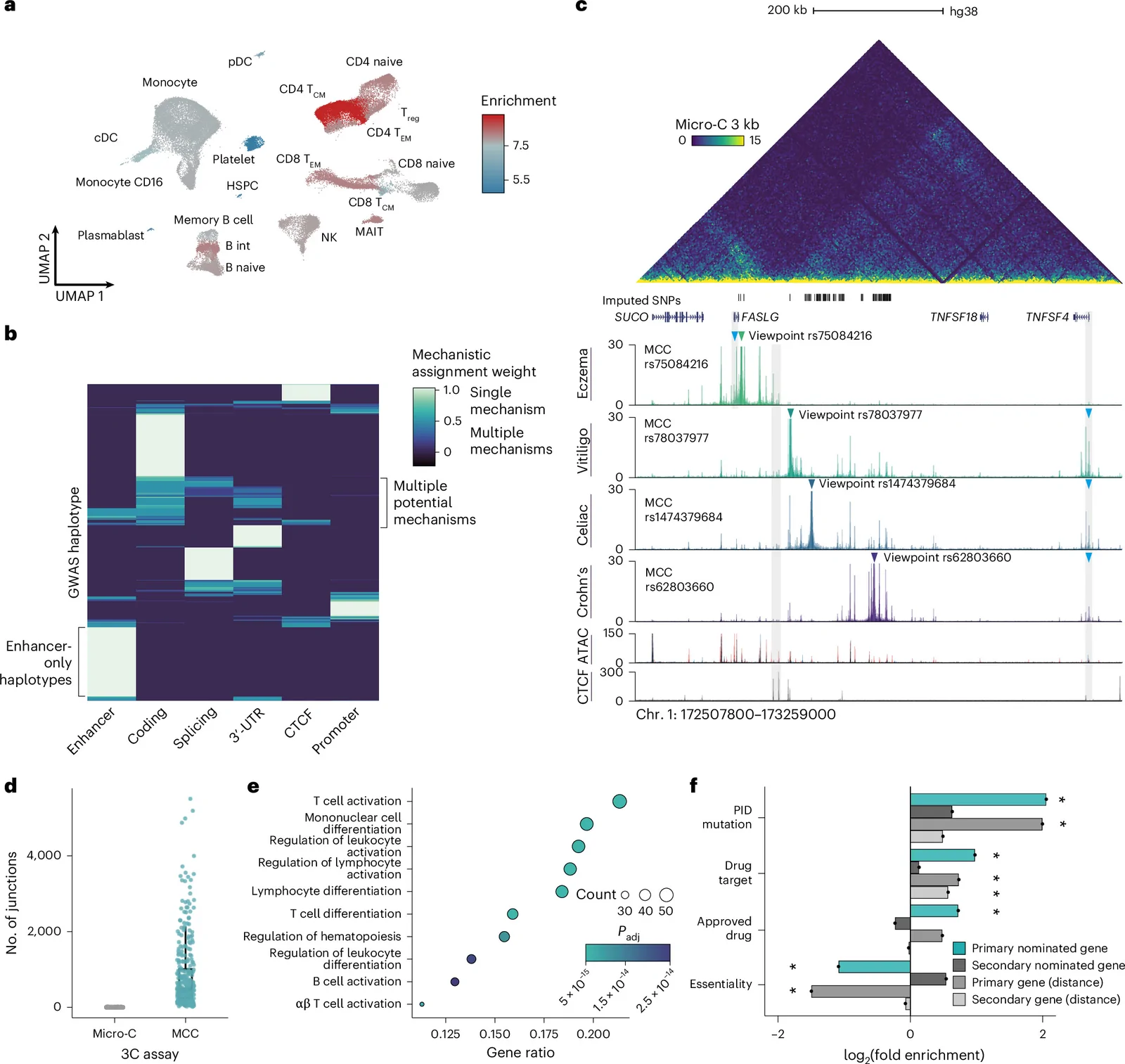
Linking GWAS variants to genes using MCC. **a**, Uniform Manifold Approximation and Projection (UMAP) of scATAC–seq from peripheral blood, colored by enrichment of lead SNPs in open chromatin peaks per cluster^54^. **b**, Clustered hierarchical heatmap for haplotypes with at least one molecular mechanism assigned. Mechanistic assignment weight = 1/(number of distinct candidate mechanisms among SNPs in the haplotype). **c**, Micro-C from CD4^+^ T cells (3-kb bin size; pooled from three replicates), ATAC–seq (CD4^+^, unstimulated blue, stimulated red, overlay) and CTCF ChIP–seq for the 1q24.3–25.1 locus: MCC for rs75084219 (eczema), rs78037933 (vitiligo), rs1474379684 (celiac disease) and rs62803660 (Crohn’s disease) with SNP–promoter contacts highlighted in gray (3 biological replicates, each with 6 technical replicates (18 in total)). **d**, Comparison of the number of variant–promoter ligation junctions identified by MCC (median = 828.2) and Micro-C (median = 2.07) (*n* = 349 promoter contacts measured across 2 independent libraries for both methods, median (center line), 25th and 75th percentiles (box limits) and whiskers extending to the most extreme values within 1.5× the interquartile range). **e**, Gene ontology overrepresentation analysis of MCC-prioritized genes in CD4^+^ T cells (top ten pathways by gene ratio are shown (total significant hits = 251)). The gene ratio is defined as the number of genes in the input list that are annotated to the corresponding term (function) over the number of genes in the input list. **f**, Comparison between the enrichment of genes with the highest number of MCC contacts (primary nominated gene) versus genes with the second highest number of MCC contacts (secondary nominated gene) compared to background (all genes ±1 Mb from captured variants). For reference, the enrichment scores of the nearest and second nearest genes for each variant are also shown (essentiality (Depmap), drug target and approved drugs (open targets) and primary immunodeficiency (Genomics England)) (*χ*^2^ test, two-sided *P* values reported in Source data). cDCs, conventional dendritic cells; MAIT, mucosal-associated invariant T cells; NK, natural killer cells; pDCs, plasmacytoid dendritic cells; T_CM_, central memory T cells; T_EM_, effector memory T cells; T_reg_, regulatory T cells; B int, intermediate B cells. Source data

**Extended Data Fig. 3:**
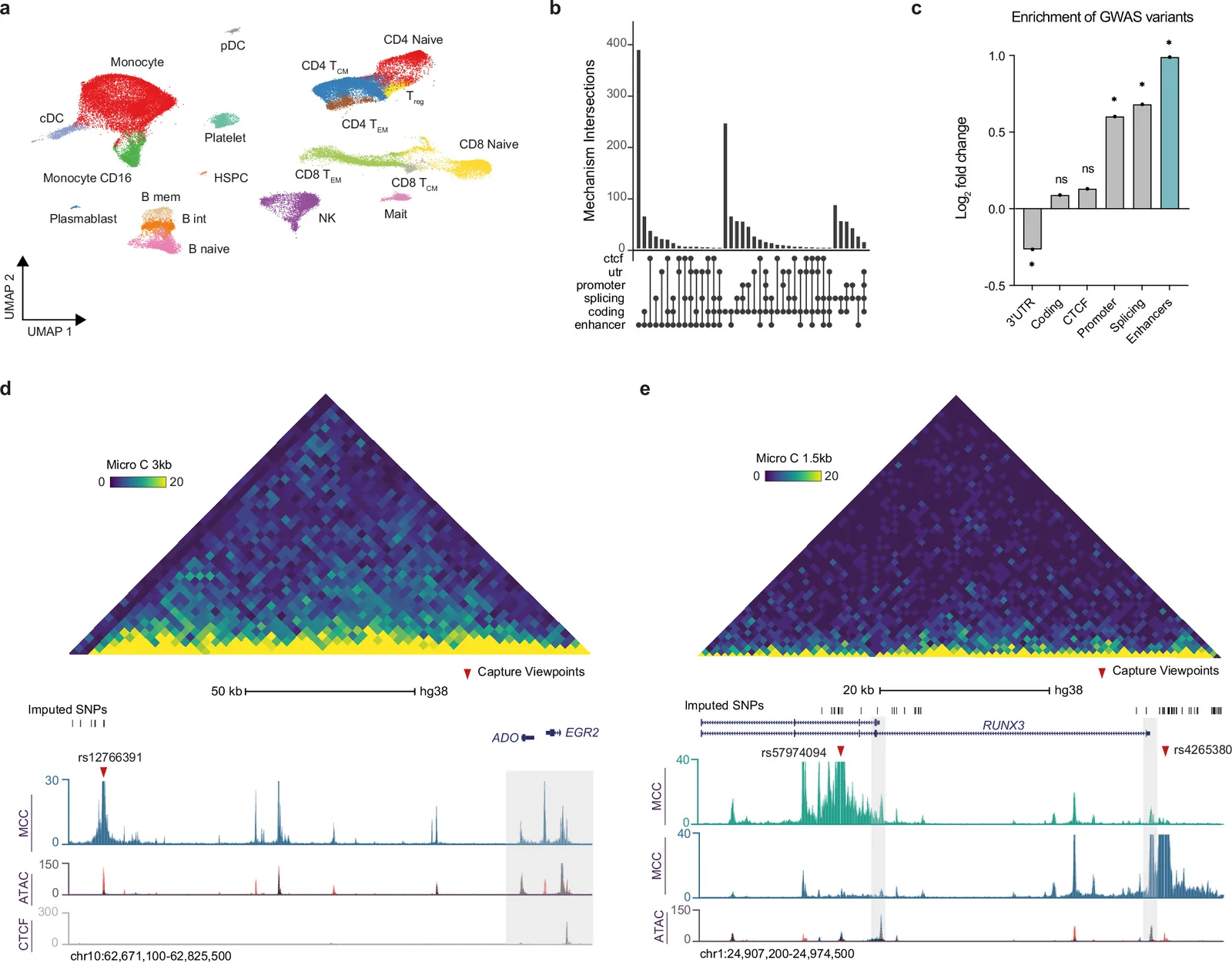
MCC can interpret previously intractable loci. **a**, Uniform Manifold Approximation and Projection (UMAP) of single-cell ATAC sequencing from peripheral blood. **b**, Upset plot of SNP prioritisation pipeline output. A total of 3238 IMID-GWAS signals were analysed, with 1441 having at least 1 SNP being identified in the six molecular mechanisms tested (coding, splicing, enhancer, CTCF, promoter, UTR). A total of 727 were classified as CTCF altering or enhancer altering in activated CD4^+^/CD8^+^ T cells or Monocytes. **c**, Enrichment scores of IMID-associated SNPs for each molecular mechanism tested in the SNP prioritisation pipeline (n = 124,000) compared to dbSNP151 common variants across the genome (n = 15,000,000). Enrichment scores were calculated as: the fraction of GWAS variants that can be explained by the tested molecular mechanisms over fraction of total common variants explained by the tested molecular mechanism (Χ^2^ test, two-sided, 3’UTR (*P* = 0.001), coding (*P* = 0.145), CTCF (*P* = 0.203), promoter (*P* = 3.68 × 10^−10^), splicing (P = 7.97 × 10^−13^, enhancer (4.67 × 10^−53^)). **d**, 3 kb binned Micro-C, ATAC (CD4^+^, unstimulated blue, stimulated red, overlay) and CTCF ChIP-seq for the 10q21.3 locus with MCC from rs12766391. **e**, 1.5 kb binned Micro-C, ATAC (CD4^+^, unstimulated blue, stimulated red, overlay) and CTCF ChIP-seq for the *RUNX3* locus with MCC from rs57974094 (green) and rs4265380 (blue). cDCs, conventional dendritic cells; MAIT, mucosal-associated invariant T cells; NK, natural killer cells; pDCs, plasmacytoid dendritic cells; T_cm_, central memory T cells; T_em_, effector memory T cells; T_reg_, regulatory T cells; B int, intermediate B cells. Source data

To validate MCCv as a tool for understanding human genetics, we investigated genetic variants associated with immune-mediated inflammatory diseases (IMIDs), which are chronic, largely incurable conditions that affect 5–7% of the population^27^. We performed enrichment analysis of GWAS signals in single-cell (sc)ATAC–seq, which showed that CD4^+^ T cells (particularly central memory T cells) contain the highest burden of IMID-associated genetic variation (Fig. 2a and Extended Data Fig. 3a). Next, we imputed 3,104 independent haplotypes from 400 studies and then tested each imputed variant within the haplotype for multiple molecular mechanisms (Supplementary Fig. 1a and Supplementary Table 1). This identified a potential causal variant and molecular mechanism for 47% of haplotypes, including 405 unique enhancer variants (Fig. 2b, Extended Data Fig. 3b and Supplementary Fig. 1b). IMID-associated variants were most strongly enriched for putative enhancer variants (Extended Data Fig. 3c). MCCv was performed at potential enhancer and a subset of CTCF variants, using oligonucleotide viewpoints designed precisely over the site of the variants, in activated primary CD4^+^ T cells. To benchmark the assay, we also performed genome-wide Micro-C^21,28^.

We were able to map previously challenging loci, such as the *EGR2* locus (Extended Data Fig. 3d), where two promoters are separated by an intervening enhancer element within 3 kb. Here the MCC data show preferential contact from the disease-associated enhancer to the promoter of *EGR2*. We found that 19.4% of enhancers had their nearest promoter contact falling within the resolution cut-off of conventional 3C techniques (<10 kb). For example, in the *RUNX3* locus we showed that two independent autoimmune signals map to two different *RUNX3* enhancers and contact different alternate promoters forming small-scale structures that are not defined by lower-resolution 3C techniques (Extended Data Fig. 3e).

**Extended Data Fig. 4:**
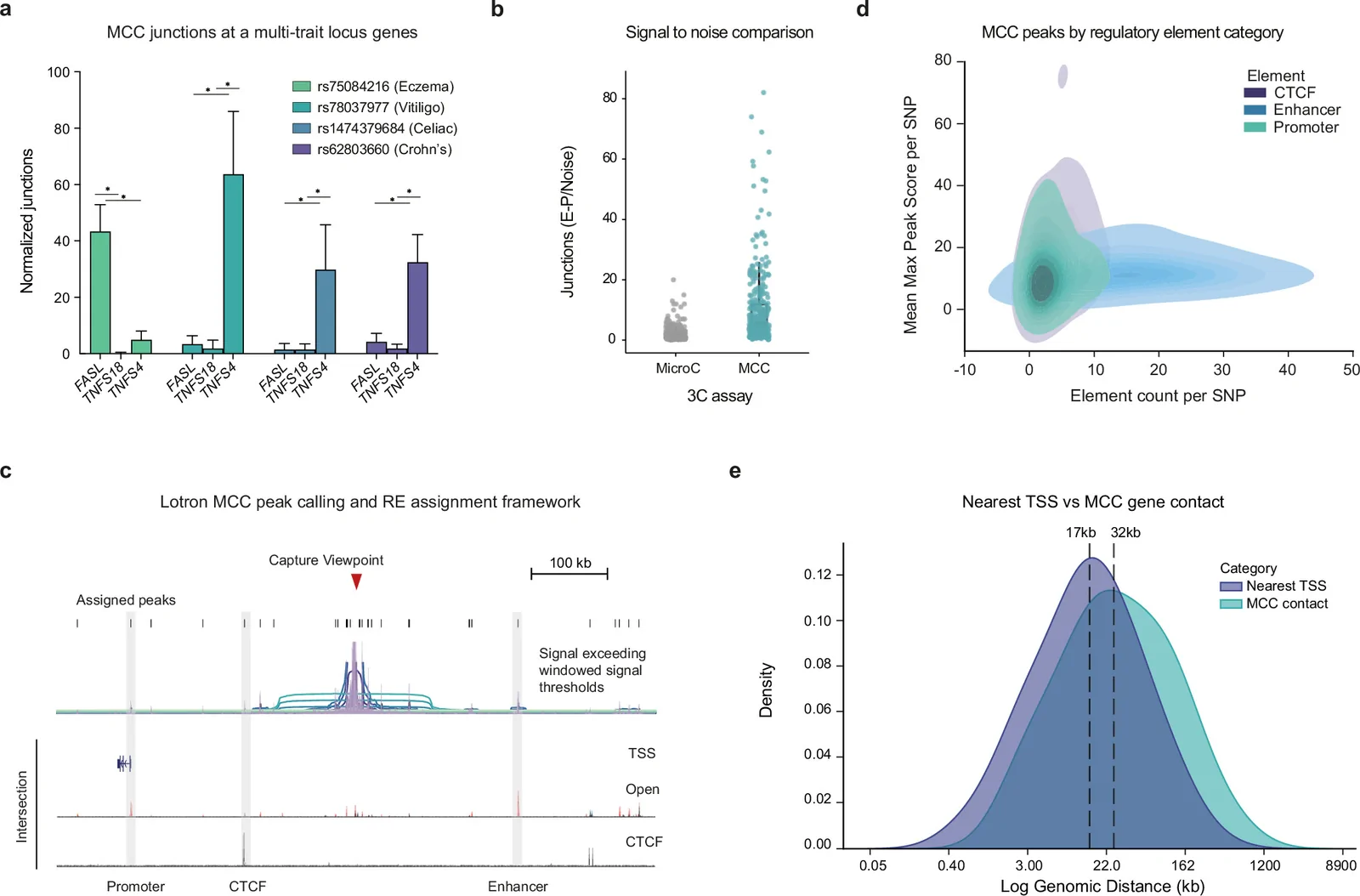
Further characterisation of MCC derived regulatory contacts at GWAS loci. **a**, Quantification of normalized MCC junctions at the genes in the multi-trait locus for all variants shown in Fig. 2c (n = 18 (3 donors, with 6 independent MCC libraries each), mean ± s.d., paired Student’s t-test, two-sided, two-stage step-up method for multiple test correction, *P* values reported in source data). **b**, Enrichment of variant–promoter ligation junctions compared to background between Micro-C (median = 1.33) and MCC (median = 5.5). Calculated using variant–promoter contacts in Fig. 2d divided by the mean junction counts at random locations that did not overlap peaks within the bounds of the two most extreme MCC peaks either side of the capture oligo (n = 349 promoters contacts measured across 2 independent libraries for both methods, median (centre line), 25th and 75th percentiles (box limits), and whiskers extending to the most extreme values within 1.5 × the interquartile range). **c**, Schematic of LotronMCC peak calling including how it assigns peaks and classifies them into regulatory element categories. For each viewpoint, 27 individual signal thresholds are computed comprised of the rolling mean coverage of MCC signal or background (with varying windows), or the fold increase of signal over background (with varying thresholds). A peak is called if a region exceeds 10 out of 27 signal thresholds. Peaks are assigned as promoters based on open chromatin and transcription start site intersection. CTCF peaks are assigned on open chromatin and CTCF ChIP-seq intersection. Remaining open chromatin intersections are assigned as enhancers. **d**, 2D KDE plot of average Lotron MCC peak score for different regulatory element types (promoter in green, enhancer in blue, and CTCF in purple) per SNP versus the corresponding number of those element types forming MCC peaks with that SNP. **e**, KDE plot showing the distance between the MCC prioritised promoter and corresponding SNP (median = 32 kb) compared to the nearest promoter to that SNP (median = 17 kb). Source data

At the 1q24.3–25.1 locus we mapped four independent GWAS signals from IMIDs to four distinct regulatory elements (Fig. 2c). The eczema-associated signal intersects with an enhancer that contacts *FASL*, whereas the vitiligo-associated, celiac disease-associated and Crohn’s disease-associated enhancers contact *TNFSF14*, skipping the intervening *TNFSF18* promoter (Extended Data Fig. 4a). We were unable to detail the diverging contacts at this locus using our Micro-C dataset due to the sparsity of the data (Fig. 2c). Across all 405 SNPs, we found that the strongest enhancer–promoter contact was supported by a mean of 839 ligation junctions in the MCC datasets, whereas Micro-C identified an average of 2.5 junctions (Fig. 2d). Furthermore, MCCv had a fivefold greater signal-to-noise ratio compared to the Micro-C dataset (Extended Data Fig. 4b).

To facilitate high-throughput analysis of the data, we developed LotronMCC, a peak-calling algorithm specifically for MCC datasets, which simultaneously classifies the peaks into distinct *cis*-regulatory element types (Extended Data Fig. 4c)^29^. Many variants contacted multiple promoters (Extended Data Fig. 4d) (mean = 2.81 per SNP). We ranked promoters based on the frequency of MCC contact, allowing us to nominate 251 unique causal genes (primary nominated gene). This identified that the strongest contact frequencies from 60.5% of variants link to the nearest gene (Extended Data Fig. 4e). Gene ontology analysis identified that the primary nominated genes cluster with key immune pathways (Fig. 2e) and were also enriched for rare primary immunodeficiency mutations and tractable drug targets (Fig. 2f). Of primary MCC nominated genes, 56% were similarly prioritized by OpenTargets L2G^30^.

### Single-allele analysis of chromatin structure identifies functional variants in disease-associated regulatory elements

**Fig. 3:**
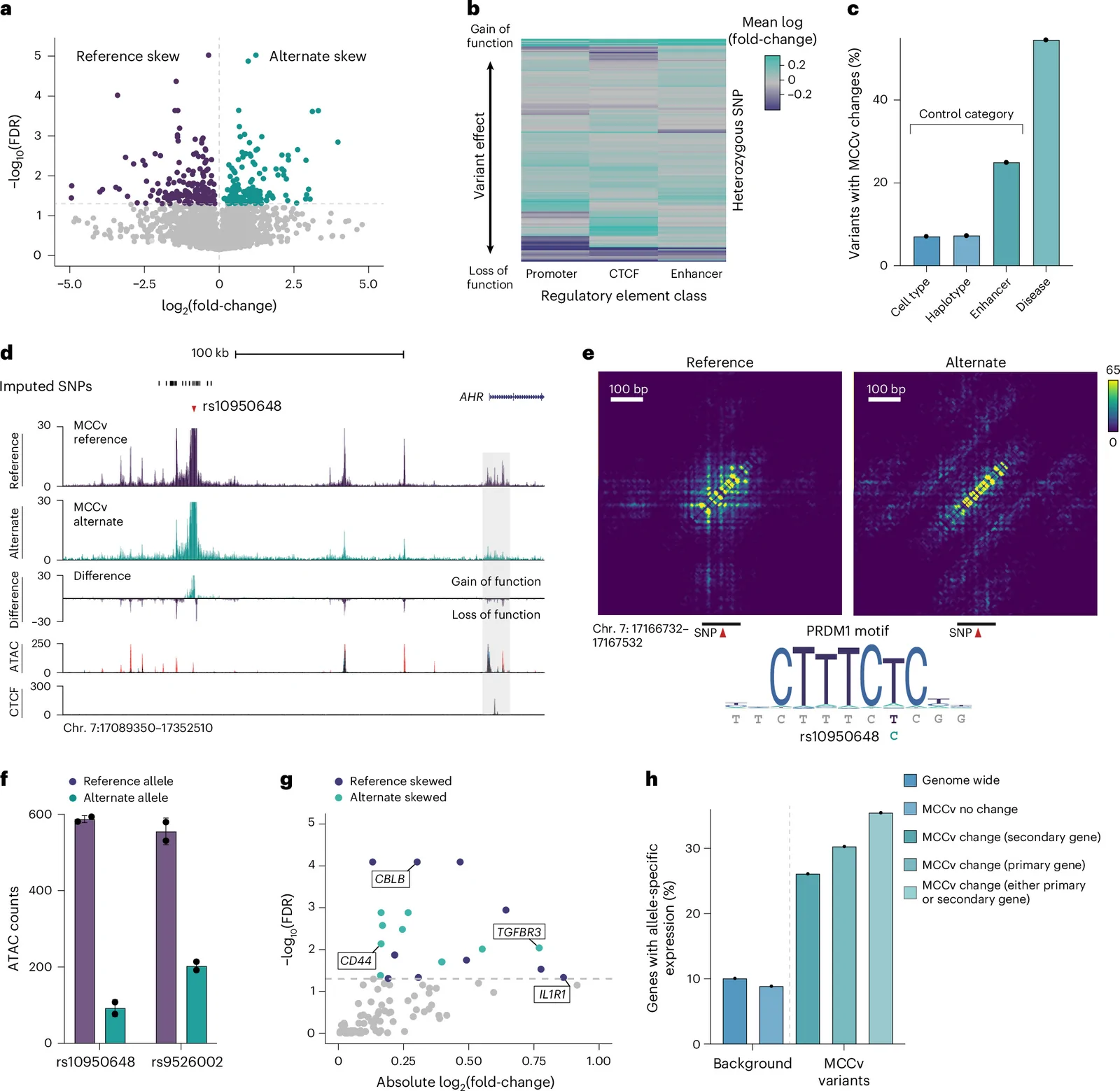
MCCv identifies GWAS variants that alter chromatin architecture. **a**, Alternate or reference normalized junction counts at MCCv peaks for heterozygous SNPs (*n* depends on how many donors are heterozygous for the SNP from which the peak is derived (minimum of six independent libraries per donor), paired, two-sided Student’s *t*-test, local false discovery rate (FDR) estimation). **b**, Clustered hierarchical heatmap of the average ratio of alternate to reference junction counts for peaks overlapping different regulatory element categories per SNP. **c**, Percentage of variants with significant MCCv changes (two-sided *χ*^2^ test, disease variants *n* = 227). Enrichment over controls (enrichment, *P*, *n* control): cell type = IMID variants intersecting monocyte enhancers (7.5, 0.0015 and 14); haplotype = SNPs in haplotype that do not intersect CD4^+^ T cell enhancers (7.5, 2.73 × 10^−13^ and 82); enhancer = variants that intersect CD4^+^ T cell enhancers but are not associated with IMIDs (2.2, 0.000014 and 76). **d**, MCCv from rs10950648 with SNP–promoter contact in gray. Difference plots (alternate minus reference), ATAC–seq (CD4^+^, unstimulated blue, stimulated red) and CTCF ChIP–seq shown underneath. **e**, Nanoscale contact maps of rs10950648 with 10-bp directional windowing. They are mirrored on the diagonal. PRDM1 motif and enhancer sequences are shown with the effect of the alternate allele highlighted (*n* = 12, merged *β* = -1.09). **f**, ATAC–seq read skew for rs10950648 (*q* = 0.024) and rs9526002 (*q* = 0.025) (*n* = 2 donors, mean ± s.d.). **g**, Allelic expression of primary or secondary nominated genes phased to a heterozygous variant: 88 variants (102 variant–gene pairs) tested. Twenty-three genes showed donor-level skew (variable-phased SNPs (*n*) per gene; two-sided, Wilcoxon’s signed-rank test was used per donor (Fisher’s method across donors) with the Benjamini–Hochberg correction). **h**, Percentage of allelically imbalanced genes by SNP–gene categories. Backgrounds were genome-wide allelic gene imbalance, 10.0% (231 out of 2,313) and allelic gene imbalance without MCCv skew, 8.9% (17 out of 190). The percentage of genes showing allelic imbalance linked to variants with evidence of MCCv changes (category: *n* variant-–gene pairs, enrichment over genome-wide (*p*), enrichment over MCCv (*p*); secondary genes: 6 of 23, 2.61 (0.0316); primary genes 10 of 33, 3.03 (0.0316), 3.40 (0.038); primary or secondary: 16 of 45, 3.55 (0.0001), 3.99 (0.0002) (two-tailed *χ*^2^ test). Source data

**Extended Data Fig. 5:**
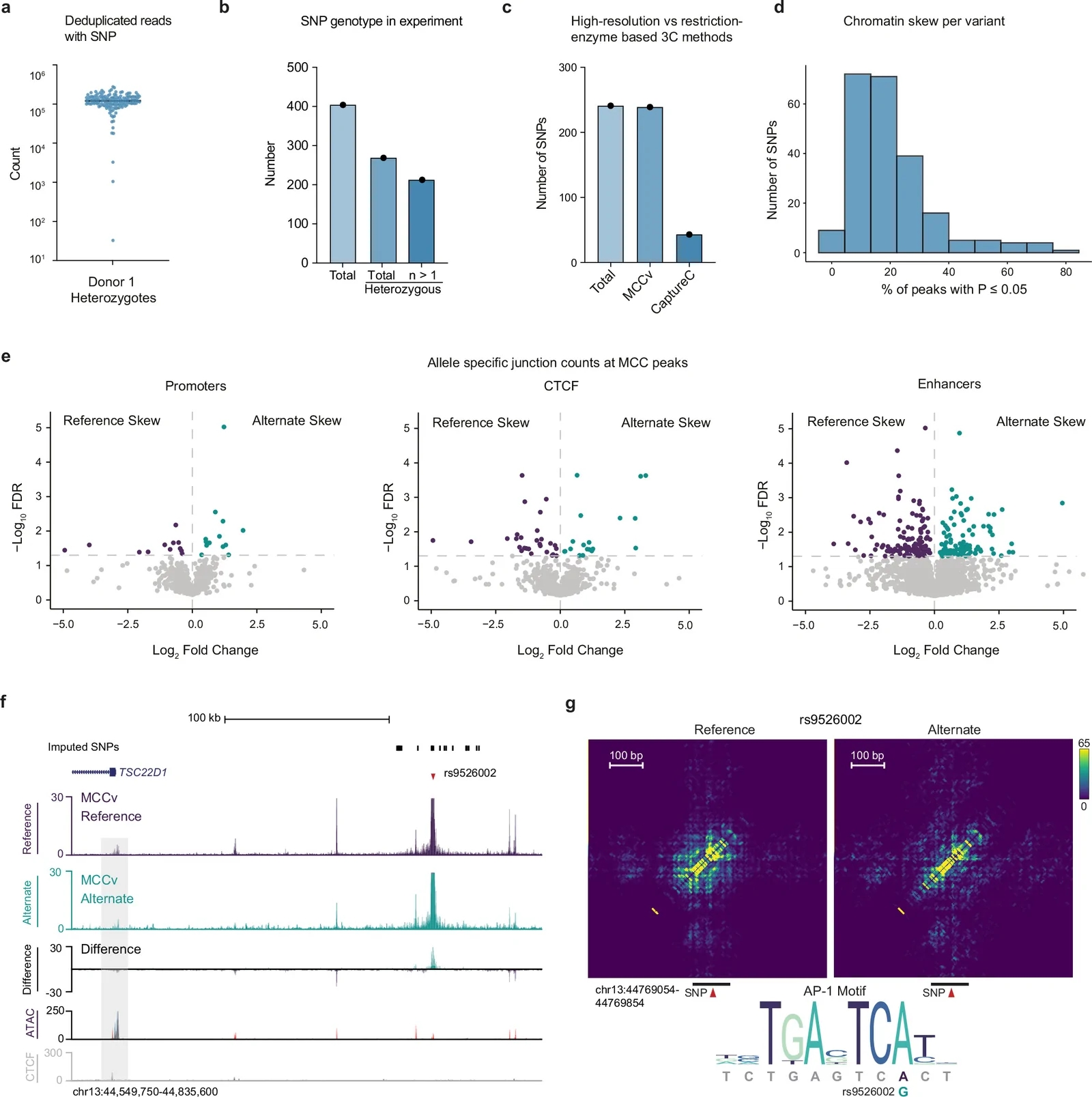
MCCv identifies regions of 3D genome skew in primary cells. **a**, Count data for PCR deduplicated reads containing the target SNP for a subset (donor 1 only) of heterozygous SNPs (n = 147 variant read counts from 6 independent MCC libraries from a single donor). This data is used to variant call the genotype of captured GWAS sites before applying the MCCv single-allele analysis pipeline. **b**, Summary of the number of variants captured by MCC (total) and those amenable to allelic analysis (heterozygous). **c**, Visualisation of the total number of heterozygous variants and the breakdown of those where allelic data was successfully generated using MCCv. We also include a theoretical estimation of the number of variants that could generate allele-specific data in a dpnII-based 3 C experiment (for example Capture C), where variants had to fall within 60 bp of a dpnII-cut site to be classified as successful. **d**, The distribution of SNPs in the MCCv dataset in terms of percentage of peaks which are significantly altered between alleles (relative to the total number of MCCv peak for that SNP). **e**, as in Fig. 3a but data has been filtered for promoters, CTCF, or enhancer elements before plotting. **f**, MCCv profile for rs9526002, with SNP-promoter contact highlighted in grey. Difference plots show alternate-minus-reference contacts, with CD4^+^ ATAC-seq and CTCF ChIP-seq tracks below. **g**, rs10950648 nanoscale contact maps generated using 10-bp directional windowing and mirrored on the diagonal. The AP-1 motif and enhancer sequence are shown, with the effect of the alternate allele highlighted (n = 12 (2 donors, 6 independent MCC libraries each), merged β = −1.10). Source data

To identify disease-associated variants that alter chromatin architecture, we applied the single-allele capabilities of the MCCv platform to this dataset. MCCv generates ~10^5^ PCR duplicate-filtered reads directly over the variant of interest, which allows high-confidence variant calling of heterozygous SNPs for allele-specific analysis (Extended Data Fig. 5a). MCCv analysis of 405 *cis*-regulatory elements produced phased tracks from 241 heterozygous sites across 3 donors (Extended Data Fig. 5b). We estimated that only ~30% of these SNPs could be analyzed with conventional 3C-based approaches (Extended Data Fig. 5c). MCCv revealed that 54.6% of heterozygous disease-associated variants have at least one significantly altered contact (Fig. 3a and Extended Data Fig. 5d,e) and both loss-of-function and gain-of-function variants were identified (Fig. 3b). Disease-associated SNPs showed significantly greater changes in MCCv allele-specific, 3C contacts than control SNPs for cell type, haplotype and disease association (Fig. 3c).

We went on to undertake analysis of variants at individual loci. We identified that rs10950648 (associated with ankylosing spondylitis and Crohn’s disease^31^) interacts with the aryl hydrocarbon receptor (*AHR*) promoter and adjacent enhancer elements more strongly on the reference allele compared to the alternate allele (Fig. 3d,e and Supplementary Fig. 2a). We also analyzed a psoriasis-associated variant (rs9526002), in which the protective variant results in reduced contacts with the promoter of *TSC22D1* (Extended Data Fig. 5f,g and Supplementary Fig. 2b).

**Extended Data Fig. 6:**
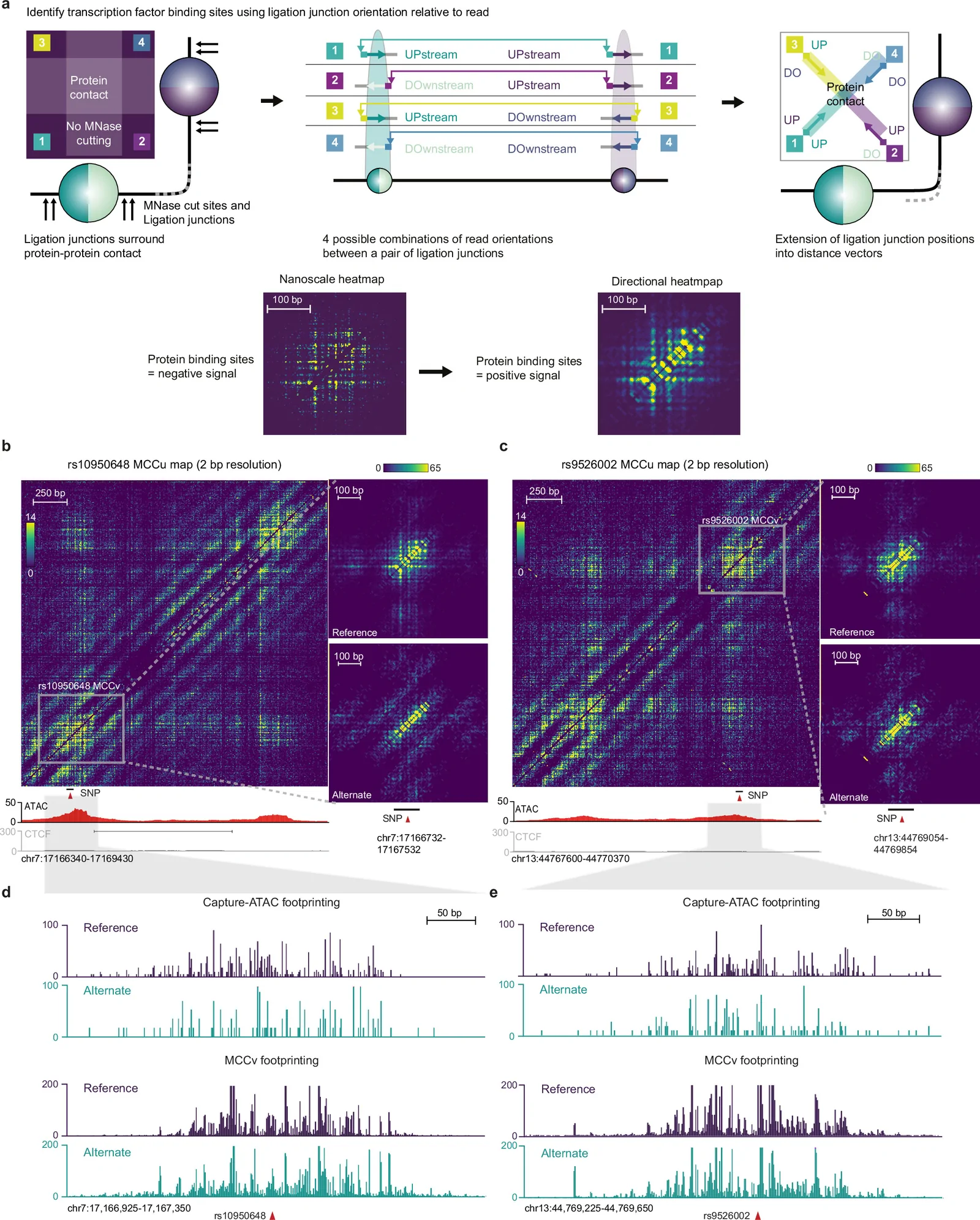
Further visualisation of nanoscale chromatin architecture at disease-associated enhancers. **a**, Schematic of converting nanoscale heatmaps into directionally windowed heatmaps for the visualisation of contacts between transcription factor motifs. Reads that give rise to ligation junctions are thought to be protected from MNase cutting by bound transcription factors. The direction of the extension of the reads can be leveraged to estimate the position of the putatively bound transcription factor. This converts the negative signal of the transcription factor footprint into positive signal equating to potential contacts between transcription factors. **b**, MCC ultra tile over the rs10950648-containing enhancer (n = 6 independent MCC libraries, bin size = 2, tile = 2.2 kb). Corresponding ATAC (CD4^+^, unstimulated blue, stimulated red, overlay) and a unified CTCF track are shown underneath. The capture probe/SNP locations are shown by the horizontal bar and red triangle respectively. Zoom in shows MCCv single-allele directional windowed heatmaps for reference and alternate alleles at the variant shown in Fig. 3e. **c**, As in **b** for the rs9526002-containing enhancer with zoom in corresponding to directional windowed heatmap as shown in Extended Data Fig. 5g (n = 6 independent MCC libraries, bin size = 2, tile = 1.9 kb). **d** and **e**, Footprinting data from ATAC-seq combined with MCCv based capture enrichment (above) and 1D-MCCv footprinting (below) for both reference and alternate alleles of rs10950648 and rs9526002. SNP position is indicated with the red triangle.

To aid visualization of the nanoscale chromatin architecture at these sites, we utilized a contact sequence reconstruction approach leveraging the directionality of ultra-short-range ligation junctions and performed MCC ultra^32^ to place this within the context of its surrounding chromatin structure (Extended Data Fig. 6a–c). This approach revealed putative interactions between potential transcription factor-binding sites on the reference alleles, whereas the disease-associated variants on the alternate alleles showed a nucleosomal striping pattern, likely due to the loss of PRDM1 (Fig. 3e) and AP-1 (Extended Data Fig. 5g) binding, for rs10950648 and rs9526002, respectively. We compared our 3D reconstruction approach with conventional MCC footprinting and found that the 3C component was important for delineating variant-associated differences. We went on to develop an approach combining targeted capture and ATAC–seq to provide deep ATAC–seq data (>1,000 PCR-deduplicated reads per variant) for footprinting (Extended Data Fig. 6d,e). This deep sequencing of ATAC–seq footprinting data was largely uninformative even if the variants demonstrated evidence of ATAC–seq read skewing (Fig. 3f).

**Extended Data Fig. 7:**
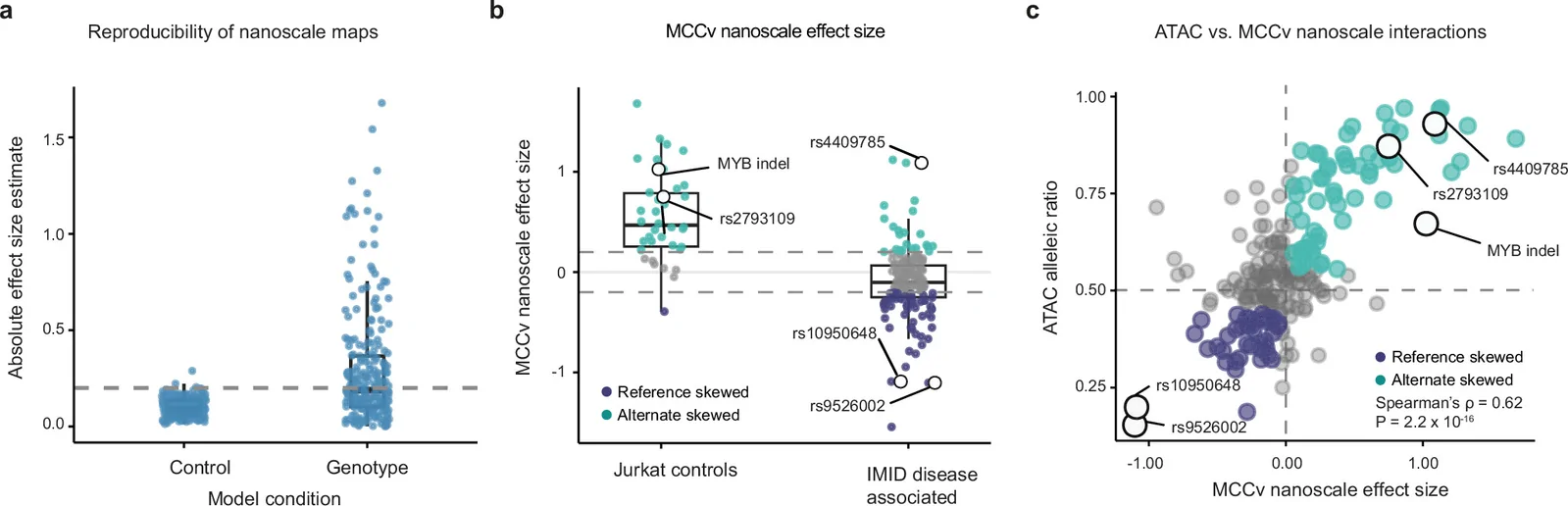
Quantifying the impact of disease-associated variants on nanoscale chromatin architecture. **a**, Effect size estimate of GLM from basepairC at tested SNPs (n = 231) (bottom 20% of variants based on read counts were filtered). Estimates were done between maps of different genotypes (allele-specific) (Genotype, mean β-coefficient (effect size) = 0.293). Random combinations of 6 reference and 6 alternate allele sample matrices were compared (mean β-coefficient = 0.097) (median (centre line), 25th and 75th percentiles (box limits), and whiskers extending to most extreme values within 1.5 × interquartile range). **b**, Effect size from basepairC matrix difference modelling in Jurkat controls (prioritised using ATAC skew) vs. T cell disease associated enhancers (prioritised by ATAC intersection only). Variants within lowest 10% of read counts for MCCv nanoscale maps were filtered from the correlation. Gain-of-function (MCCv nanoscale effect size ≥ 0.2) and loss-of-function (MCCv nanoscale effect size ≤ -0.2) variants are highlighted in green and purple respectively. Variants highlighted throughout the study are indicated on the plot (Jurkat controls – n = 38 variants (6 independent MCC libraries), IMID variants – n = 191 variants (6 independent MCC libraries from 1-3 biological donors, median (centre line), 25th and 75th percentiles (box limits), and whiskers extending to the most extreme values within 1.5 × the interquartile range.). **c**, Allelic ratio from ATAC-seq versus effect size from basepairC matrix difference modelling (n = 201, two-sided Spearman’s ρ = 0.62, *P* = 2.2 × 10^−16^). Variants within the lowest 10% of read counts for either ATAC or MCCv were filtered from the correlation. Nominally gain-of-function (ATAC allelic ratio ≥ 0.55 & MCCv nanoscale effect size ≥ 0.05) and loss-of-function (ATAC allelic ratio ≤ 0.45 & MCCv nanoscale effect size ≤ -0.05) variants are highlighted in green and purple respectively. Source data

We developed a statistical framework utilizing a generalized linear model to assess differences in basepair resolution matrices between alleles (basepairC). Using basepairC, we showed that differences in nanoscale patterns were largely driven by differences in genotype rather than between replicates or donors (Extended Data Fig. 7a and Supplementary Fig. 3). Of disease-associated variants, 47.6% had significant changes between alleles (absolute *β* coefficient (effect size) ≥0.2 and adjusted *P* value (*P*_ad__j_) ≤ 0.01) with both loss-of-function and gain-of-function effects consistent with MCCv long-range contact data (Extended Data Fig. 7b). Alterations in nanoscale patterns correlated with the degree of skew in ATAC–seq reads between alleles (Extended Data Fig. 7c).

Utilizing MCCv phasing, we analyzed allelic expression of primary and secondary nominated genes from heterozygous variants within intron-rich RNA-seq data from CD4^+^ T cells in matched donors. To improve the accuracy of identifying allelic skew within heterozygous donors, we employed strict filtering when phasing variant–gene pairs (Supplementary Fig. 4). This demonstrated changes in allelic expression of key mediators of immune signaling such as *CD44*, *TGFBR3* and *IL1R1* (Fig. 3g).

To ascertain whether changes in MCCv contacts enrich for functional consequences at the level of transcription, we estimated a genome-wide background allelic imbalance using the RNA-seq datasets and compared this to a variety of variant–gene pair conditions (Supplementary Note 1). This revealed that primary and secondary nominated genes linked to variants with detectable differences in MCCv contacts between alleles are significantly more likely to show allelic imbalance in RNA expression compared to the genome-wide background and to genes linked to variants without evidence of altered contact frequency (Fig. 3h). Overall, 35.5% (16 of 45) of variants with a change in MCC contacts were associated with RNA skew at either the primary or the secondary gene. For the primary genes linked to SNPs with changes in chromatin structure there was a threefold enrichment in detection of allelic RNA skew over the genome-wide background. There was directional concordance between the allelic imbalance and the nanoscale architectural changes at 64.7% of primary variant–gene pairs with reliable phasing and evidence of long-range MCCv changes. This rises to 88.2% when the directionality of the long-range MCCv changes is also considered.

Taken together, the single-allele analysis capabilities of MCCv increase mechanistic understanding of disease-associated noncoding variants and, when paired with phased RNA-seq, can associate enhancer genotypes to transcriptional outcomes.

### MCCv identifies a CTCF-mediated gain-of-function mechanism at a multitrait autoimmune locus

**Fig. 4:**
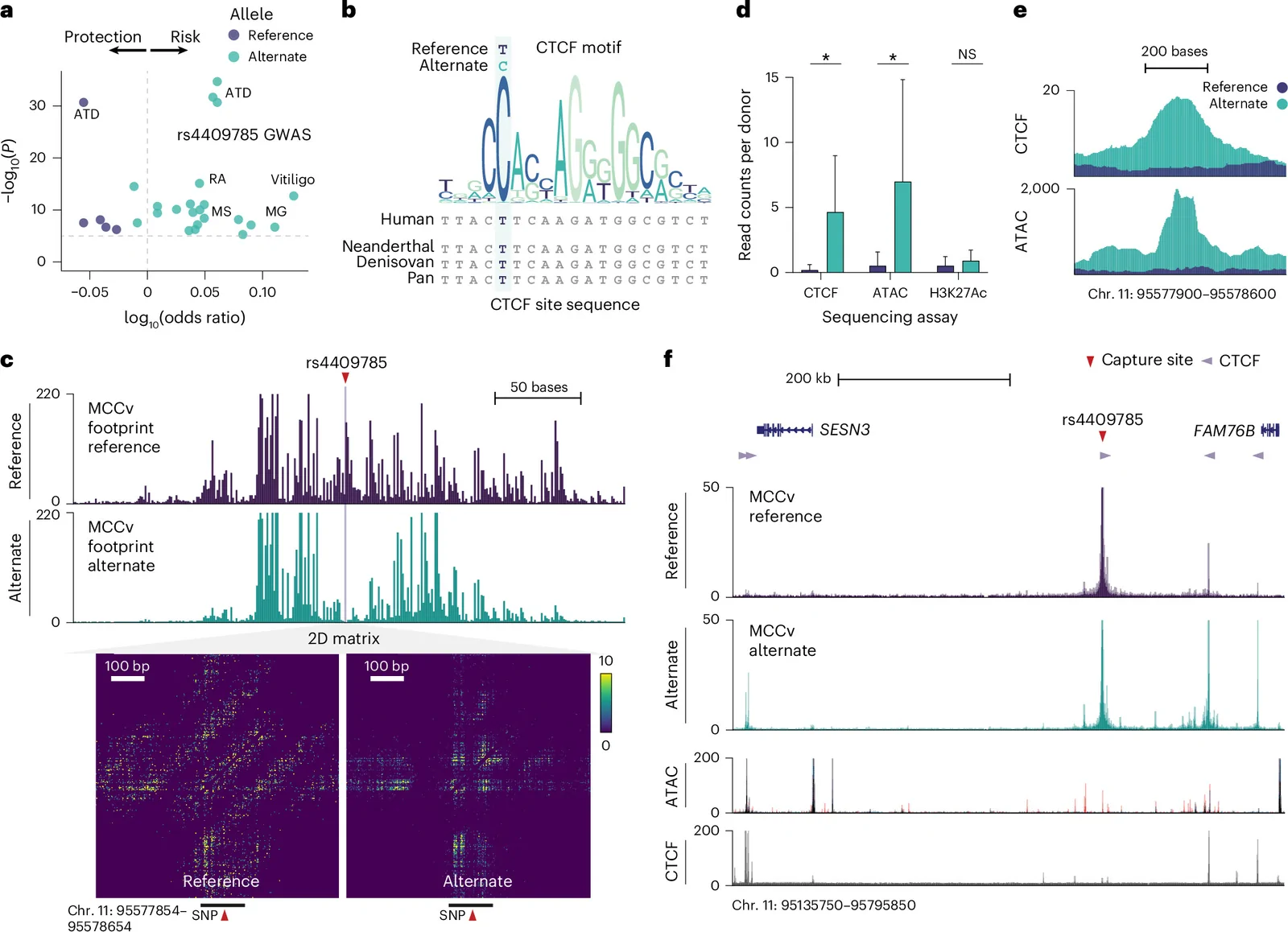
rs4409785 acts through a gain-of-function CTCF site. **a**, Associations of rs4409785 from the National Center for Biotechnology Information (NCBI) GWAS catalog, with −log_10_(*P*) versus log_10_(odds ratio) for the tested allele plotted as a volcano. Associations reported with reference T allele are shown in purple, whereas associations reported with the alternate C allele are shown in green. Representative autoimmune disease signals are labeled (autoimmune thyroid disease (ATD), RA, MS, myasthenia gravis (MG) and vitiligo). **b**, CTCF consensus motif and the sequence around rs4409785 in humans. The effect of rs4409785 is highlighted in gray. Sequence alignment from early hominins and chimps is shown below. **c**, MCCv basepair resolution footprinting for reference and alternate alleles with the rs4409785 location marked by a red triangle. Corresponding nanoscale heatmaps are shown as in Fig. 1d (bin size = 1 bp, 800-bp window, *n* = 6 merged per allele and merged *β* = 1.09). **d**, CTCF ChIP–seq (*q* = 0.000123), ATAC–seq (*q* = 0.000123) and H3K27ac ChIP–seq (*q* = 0.156) skew in heterozygous donors (*n* = 15 CTCF or ATAC, *n* = 12 H3K27ac; two-sided Wilcoxon’s matched-pairs, signed-rank test, two-stage step-up procedure for multiple-test correction, mean ± s.d.). **e**, CTCF ChIP–seq and ATAC–seq reads per kilobase of transcript per million mapped reads-normalized signal from three donors who are either homozygous reference (purple) or alternate (green) for rs4409785. **f**, MCCv profiles for rs4409785 alleles with corresponding ATAC (CD4^+^, unstimulated blue, stimulated red, overlay) and a unified CTCF track. Capture probe location or SNP is marked by the red triangle and CTCF elements with orientation are marked by purple triangles. Source data

**Extended Data Fig. 8:**
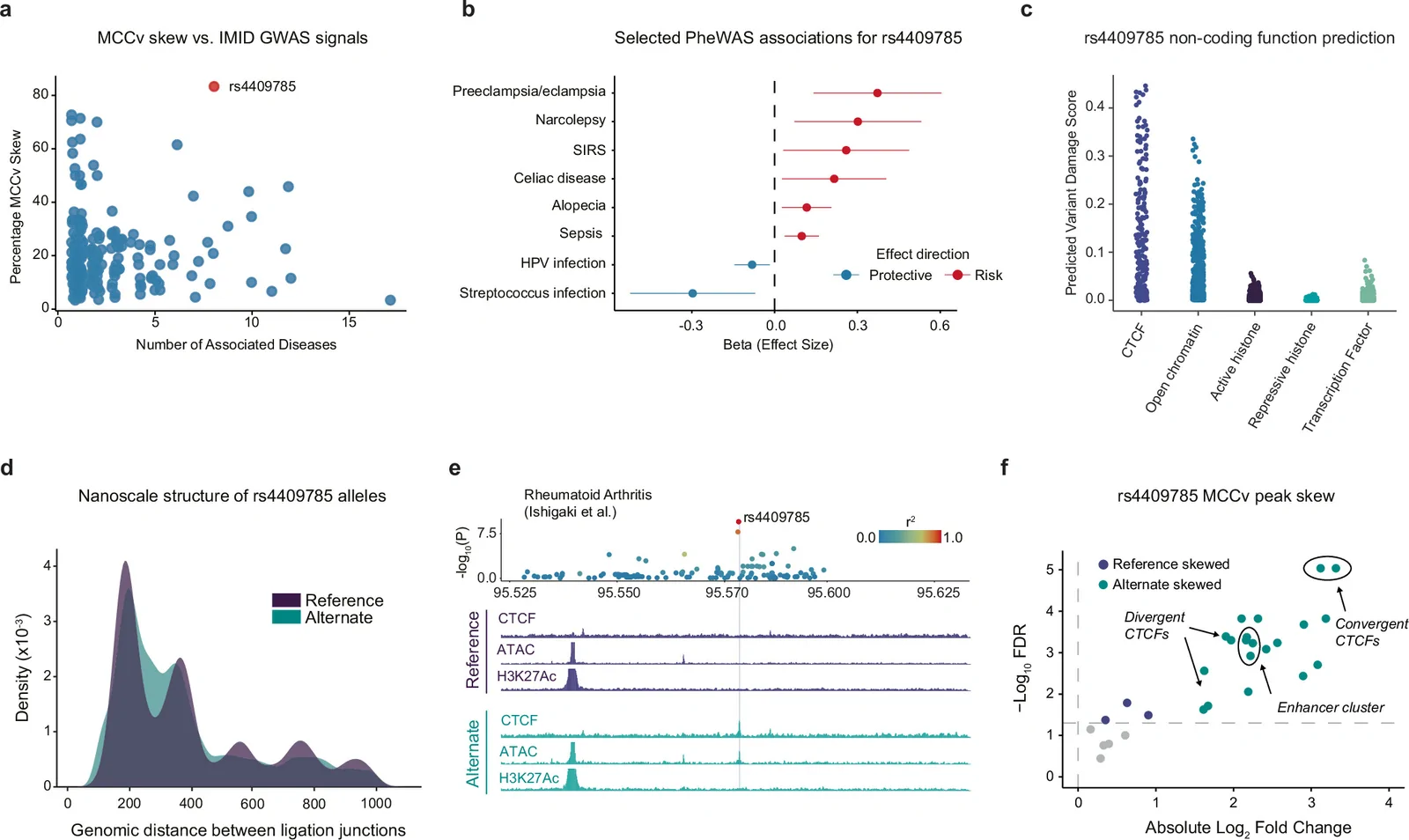
11q21 contains a pleiotropic autoimmune risk locus within a gene desert. **a**, Percentage MCCv skew (percentage of total peaks demonstrating skew) versus number of NCBI GWAS catalogue associations for all MCCv SNPs in dataset, with rs4409785 highlighted in red. **b**, Associations between rs4409785 and various immune-related traits in the All of Us database. Only hits with a significant FDR corrected *P* value are displayed (n cases/controls = 257/98802 (SIRS), 377 /98552 (Celiac), 1311/95544 (Alopecia), 436/83656 (Preeclampsia), 254/99186 (Narcolepsy), 4683/164303 (Sepsis), 4108/163806 (HPV), 260/98019 (Streptococcus infection), odds ratio ± standard error, Firth logistic regression, two-sided; Benjamini–Hochberg FDR correction) (SIRS = serious inflammatory response syndrome, HPV = Human papillomavirus). **c**, DeepHeam absolute damage score for each classifier grouped into molecular categories. Damage score calculated as the difference between reference and alternate allele predictions for tested epigenetic classifier. **d**, Locus zoom plot for the association of rs4409785 with rheumatoid arthritis. R^2^ values for nearby SNPs are displayed from high-linkage (red) to low-linkage (blue). The molecular effect of this variant is shown underneath by genomic tracks of CTCF ChIP-seq, ATAC-seq, and H3K27ac ChIP-seq from donors homozygous for either the reference (purple) or alternate risk (green) allele. **e**, KDE plot of primary genomic distance between two base-pairs which make up ultra-short ligation junctions ( < 800 bp) for rs4409785. **f**, Absolute log_2_ fold change of junction counts between reference and alternate alleles at called Lotron MCC peak for rs4409785 (n = 6, paired Student’s t-test, two-sided, local FDR estimate with fdrtools). Key CTCF peaks as well as the peaks belonging to the *SESN3* super-enhancer are highlighted as being significant. Source data

Through prioritization of haplotypes with both clear functional effects and clinical importance, we identified the rs4409785 variant at the 11q21 locus, which has associations with multiple autoimmune diseases including multiple sclerosis (MS) and rheumatoid arthritis (RA) (Fig. 4a and Extended Data Fig. 8a). Phenome-wide association analysis of the All of Us and UK Biobank databases identified further associations including risk for sepsis and protection against *Streptococcus* spp. and human papillomavirus infection (Extended Data Fig. 8b).

Motif analysis of the disease-associated allele demonstrated that it creates a neo-CTCF-binding motif (Fig. 4b) and deep learning methods^33,34^ prioritized this variant as having a high-impact gain of function (Extended Data Fig. 8c). Conservation analysis showed that the reference T allele is conserved throughout hominin and primate evolution, confirming gain of function with the risk allele (Fig. 4b and Supplementary Fig. 5).

MCCv showed marked changes in the nanoscale topology of the nucleosome-depleted region and a 20-bp footprint precisely over the CTCF motif only in the alternate risk allele (Fig. 4c and Extended Data Fig. 8d). This was confirmed with analysis of CTCF ChIP–seq and ATAC–seq from heterozygous donors, which showed increased CTCF binding and open chromatin on the alternate allele (Fig. 4d,e and Extended Data Fig. 8e). MCCv analysis of long-range contacts revealed that the alternate allele of rs4409785 makes major contacts with two convergent CTCF sites (Fig. 4f and Extended Data Fig. 8f).

### Combining MCCv with genome editing to demonstrate mechanism

**Fig. 5:**
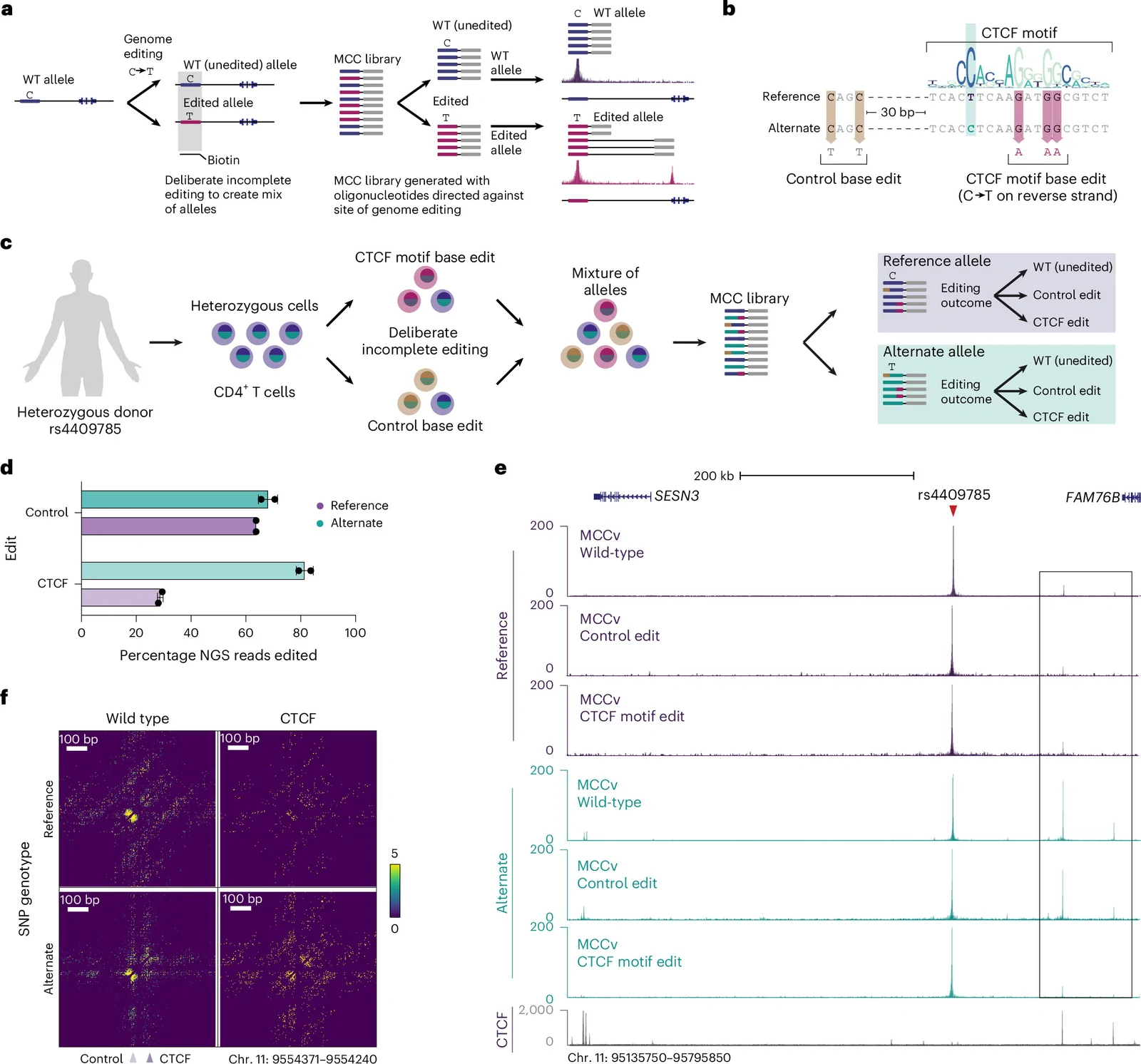
Direct read-out of genome editing with MCCv. **a**, Schematic illustrating coupling of basepair-level editing technologies with basepair resolution MCCv. Incomplete genome editing is leveraged to make a population of cells with mixed alleles become deconvoluted with the platform’s single-allele capabilities. **b**, Base editing experimental design with CTCF guide RNA sequence as well as locations of rs4409785 and the CTCF motif shown. The location of an upstream control guide outside the extended CTCF motif but within the MCC capture oligonucleotide is also shown. **c**, Experimental set-up of the CTCF and control edits in primary T cells. Edits were performed in parallel and mixed to avoid double editing. The cell mix was then fixed and captured by MCCv. As these cells were heterozygous for risk variant rs4409785 this led to six different editing outcomes that were deconvoluted by single-allele MCCv. To increase the depth of the data, six independent replicates were performed. **d**, Allele-specific (relative to rs4409785) editing efficiency of BE4max in primary CD4^+^ T cell for both control and CTCF guides. Editing efficiencies are shown as a percentage of NGS reads with C-to-T substitution. Where multiple Cs could be edited, the one with highest editing efficiency is shown (*n* = 2 independent experiments, mean ± s.d.). **e**, Edit-MCCv tracks for reference (purple) and alternate (green) alleles of rs4409785 with wild-type, control edit and CTCF edit shown. A CTCF ChIP–seq track is shown underneath. **f**, Nanoscale heatmaps for rs4409785 reference and alternate alleles with corresponding edit type (bin size = 1 bp, 800-bp window, *n* = 6 merged per allele). NGS, next-generation sequencing. Source data

We reasoned that MCCv could be used to create separate chromatin contact profiles after genome editing to delineate the effects of specific mutations on genome structure (Fig. 5a). We designed an editing strategy that could be undertaken in primary CD4^+^ T cells from individuals with heterozygosity for rs4409785, to definitively show that the alternate allele creates a CTCF-binding site at the 11q21 locus. We used the cytosine base editor BE4max to mutate three critical bases in the CTCF motif, but deliberately left the variant itself intact to allow the effects of editing the reference and alternate alleles to be studied separately (Fig. 5b). We also included a control guide (g)RNA, which targeted a region ~30 bp upstream of the extended CTCF-binding motif, to mitigate the effects of the genome editing itself on chromatin structure. All edits were deliberately designed to be within the region captured by the biotinylated oligonucleotide at the rs4409785 viewpoint. CD4^+^ T cells were harvested from a heterozygous donor for rs4409785 and split into two aliquots, which were nucleofected with either the control or CTCF-targeting gRNA to avoid cells with two simultaneous edits. Cells were subsequently mixed to minimize experimental noise and six technical replicates were performed to maximize data depth (Fig. 5c). Initially, we had to titrate down the base editor messenger RNA concentration to achieve ~50% editing efficiencies for both the CTCF-targeting and the control gRNAs (Fig. 5d).

**Extended Data Fig. 9:**
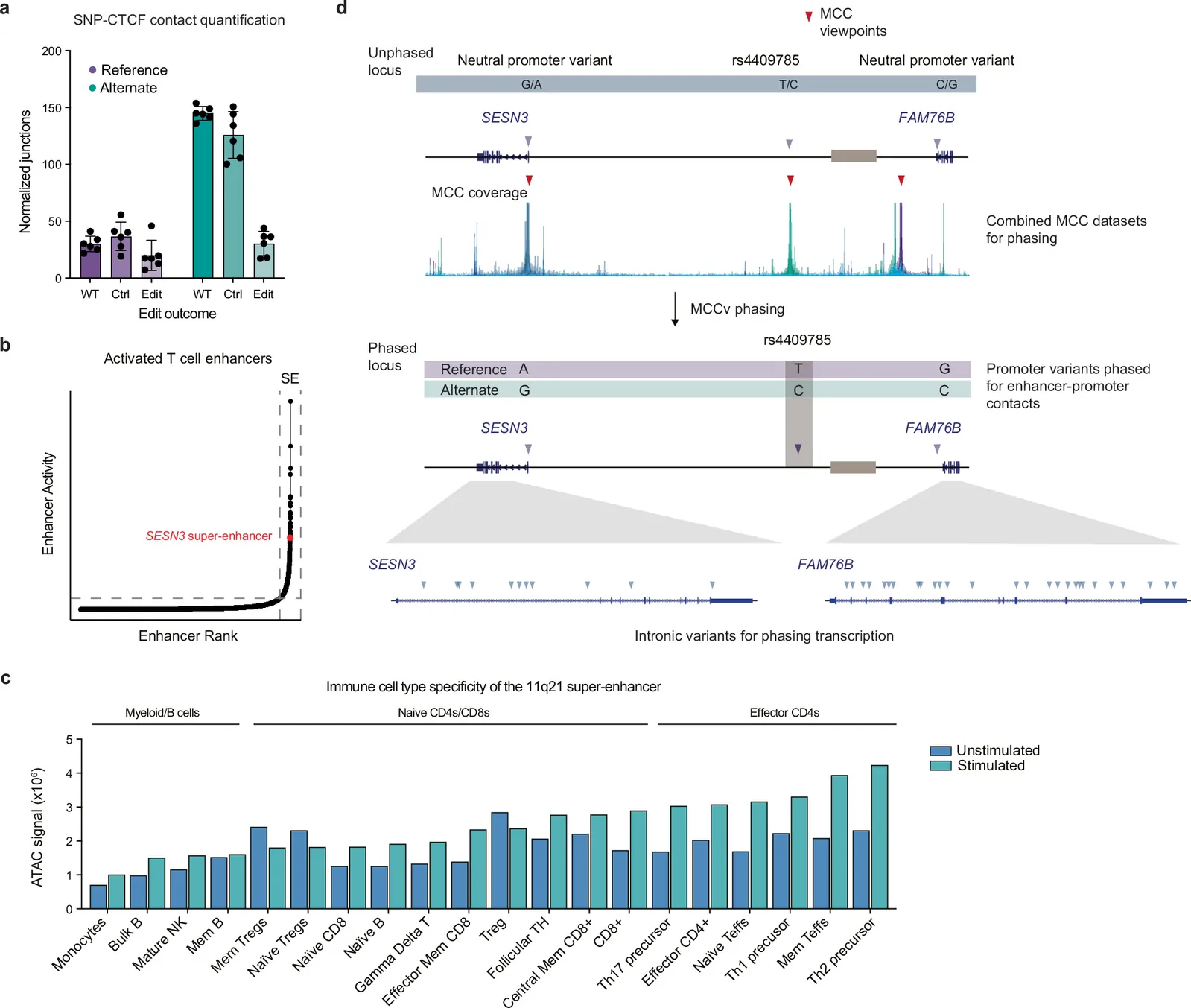
Phased genomics to decipher non-coding variant mechanisms. **a**, Ligation junction counts between rs4409785 and the major downstream CTCF site (wild type vs. edit; q = 0.089 (reference) and q = 0.000001 (alternate), wild type vs. control; q = 0.141 (reference) and q = 0.079 (alternate)) (n = 6 independent MCC libraries across 1 biological donor with 3 independent nucleofections, Student’s t-test, two-sided, two-stage step-up procedure for multiple test correction, mean ± s.d.). **b**, Detailed schematic for MCCv phasing strategy employed in order to analyse super-enhancer–promoter contacts and RNA in an allele-specific manner. Zoom-in shows the locations of phased intronic SNPs for both *SESN3* and *FAM76B* used in RNA skew analysis. **c**, Rank Ordering of Super-enhancers (ROSE) analysis of H3K27ac ChIP-seq data from activated CD4^+^ T cells. The dashed line marks the inflection point above which enhancers are classified as super-enhancers (SE). The *SESN3* super-enhancer is labelled in red and is ranked as the 75^th^ strongest super-enhancer out of 760 super-enhancers and 15,281 total enhancers. **d**, RPKM normalised ATAC signal at a genomic window corresponding to the defined super enhancer in a variety of blood cell types with both unstimulated (blue) and stimulated (green) paired data. Source data

Using this approach, we generated six separate profiles from a single experiment. This included separation of the reference and alternate alleles for the three possible editing outcomes: editing with either the control or the CTCF motif targeting gRNA and unaltered alleles (Fig. 5c,d). For both the reference and alternate alleles, no differences were seen between the unedited alleles (wild-type) and the alleles with control edits. On the alternate chromosome, when the CTCF motif edits had occurred, the strong contacts to the convergent downstream CTCF sites were reduced to the levels seen on the wild-type reference allele (Fig. 5e and Extended Data Fig. 9a). Editing with the CTCF motif targeting gRNA on the alternate allele also reverted the nanoscale chromatin structure to the inert nucleosome pattern seen with the reference allele (Fig. 5f). Collectively, these results highlight how MCCv can be paired with precise genome editing in primary human cells, to definitively reveal the impact of engineered variants on chromatin architecture.

### Neo-CTCF motif blocks super-enhancer–promoter contacts

**Fig. 6:**
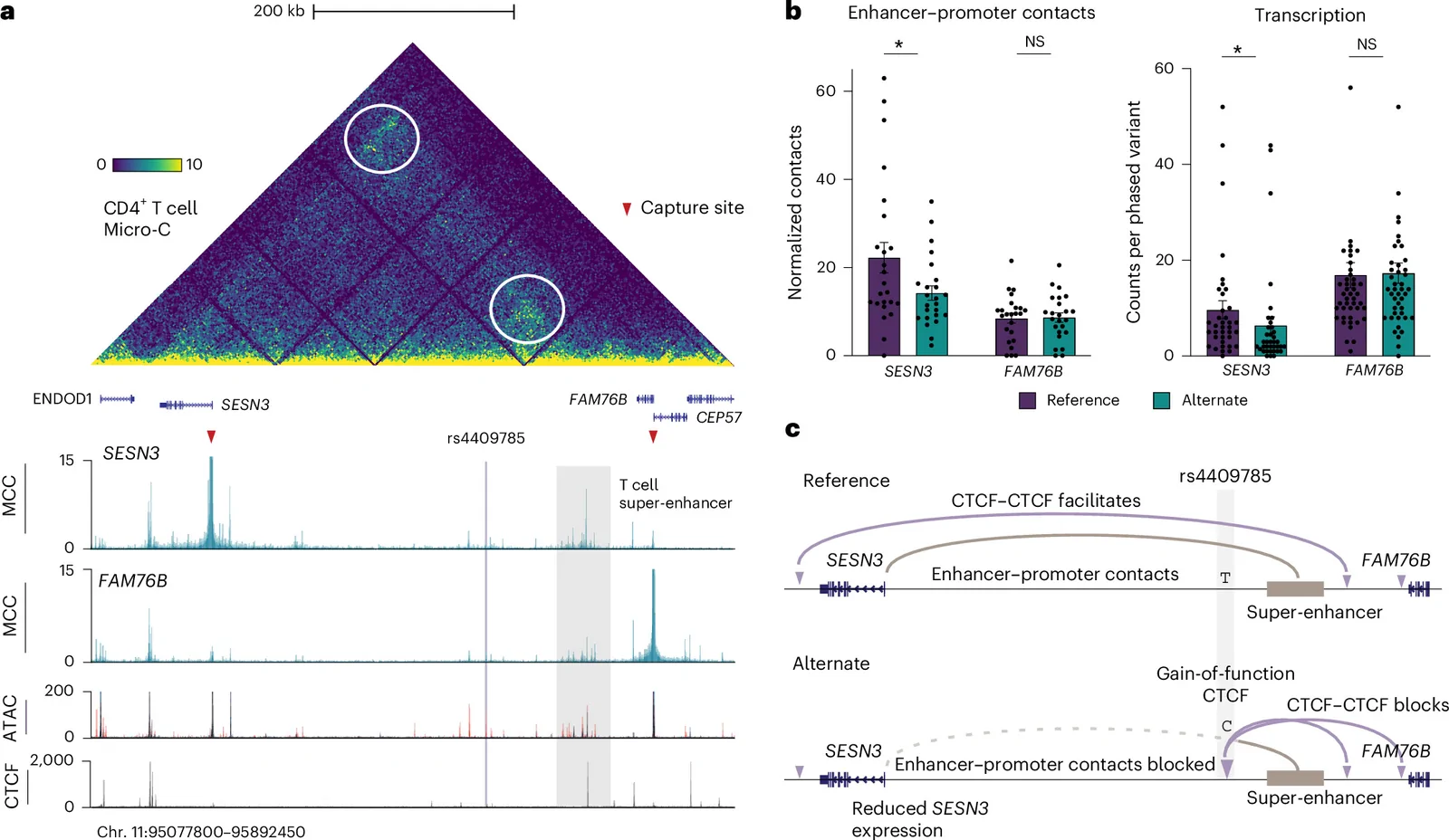
Neo-CTCF site blocks super-enhancer–promoter interactions, reducing *SESN3* expression. **a**, A 2.5-kb, binned Micro-C, ATAC–seq (CD4^+^, unstimulated blue, stimulated red, overlay) and CTCF ChIP–seq for the 11q21 locus. Bulk MCCs from the promoters of *SESN3* and *FAM76B* are shown in blue. The location of the super-enhancer is highlighted in gray. **b**, Phased enhancer–promoter contacts (*SESN3* (*q* = 0.018, *n* = 24 peaks across six independent MCC libraries) and *FAM76B* (*q* = 0.916, *n* = 24 peaks across six independent MCC libraries), Student’s *t*-test, two-stage step-up procedure for multiple-test correction, mean ± s.e.m.) and intron-rich RNA (*SESN3* (*q* = 0.00054, *n* = 37 phased heterozygous variants across two donors) and *FAM76B* (*q* = 0.11, *n* = 45 phased heterozygous variants across two donors), Wilcoxon’s signed-rank test, two-stage step-up procedure for multiple-test correction, mean ± s.e.m.) from MCCv-phased heterozygous variants for both *SESN3* and *FAM76B*. **c**, Working mechanistic model for the 11q21 risk locus. The neo-CTCF site competes with canonical upstream boundary elements for convergent downstream CTCF sites, forming a subdomain that isolates the super-enhancer away from the promoter of *SESN3*. Source data

Neither allele of rs4409785 made significant contacts with any gene promoters, including the nearest genes *FAM76B* and *SESN3*. However, a cluster of enhancers was identified between these two genes, which meets the criteria for super-enhancer activity^35^, specifically in activated effector CD4^+^ T cells (Extended Data Fig. 9b,c). MCC data from the promoters of the *SESN3* and *FAM76B* genes showed that these genes both contact the super-enhancer (Fig. 6a).

We hypothesized that the creation of the CTCF motif on the alternate allele would reduce transcription of *SESN3* by blocking the super-enhancer from contacting the *SESN3* promoter. We utilized MCCv data from this locus to phase rs4409785 with heterozygous variants in the promoter regions of *SESN3* and *FAM76B* and multiple intronic variants in both genes (Extended Data Fig. 9d). We used these variants to perform MCCv from the *SESN3* promoter relative to the genotype of rs4409785, demonstrating that the neo-CTCF created by the disease allele reduces contacts between the T cell-specific super-enhancer and the *SESN3* promoter (Fig. 6b). Combining MCCv with RNA-seq analysis linked rs4409785 with a 36.6% reduction of *SESN3* transcript expression on the risk allele, consistent with the reduction in super-enhancer–promoter contacts (Fig. 6b). Taken together, we have demonstrated that rs4409785 functions through a CTCF-mediated, super-enhancer-blocking mechanism to reduce *SESN3* expression (Fig. 6c).

### In vivo validation of *SESN3* and identification of its role in the mTOR pathway through tryptophan sensing

**Fig. 7:**
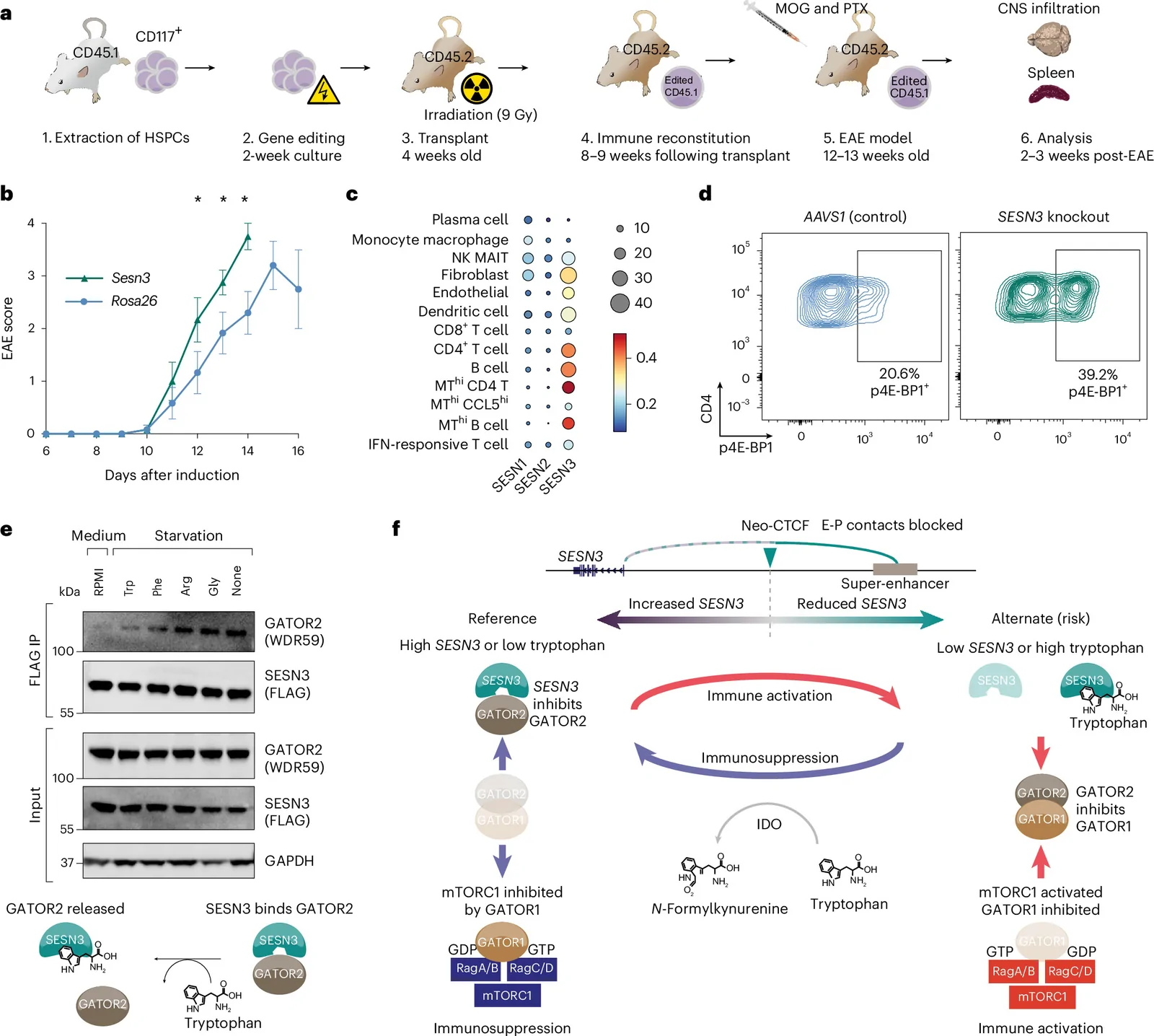
Functional characterization of SESN3. **a**, Experimental design of the mouse EAE model. **b**, Clinical EAE score for both *Rosa26* and *Sesn3* cohorts. For each day where both cohorts were represented, scores were compared statistically (*n* = 6 mice per condition, day 12, *q* = 0.0024; day 13, *q* = 0.0089; day 14, *q* = 0.0024; two-sided, unpaired, Student’s *t*-test with single pooled variance, mean ± s.e.m.). **c**, Dot plot representation of *SESN* gene family members’ scRNA-seq expression from synovial tissue of patients wth RA^56^. **d**, Representative flow cytometry contour plots showing a positive population of CD4^+^ (*y* axis) and phospho-4E-BP1^+^ (mTORC1 signaling marker, *x* axis). **e**, Western blots for FLAG-tagged SESN3 or GATOR2 complex subunit, WDR59, either from cell lysate (input) or from co-immunoprecipitation using anti-FLAG antibody-conjugated beads. Experimental conditions compared were a medium control (fed state) versus a starved state in Hanks’ balanced salt solution (HBSS) with or without various free amino acids. Specific indicated free amino acids were added to the starved state during the 1-h starvation at 500 µM. **f**, Schematic for working model of the effect of rs4409785 on reducing *SESN3* gene expression and, in turn, how this may affect mTORC1 signaling in starvation conditions, specifically low tryptophan availability. *AAVS1*, adeno-associated virus integration site 1; CNS, central nervous system; E-P, enhancer–promoter; GAPDH, glyceraldehyde 3-phosphate dehydrogenase; IFN, interferon; IP, immunoprecipitation; MOG, myelin oligodendrocyte glycoprotein; MT^hi^, mitochondrial high; mTORC1, mTOR complex 1; PTX, pertussis toxin. Source data

**Extended Data Fig. 10:**
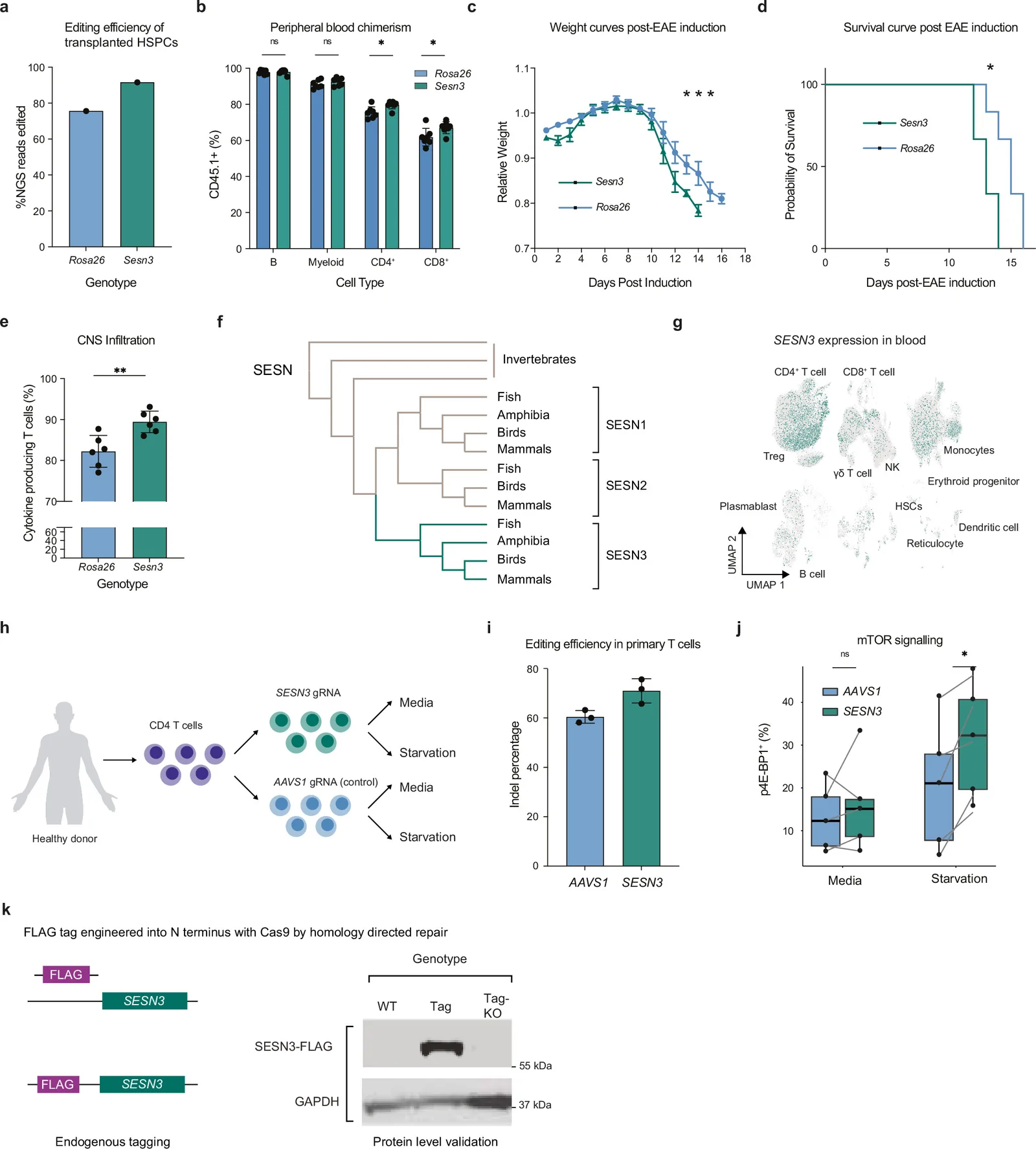
Further characterisation of the *SESN3* gene. **a**, % edited reads, 24 h post-nucleofection in mHSPCs for *Rosa26* and *Sesn3* guides (n = 1). **b**, CD45.1^+^ % in different cell types; CD8^+^ (q = 0.0029), CD4^+^ (q = 0.0098), Myeloid (q = 0.344), B cells (q = 0.723) in *Rosa26* or *Sesn3* conditions (n = 7 mice per condition, Student’s t-test single pooled variance, two-sided, unpaired, two-stage step-up procedure for multiple test correction, mean ± s.d.). **c**, Relative mouse weights for *Rosa26* and *Sesn3* cohorts (day 12, q = 0.023; day 13, q = 0.026; day 14, q = 0.026) (n = 6 mice per condition, Student’s t-test single pooled variance, two-sided, unpaired, two-stage step-up procedure for multiple test correction, mean ± s.e.m.). **d**, Survival curve post EAE-induction for *Rosa26* and *Sesn3* conditions (*P* = 0.019, n = 6, Grehan-Brislow-Wilcoxon test, two-sided). **e**, % infiltrating activated T cells (CD4^+^ IFNγ^+^ IL17^+^ CD44^high^) in CNS for *Rosa26* and *Sesn3* cohorts (*P* = 0.0038, Student’s t-test, two-sided, n = 6 mice per condition, mean ± s.d.). **f**, Rooted phylogenetic maximum-likelihood tree of *SESN* gene family from amino-acid sequence. **g**, Single-cell RNA-seq UMAP from healthy volunteer PBMCs (*SESN3* expression highlighted in green). **h**, Experimental scheme for ex vivo human T cell editing in fed and starved conditions. **i**, Tracking of indels by decomposition analysis 72 h post-nucleofection of T cells for *AAVS1* and *SESN3* sgRNAs (n = 3 nucleofections, mean ± s.d.). **j**, Phospho-4E-BP^+^ CD4^+^ T cells % for *AAVS1* or *SESN3* sgRNA nucleofections in media (*P* = 0.462) or starvation (*P* = 0.014) (n = 5 nucleofections across donors, Student’s t-test, two-sided, median, interquartile range (box; 25th–75th percentiles), whiskers extending to the most extreme data points within 1.5 × the interquartile range*)*. **k**, Immunoblot for anti-FLAG-SESN3 in wildtype (WT), FLAG knock-in (Tag), and FLAG knock-in edited with sgRNAs targeting *SESN3* (Tag-KO). Source data

Among the pleiotropic autoimmune associations, rs4409785 was significantly associated with an increased risk of MS. To study the contribution of *SESN3* to autoimmunity in vivo in both lymphoid and myeloid compartments, we combined transplantation of genome-edited hematopoietic stem and progenitor cells (HSPCs) with the MS-like mouse model experimental autoimmune encephalomyelitis (EAE) (Fig. 7a)^36^. Using Cas9 ribonucleoprotein we achieved high editing efficiencies of *Sesn3* or the control *Rosa26* locus (Extended Data Fig. 10a)^37^. After transplantation, there were high levels of immune reconstitution of transplanted cells and there was a small but significant difference in the percentage of both CD4^+^ and CD8^+^ populations in *Sesn3* knockout cells compared to *Rosa26* controls (Extended Data Fig. 10b). Then 10 d post-EAE induction, we observed an accelerated onset of EAE symptoms and weight loss in mice transplanted with *Sesn3* knockout HSPCs compared to *Rosa26* controls (Fig. 7b and Extended Data Fig. 10c,d). Flow cytometry on the central nervous system of the mice showed that *Sesn3* editing resulted in an increase in infiltrating activated T cells (Extended Data Fig. 10e).

SESN1 and SESN2 are known to play a role in inhibiting mammalian target of rapamycin (mTOR) signaling but the role of SESN3 has not been well characterized. SESN3 is highly conserved across vertebrates and is the most evolutionarily distinct member of the Sestrin family (Extended Data Fig. 10f). It is highly expressed in a variety of immune cell subpopulations compared to *SESN1* and *SESN2* in both healthy donors and patients with autoimmune disease (Fig. 7c and Extended Data Fig. 10g)^38,39^.

To gain further mechanistic understanding of the role of *SESN3* in autoimmunity, we performed editing experiments in primary human CD4^+^ T cells (Extended Data Fig. 10h,i). Under normal conditions, *SESN3* knockout did not significantly alter mTOR signaling. However, under starvation conditions, which included removal of all amino acids, *SESN3* knockout in primary human CD4^+^ T cells had increased mTOR signaling activity (Fig. 7d and Extended Data Fig. 10j). This demonstrates that reduced levels of *SESN3* in starvation conditions lead to increased mTOR signaling, which is known to increase immune activation, consistent with the direction of effect seen with the disease-associated haplotype.

SESN2 is known to sense leucine, which abrogates its inhibition of the key mTOR regulator GATOR2. However, the mechanism by which SESN3 mediates its effects is not well understood. To delineate the biochemical mechanism by which SESN3 alters mTOR signaling, we generated an endogenously tagged SESN3 Jurkat cell line (Extended Data Fig. 10k). Using co-immunoprecipitation, we observed an increased protein–protein interaction between SESN3 and GATOR2 in the starvation conditions previously introduced (Fig. 7e). We went on to show that this protein–protein interaction was reduced by tryptophan but not other free amino acids (Fig. 7e). These data show that *SESN3* expression is important for normal T cell homeostasis, through inhibition of mTOR signaling during periods of low tryptophan availability (Fig. 7f).

## Discussion

In this study, we describe a high-resolution method of delineating chromatin architecture changes due to noncoding variants using in vivo nanoscale DNA structure within individual *cis*-regulatory elements. Simultaneously, MCCv also maps enhancer–promoter contacts precisely between alleles in the same population of cells. In addition, it can be used to phase variants across the locus of interest, allowing other data types such as RNA-seq to be integrated. MCCv can also be combined with precise genome editing in primary human cells, providing a robust approach to demonstrate how mutations affect chromatin structure.

Although there have been previous studies demonstrating allele-specific 3C analysis^19,40,41^ and quantitative trait mapping of 3C contacts^42^, these have used restriction-enzyme-based methods, which have limited utility for interrogating variants. This is because data are generated only from restriction-enzyme cut-sites and only ~30% of variants lie close enough to these sequences to be studied. The MNase-based approach used by MCCv circumvents this issue because data can be generated from any site of interest and data with much greater contact frequency resolution are generated than is possible using previous approaches^24^. MCCv also allows the nanoscale architecture of *cis*-regulatory elements to be compared between alleles. Images of all single-allele nanoscale maps and their corresponding genomic coordinates have been made available to view in a publicly available repository^43^.

MCCv allows up to 12 independent replicates to be simultaneously analyzed with capture enrichment. In this work we captured from 405 sites but it is likely that this could be extended to >1,000 targets in a single experiment (Supplementary Note 2)^20^. The parallelized sequencing library preparation protocol means that each MCCv library can potentially be used in ten or more capture reactions with different targets.

We have validated the platform by identifying causal genes from GWAS signals associated with IMIDs. We prioritized likely CD4^+^ T cell enhancer and CTCF-altering variants and performed deep targeted capture. Precise mapping of variant–promoter contacts allowed us to nominate 251 causal genes. Contact between an enhancer and promoter does not always lead to alterations in transcription^44^. However, the absence of contact strongly argues against direct regulation and the MCCv platform, therefore greatly reduces the number of potential causal genes affected by disease-associated noncoding variants. We demonstrated that variants that result in detectable changes in allelic contact frequency are significantly enriched (approximately threefold) for contact to nominated genes with an allelic imbalance in transcription. We envisioned that the number of detectable gene expression changes would increase, with more sensitive methods of detecting intron-rich RNA^45^. Given this enrichment score, we believed that, after their identification, variants require formal validation at the level of either the variant or the gene or, ideally, both.

The utility of the MCCv approach was demonstrated by the identification of a variant that increases the risk of multiple autoimmune diseases through a gain-of-function, CTCF motif-based mechanism. We generated six profiles of editing outcomes from the reference and alternate alleles of this variant in a single experiment, demonstrating that the CTCF-binding site in the alternate allele alters chromatin structure. It is interesting that this variant does not contact gene promoters and we went on to demonstrate that the variant causes an allele-specific reduction in contacts between *SESN3* and a T cell-specific super-enhancer, which may result in a decrease of *SESN3* transcription seen linked to that haplotype. Although it has previously been reported that CTCF can block enhancer–promoter contacts^46^, and that common variants may affect its binding^47^, our findings demonstrate a GWAS variant functioning in a gain-of-function manner with respect to the CTCF motif. The molecular mechanism underpinning this signal would likely only be fully elucidated with high-resolution, allele-specific 3C methods such as the one presented here.

Our study also identifies *SESN3* as an underappreciated contributor to autoimmune disease. The other homologs (*SESN1* and *SESN2*) have been identified as regulators of the mTOR pathway, which is known to play a critical role in modulating immune cell function^48^. We found that *SESN3* is expressed at greater levels in immune cells than the other homologs. SESN2 has been shown to sense amino acids, in particular leucine^49,50^, and it has been suggested that SESN3 may sense aromatic amino acids^51,52^. Here we have demonstrated that SESN3 senses free tryptophan levels in a T cell context. Recently there has been great interest in the role of tryptophan in immune cell function, particularly in relation to the indoleamine-2,3-dioxygenase pathway^53^. It is known that reduced indoleamine-2,3-dioxygenase function leads to immune activation through mTOR^54^ and we have shown that SESN3 is likely to be important in mediating this process through its tryptophan-dependent interaction with the GATOR2 complex. To further validate the role of *SESN3* in autoimmune disease, we used the EAE mouse model of MS. Using stem-cell transplantation with *Sesn3* knockout in HSPCs, we observed accelerated disease progression in the mice, confirming the role of *SESN3* in autoimmune disease in vivo.

The current cost of drug development is largely driven by the failure of clinical programs^55^. The probability of progressing through clinical trials is increased approximately 2.6-fold by genetic evidence^56,57^. Although there is a huge wealth of genetic variants linked to human disease, it has been challenging to convert these data to drug targets due to the intricate mechanisms by which these variants control gene expression^10,58,59^. Here we have presented MCCv, which is a multifaceted platform that allows identification of causal variants and associated genes at scale.

## Methods

### Cell culture

Jurkat cells, clone E6-1 (American Type Culture Collection (ATCC), cat. no. TIB-152), were cultured in ATCC-modified Roswell Park Memorial Institute medium (Gibco, cat. no. A1049101) with 10% fetal bovine serum (FBS; Sigma-Aldrich) at 37 °C and 5% CO_2_.

### Isolation and culture of human T cells

Peripheral blood was obtained with ethical approval by venesection from volunteers without self-reported autoimmune or other chronic immunological or inflammatory disorders who were recruited through the Investigation of hematopoiesis in the Healthy Volunteers Study (no. CUREC1 R88300/RE001). Written informed consent was obtained from all participants. Blood, 100 ml, was mixed 1:1 with phosphate-buffered saline (PBS) before layering 30 ml on 15 ml of Lymphoprep (STEMCELL Technologies, cat. no. 18061). This was spun at room temperature for 30 min at 800*g* with minimal break and acceleration. The peripheral blood mononuclear cell layer was isolated and CD4^+^ or CD3^+^ pan-T cells were enriched with CD4^+^ magnetic beads (Miltenyi, cat. no. 130-045-101) or pan-T cell isolation kit (Miltenyi, cat. no. 130-096-535), respectively, as per the manufacturer’s instructions. CD4^+^ or CD3^+^ cells were cultured at 1 × 10^6^ cells ml^−1^ in Immunocult-XF T cell expansion medium (STEMCELL Technologies, cat. no. 10981) supplemented with 25 μl ml^−1^ of Immunocult Human CD3, CD28, CD2 T cell activator (STEMCELL Technologies, cat. no. 100-0785) and 10 ng ml^−1^ of human interleukin-2 (Invitrogen), for 60 h, before collection.

### Manufacture of the 3C library

Aliquots of 1 × 10^7^ cells were fixed in 37% (v:v) formaldehyde (Sigma-Aldrich, cat. no. 47608, 250 ml) to a final concentration of 2% at room temperature with rotation. After 10 min, the reaction was quenched with ice-cold 1 M glycine (final concentration 130 mM) before being centrifuged for 10 min at 300*g*. Cells were washed with ice-cold PBS before reconstitution in 1 ml of PBS. Cells were incubated for 10 min at room temperature with digitonin (Sigma-Aldrich, cat. no. D-141; 0.005% w:v final concentration). Cells were snap-frozen and stored at −80 °C. An aliquot of frozen permeabilized cells was thawed at room temperature before spinning for 5 min at 300*g*. Cells were resuspended in a reduced calcium MNase buffer (10 mM Tris-HCl, pH 7.5, and 1 mM CaCl_2_), before being split into three reactions for MNase titration (NEB, cat. no. M0247), which typically ranged from 5 Kunitz units per reaction to 20 Kunitz units per reaction. The reaction was incubated at 37 °C for 1 h in an Eppendorf Thermomixer at 500 r.p.m. with shaking. Quenching was achieved by adding ethylene glycol-bis(2-aminoethylether)-*N*,*N*,*N*ʹ,*N*ʹ-tetra-acetic acid (EGTA; Sigma-Aldrich, cat. no. E3889) to a final concentration of 5 mM. Of the reaction, 20% was taken to assess digestion efficiency. The remaining reaction was centrifuged, PBS washed and resuspended in DNA ligation buffer (Thermo Fisher Scientific; final concentrations: 40 mM Tris-HCl, pH 7.5, 10 mM MgCl_2_, 10 mM dithiothreitol and 5 mM ATP) supplemented with dNTPs (final concentration 400 µM each for dATP, dCTP, dGTP and dTTP (Thermo Fisher Scientific, cat. no. R0191)) and EGTA 5 mM. T4 polynucleotide kinase PNK (NEB, cat. no. M0201L) and DNA polymerase I large (Klenow) fragment (NEB, cat. no. M0210L) were at final concentrations of 200 U ml^−1^ and 100 U ml^−1^, respectively, T4 DNA ligase (Thermo Fisher Scientific, high-concentration ligase (30 U µl^−1^), cat. no. EL0013) to a final concentration of 300 U ml^−1^ was added and the reaction was incubated at 37 °C for 2 h before cooling to 20 °C and overnight incubation on an Eppendorf Thermomixer with intermittent 500-r.p.m. shaking (30 s on and 30 s off). Chromatin was de-crosslinked (proteinase K, 65 °C, >2 h) and DNA was isolated using the QIAGEN DNeasy blood and tissue kit (cat. no. 69506). Digestion and ligation efficiencies were assessed on a D1000 Agilent TapeStation (Agilent, cat. no. 5067-5583).

### Preparation of 3C sequencing library

For chosen 3C libraries 2–6 µg was sonicated to an average fragment size of 200 bp (Covaris S220 Focused Ultrasonicator, 3 cycles of 60 s, duty cycle 10%, intensity 5, 200 cycles per burst) before 1.8X Ampure XP bead (Beckman Coulter, cat. no. A63881) purification (100-µl elution). This was split into two library preparation reactions to maximize library complexity. The NEB Ultra II (NEB, cat. no. 7645S) protocol was followed with the following modifications: 5 µl of adapter was ligated, 1.8X Ampure XP bead clean-ups and the final PCR was performed in duplicate using Herculase II (Agilent).

### Capture oligonucleotide design

Oligonucleotides of 120 bp were designed using CapSequm^20^, ensuring that the variant of interest resides in the central position. If this was not possible due to spurious alignment (BLAT score >30), repeats or high GC content (>80%), then oligonucleotides would be iteratively designed with the variant in all possible positions. If none of these designs passed filtering, oligonucleotides were designed to the summit of the open chromatin peak defining the regulatory element. xGen MRD Hyb Panels from IDT or single 5ʹ-biotinylated oligonucleotides were ordered depending on the size of the design. All oligonucleotide sequences are listed in Supplementary Table 2.

### Oligonucleotide hybridization, capture and sequencing

Then, 1–2 µg of 6–12 libraries were pooled to make a 12-µg total capture reaction. Hybridizations used the Roche HyperCapture Target Enrichment Kit reagents (Roche, cat. no. 9075828001) following a modified protocol^23^. The hybridization reaction was denatured at 95 °C (10 min) before transferring to 47 °C. Biotinylated oligonucleotides, at 29–290 µM (depending on target number), were added and incubated for 48–72 h. To purify captured DNA, Streptavidin MyOne C1 beads (Thermo Fisher Scientific, cat. no. 65001) were added, followed by washing and amplification using the Roche HyperCapture procedure. A second round of hybridization capture was performed with the same reagents scaled down to 1× volumes and a 24-h incubation. The 150-bp paired-end Illumina sequencing was performed to a read depth of 0.1 × 10^6^ to 0.5 × 10^6^ reads per target per library (Supplementary Note 2).

### GWAS variant prioritization

IMID GWAS was sourced from the GWAS catalog^6^. GWAS signals within the extended major histocompatibility complex cluster (hg38 coordinates: chr. 6: 28510020–33480577) were removed and signals merged if lead SNPs were within linkage (*r*^2^ > 0.8) using the National Institutes of Health (NIH) linkage disequilibrium (LD) matrix^60^. Lead SNPs were imputed with LD link^61^ (*r*^2^ > 0.7) using matched ancestry to the original GWAS. All variants within each haplotype were annotated using the SNP_prioritization_pipeline:

1. Coding: nonsynonymous variants labeled with ANNOVAR^62^ run with default parameters on hg38 genome build.
2. Splicing: variants with spliceAI *δ* scores ≥0.2 (donor or acceptor).
3. 3’-UTR: ANNOVAR-defined 3’-UTR variants intersecting known micro-RNA-binding sites^63^.
4. Promoter or enhancer: variants intersecting cell type-specific ATAC^64^. ATAC peaks were called using LanceOtron and filtered (peak score ≥0.5). Intersections passed if the target SNPs was within 100 bp of the peak summit and the signal coverage at the variant position was greater than the average signal coverage of the peak. Promoter variants were intersected with cap analysis of gene expression sequencing-defined transcription start sites^65^. Distal ATAC–seq peaks were classified as putative enhancers.
5. CTCF: intersection (as above) with deeply sequenced CTCF ChIP–seq data from 50 healthy donors. Peaks were called using LanceOtron and filtered (peak score >0.5).

Oligonucleotide probes were designed to all enhancer and a subset of CTCF variants, and captured in activated CD4^+^ T cells from three donors. This included haplotypes for which there was a secondary molecular mechanism annotated, beyond the potential enhancer mechanism. A variety of control variants was included: cell-type controls (SNPs associated with disease but intersecting monocyte rather than CD4^+^ T cell open chromatin), haplotype controls (SNPs in a shared haplotype with a captured enhancer or CTCF SNP, but itself annotated as neutral) and enhancer controls (SNPs intersecting CD4^+^ T cell open chromatin, but not associated with IMIDs).

### RNA-seq

RNA was extracted from 1 × 10^6^ cells with the Zymo Direct-zol kit (cat. no. R2051). Ribosomal RNA was depleted using the RiboMinus Eukaryote System v2 (cat. no. A15020). Poly(A^+^) RNA was isolated with the NEBNext poly(A) mRNA magnetic isolation module (cat. no. E7490), while keeping the flow-through poly(A^−^) RNA. Both were converted to complementary DNA and library prepped using the NEBNext Ultra II Directional RNA Library Prep Kit for Illumina (cat. no. E7760). Libraries were sequenced by paired-end sequencing with a 75-cycle, high-output NextSeq 500 kit (Illumina).

### Allele-specific RNA-seq analysis

Skew analysis was performed with customized scripts utilizing Bcftools mpileup (Wellcome Sanger Institute). Only genes with ≥25 phased variants were considered. To ensure independence of phased SNPs, we compared the average alternate ratio of pairs of phased SNPs within the same gene across distances, within and between donors. The distance at which skew behavior was comparable was chosen (Supplementary Fig. 5). Therefore, variants separated by <250 bp (median distance between gene body SNPs = 950 bp, the mean 90th percentile RNA insert size across analyzed BAM files = 249 bp) were collapsed (read counts averaged). Haplotype significance of the gene was tested using a Wilcoxon’s signed-rank test of the phased variants within a donor. When multiple donors were heterozygous for the MCC variant, *P* values were combined with Fisher’s method if the direction of the effect was concordant. Haplotype phase in relation to the MCC variant was assigned using MCCv phasing.

### Single-allele MCC analysis

Adapter sequences were removed using Trim Galore (Babraham Institute, v0.3.1), before full read reconstruction using FLASH (v1.2.11). Reads were then aligned using the nonstringent aligner BLAT (v35) to an 800-bp mini-chromosome around the capture site. MCCsplitter.pl divided reads into sub-FASTQs for every capture site (SNP). These were then aligned with Bowtie2 to the hg38 genome. Sequence Alignment Map (SAM) files were parsed into MCC_SNP_splitter.pl, dividing the SAM file into corresponding reference and alternate genotypes by querying reads at specified positions. To avoid mapping bias, MCCv sites were aligned using BLAT and Bowtie2 to a customized alternate genome. The reference SAM files were taken from the reference genome-aligned split SAM file and vice versa for the alternate aligned SAM file. Split SAM files were then parsed into MCCanalyser.pl, which stringently removes PCR duplicates and identifies the precise position of ligation junctions between subreads as well as orientation of the reads relative to the junction. Junction tracks were normalized by the total number of *cis*-unique ligation junctions per individual allele. Allele-specific signal pileups and footprinting tracks were visualized on the University of California, Santa Cruz (UCSC) genome browser. MCCv heatmaps were generated with a customized script which plotted the ultra-short-range capture site junctions as a 2D matrix in an allele-specific manner at single-basepair resolution. Directional windowing was achieved as previously described^30^. MCCv phasing was achieved by variant calling unique ligation junctions at specified regions of heterozygosity within the locus, using Bcftools mpileup on the allele-split SAM files.

### Statistical analysis of MCCv nanoscale matrices

MCCv nanoscale matrices were compared with basepairC (Supplementary Methods). Allele-specific matrices were symmetrized, combined across samples and normalized using the trimmed mean of M-value method^66^. Allelic effects were assessed using quasi-Poisson, generalized linear models with junction counts as the outcome and allele as the covariate. To estimate effect sizes expected from random sample bias, ten sham allele assignments matching the experimental design were modeled to generate an empirical null distribution of coefficients and their standard errors.

### MCCv peak calling

The average signal of a region in MCC is highly dependent on the distance to the capture point, making it challenging to find regions of enrichment (peaks). Three parameters define enrichment: the average signal, the background against which the signal is compared and a threshold that the enrichment level must reach. As these parameters will vary across the region of interest within an MCC track, peak calling is performed using sets of values, rather than individual values, for each of these three parameter types. This results in 27 peak calls for each track. Each peak call is mapped back to the reference genome. Every base around the capture points (2 Mb either side) included as part of a peak in at least ten thresholds was classified in a final peak. This threshold allows for stringent filtering while retaining the sensitivity to detect peaks in distal regions with lower coverage. Additional filtering is done such that peaks must have between 50 and 500 contiguously enriched bases, as well as a minimum depth of 5 reads. The parameter set for the average signal was window size (rolling mean coverage) (200 bp, 400 bp and 600 bp), for background it was window size (10^4^ bp, 10^5^ bp and 10^6^ bp) and for threshold enrichment it was a fold increase in signal against background (twofold, fourfold and sixfold).

Peaks were assigned as promoter peaks based on open chromatin and transcription start site intersection. CTCF peaks are assigned based on open chromatin and CTCF ChIP–seq intersection. Remaining open, chromatin intersections are assigned as enhancers. Intersections were filtered as previously described. Junction counts were quantified within the range of the called peaks for both alleles and normalized by the total number of PCR-deduplicated unique ligations per allele. This was repeated for all independent replicates of MCC data and counts were compared statistically using a paired, two-sided Student’s *t*-test followed by FDR correction using fdrtools^67^. Further details can be found in Supplementary Methods.

### Estimating background allelic expression for MCCv enrichment

To investigate whether MCCv changes predict variants will be in contact with genes displaying allelically imbalanced expression, we calculated the percentage of genes with allelic imbalance genome wide in three donors. We utilized phased whole-genome sequencing for haplotype reconstruction and applied the strict phasing criteria introduced previously. For a secondary estimation of the background, we took the percentage of genes with allelic imbalance linked to heterozygous variants with no 3C skew. Backgrounds were compared to variant–gene pairs with evidence of MCCv change at the variant. We divided variant–gene pairs into whether the linked gene was the primary or the secondary nominated gene (Supplementary Note 1).

### Nomination of causal genes

MCC peaks were assigned as promoters by LotronMCC as previously introduced. If a given SNP had multiple promoter peaks, they were ranked based on the number of normalized ligation junction counts. The promoter with the highest counts in each variant MCCv map was assigned as the primary nominated gene for the given SNP.

### Base editing of primary T cells

CD4^+^ T cells, 1 × 10^6^, were activated in culture for 60 h before resuspension in 20 µl of P3 solution (Lonza, cat. no. V4XP-3032). Next, 2 µg of BE4max mRNA and 100 pmol of synthetic guide (sg)RNA (Supplementary Methods) were added before nucleofection with program no. EO-115. After 24 h, nucleofected cells were split into five separate 1-ml wells of Immunocult-XF T cell expansion medium to wash out the P3 solution. After 4 d, gDNA was extracted using the QIAGEN DNeasy kit. Using primers spanning the control and CTCF edit sites (Supplementary Table 3), gDNA was amplified with high-fidelity Q5. Of this PCR reaction, 1 µl was used in a second PCR to add dual-index sequencing adapters (NEBNext Multiplex Oligos for Illumina kit, NEB Ultra II master mix). The products were sequenced with Illumina MiSeq platform and analyzed with CRISPResso^68^.

### Mouse transplantation and EAE

Mice breeding or maintenance and EAE inductions were performed under project license no. PP8589020 approved by the UK Home Office under the Animal (Scientific Procedures) Act 1986, in accordance with the 3Rs principles (replacement, reduction and refinement) in animal research. Mice were maintained in individually ventilated cages containing environmental enrichment, including red tunnels or houses and balconies. Animals were kept under a 12-h light-to-dark cycle at 21–22 °C, with a relative humidity of 45–65%.

The 3-week-old to 4-week-old C57/BL6 mice (Charles River) were irradiated (2 doses of 5.5 Gy with a 4 h interval) using a γ irradiator. Mice were then injected intravenously with 500,000 edited mouse CD117^+^ HSPCs. At 8 weeks post-transplantation, blood was withdrawn from the tail vein to check for hematopoietic reconstitution by flow cytometry (Supplementary Table 4). By 13–14 weeks of age, mice were immunized with a pre-made MOG35-55/CFA Emulsion kit (Hooke Lab, cat. no. EK-2110) according to the manufacturer’s instructions. Clinical EAE scores were determined daily, ranging from score 0 (no overt disease) to 4 (complete hindlimb paralysis). If mice reached a score of 4 for 4 d consecutively or lost 20% of their body weight, they would be euthanized by perfusion under terminal anesthesia (a schedule 1-approved method) according to an approved project license (no. PP8589020). Animals that were killed for animal welfare reasons related to EAE severity were excluded from the analysis after the timepoint of their death.

### Mouse cell isolation

Spleen and inguinal lymph nodes were obtained from euthanized mice by standard methods and run through a 70-µm cell strainer. Red blood cell lysis buffer (BioLegend) was used to remove red blood cells. Brain and spinal cords were dissected and digested in HBSS supplemented with 0.05% collagenase D (Roche), 0.1 mg ml^−1^ of tosyl-L-lysyl-chloromethane hydrochloride (TLCK; Sigma-Aldrich) and 0.025 U ml^−1^ of deoxyribonuclease I (Thermo Fisher Scientific). A Percoll (GE Healthcare Life Sciences) gradient centrifugation at 200*g* for 40 min was used to isolate mononuclear cells. Cells were washed twice with PBS with 2% FBS before staining with a mononuclear antibody panel (Supplementary Figs. 6 and 7 and Supplementary Table 4).

### Starvation experiments in primary T cells

After 60 h of activation, pan-T cells were resuspended in 20 µl of P3 solution (Lonza, cat. no. V4XP-3032) to which 100 pmol of Synthego sgRNAs (targeting control *AAVS1* or *SESN3*) were added along with 100 pmol of ALT-R Cas9 (IDT, cat. no. 1081058). This mixture was nucleofected with program EO-115. After 4 d, cells were counted and replated at a ratio of 2 × 10^6^ cells per 1 ml of medium or HBSS for 4 h. Cells were washed (PBS with 2% FBS) before 15 min of surface staining in the dark at room temperature (Supplementary Table 4). Cells were fixed, permeabilized and intracellularly stained using the True-Nuclear Transcription Factor buffer set (BioLegend, cat. no. 424401). Then, 200 µl of TrueNuclearFix 1× was applied for 45 min in the dark at room temperature before two 200-µl washes of True-Nuclear 1× Perm Buffer. Intracellular stain was performed in True-Nuclear 1× Perm Buffer for 30 min at room temperature in the dark. Cells were washed and resuspended in 200 µl of PBS (2% FBS) before acquisition on the LSR Fortessa X50 (Supplementary Fig. 8).

### Co-immunoprecipitation and western blotting

*SESN3* flag-tagged cells (Supplementary Methods) were cultured in Roswell Park Memorial Institute medium (fed) or HBSS (starved) (with or without the indicated amino acid at 500 µM) for 1 h before lysing for 30 min on ice with a buffer containing 25 mM Tris-HCl, pH 7.5, 150 mM NaCl, 1 mM EDTA, 0.5% NP-40, 5% glycerol and 1× protease inhibitor cocktail (Merck, cat. no. 539131-1VL). Lysates were quantified by bicinchoninic acid assay before immunoprecipitation with anti-FLAG M2 magnetic beads (cat. no. M8823) for 2 h at 4 °C. Immunoprecipitates were washed 3× with lysis buffer before elution with 2× FLAG peptide (cat. no. F4799). Co-immunoprecipitations were visualized by running samples on a NuPage Bis-Tris 4–12% gel. After transfer and incubation with primary antibodies and horseradish peroxidase-conjugated secondary antibodies (Supplementary Table 4), the membranes were incubated with ECL (Thermo Fisher Scientific) before chemiluminescence was recorded on the ChemiDoc Touch imager (BioRad). Densitometric analysis was performed using Fiji. A representative western blot from two independent experiments with similar results was chosen for figure presentation.

### Statistics and reproducibility

Statistical tests were performed using GraphPad Prism and the SciPy statistical Python package. Data were examined for normal distributions using Shapiro–Wilk tests. For normally distributed data, paired or independent, two-sided Student’s *t*-tests were performed depending on the nature of the comparison. Otherwise, statistical comparisons were made with Wilcoxon’s signed-rank tests (paired) or Mann–Whitney *U*-tests (unpaired). Where appropriate data were corrected for multiple testing using the Benjamini–Hochberg procedure or local FDR estimation using R package fdrtools. For animal work, survival analysis was analyzed using a Grehan–Brislow–Wilcoxon test. These experiments were performed blind. A single animal was excluded from the study due to the development of skin lesions and total hair loss (alopecia) during EAE induction (in keeping with Hooke Lab EAE product information). Sample size was calculated in advance using the R pwr package and data collected from previous studies or pilots. Exact *n* values and statistical tests are reported in the figure legends.

### Reporting summary

Further information on research design is available in the Nature Portfolio Reporting Summary linked to this article.

## Online content

Any methods, additional references, Nature Portfolio reporting summaries, source data, extended data, supplementary information, acknowledgements, peer review information; details of author contributions and competing interests; and statements of data and code availability are available at https://doi.org/10.1038/s41588-026-02776-8.

## Supplementary information

Supplementary Information Supplementary Figs. 1–8, Notes 1 and 2, and Methods.

Reporting Summary

Peer Review File

Supplementary Tables 1–5 Supplementary Tables 1–5. Each table is provided on a separate worksheet.
Supplementary Table 1. Lead variants and linked variants from the GWASs used for locus selection.
Supplementary Table 2. MCC capture oligonucleotides.
Supplementary Table 3. SgRNAs, donor templates and primers.
Supplementary Table 4. Antibodies.
Supplementary Table 5. GEO accession nos. for publicly available datasets analyzed in this study.

Supplementary Data 1 Statistical source data for Supplementary Figs. 1, 2 and 5.

## Source data

Source Data Fig. 1 Statistical source data.

Source Data Fig. 2 Statistical source data.

Source Data Fig. 3 Statistical source data.

Source Data Fig. 4 Statistical source data.

Source Data Fig. 5 Statistical source data.

Source Data Fig. 6 Statistical source data.

Source Data Fig. 7 Statistical source data.

Source Data Extended Data Fig. 1 Statistical source data.

Source Data Extended Data Fig. 2 Statistical source data.

Source Data Extended Data Fig. 3 Statistical source data.

Source Data Extended Data Fig. 4 Statistical source data.

Source Data Extended Data Fig. 5 Statistical source data.

Source Data Extended Data Fig. 7 Statistical source data.

Source Data Extended Data Fig. 8 Statistical source data.

Source Data Extended Data Fig. 9 Statistical source data.

Source Data Extended Data Fig. 10 Statistical source data.

Source Data Fig. 7e & Extended Data FIg. 10k Unprocessed western blots.

## Data Availability

All sequencing data have been submitted to the NCBI’s Gene Expression Omnibus, accession nos. GSE301652, GSE301653, GSE301655, GSE301656 and GSE317575. Previously published data are available under the accession nos. listed in Supplementary Table 5. Processed nanoscale heatmaps are available via Zenodo at https://doi.org/10.5281/zenodo.18404259 (ref. ^43^). All data generated or analyzed during this study are included in this published article and Supplementary information. Source data are provided with this paper.

## Code availability

The MCC pipeline, peak caller and variant-to-function analysis are available for academic use through the Oxford University Innovation software store https://process.innovation.ox.ac.uk/software/p/16529a/micro-capture-c-academic/1. The SNP prioritization code is available at https://github.com/Davies-Genomics-and-Genome-Editing-Lab/snp_prioritization_pipeline (ref. ^69^). BasepairC can be found at https://github.com/jamesdalg/basepairC (ref. ^70^).
