# Supplementary material for: Single-allele nanoscale mapping of regulatory variants
Source: Nat Genet. 2026 Oct 2;58(10):2660–72. doi: 10.1038/s41588-026-02776-8 (PMC13649568; doi:10.1038/s41588-026-02776-8)
Supplement: Supplementary file 1 [file 41588_2026_2776_MOESM1_ESM.pdf]

nature genetics

Supplementary information https://doi.org/10.1038/s41588-026-02776-8

Single-allele nanoscale mapping of regulatory variants

In the format provided by the authors and unedited

Supplementary Information

Single-allele nanoscale mapping of regulatory variants.

Table of Contents

Supplementary Figures 3
- Supplementary Figure 1 3
- Supplementary Figure 2 4
- Supplementary Figure 3 5
- Supplementary Figure 4 6
- Supplementary Figure 5 7
- Supplementary Figure 6 8
- Supplementary Figure 7 9
- Supplementary Figure 8 10

Supplementary Notes 11
- Supplementary Note 1 11
- Supplementary Note 2 12

Supplementary Methods 13

Supplementary Figures

Supplementary Figure 1

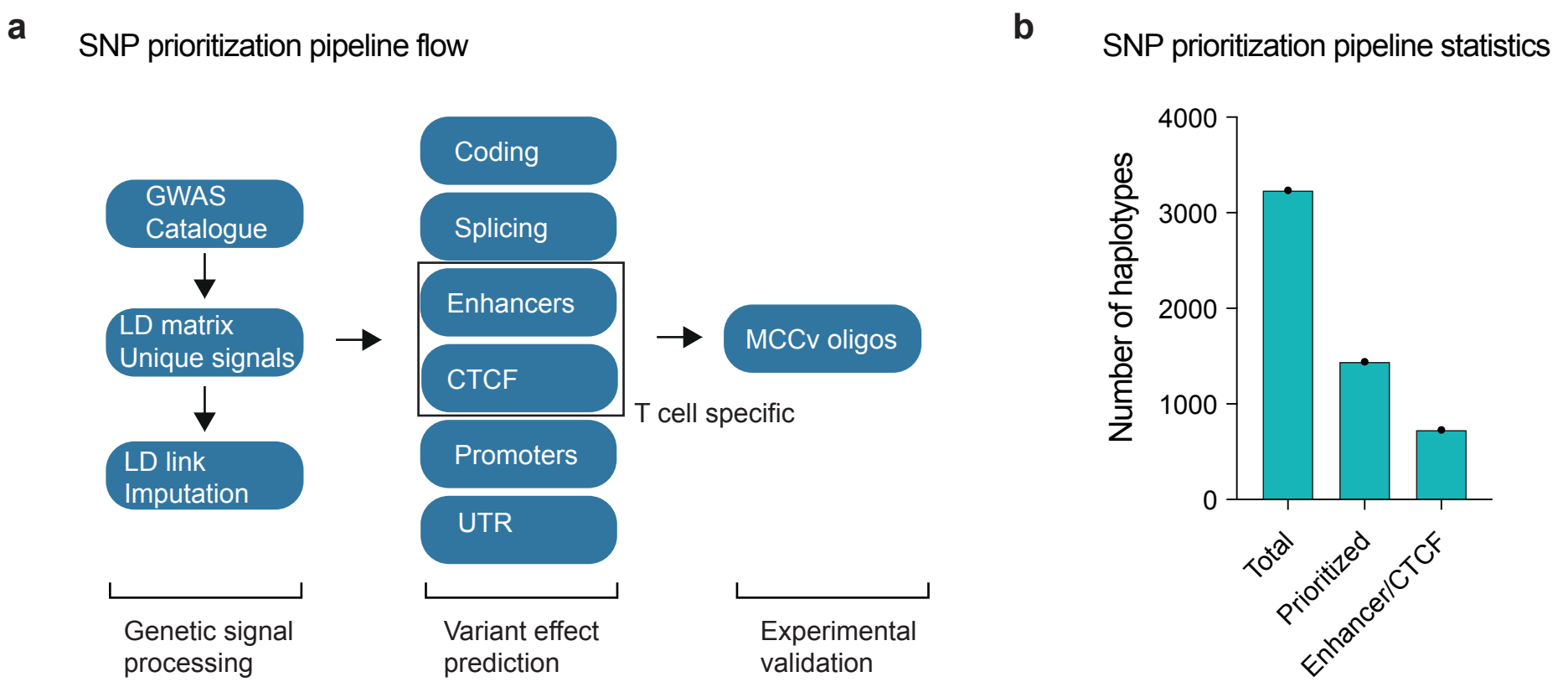

Supplementary Figure 1 | a, Schematic of the SNP prioritisation pipeline (see methods for more details). IMID-GWAS signals were collated from the GWAS catalogue, before imputation with LDlink[1]. Full haplotypes were assessed for coding (ANNOVAR[2]), splicing (spliceAI[3]), 3'UTR (miRDB[4]), promoter[5] (ATAC and CAGE-seq intersection), enhancer (ATAC-intersection) and CTCF variants (CTCF ChIP seq intersection). Enhancer and CTCF variants were chosen for MCCv-based follow-up. **b,** Summary of input and output numbers for SNP prioritisation pipeline. A total of 3238 IMID-GWAS signals were analysed, with 1441 having at least 1 SNP being identified in the six molecular mechanisms tested (coding, splicing, enhancer, CTCF, promoter, UTR). A total of 727 were classified as CTCF altering or enhancer altering in activated $CD4^+$/$CD8^+$ or Monocytes.

Supplementary Figure 2

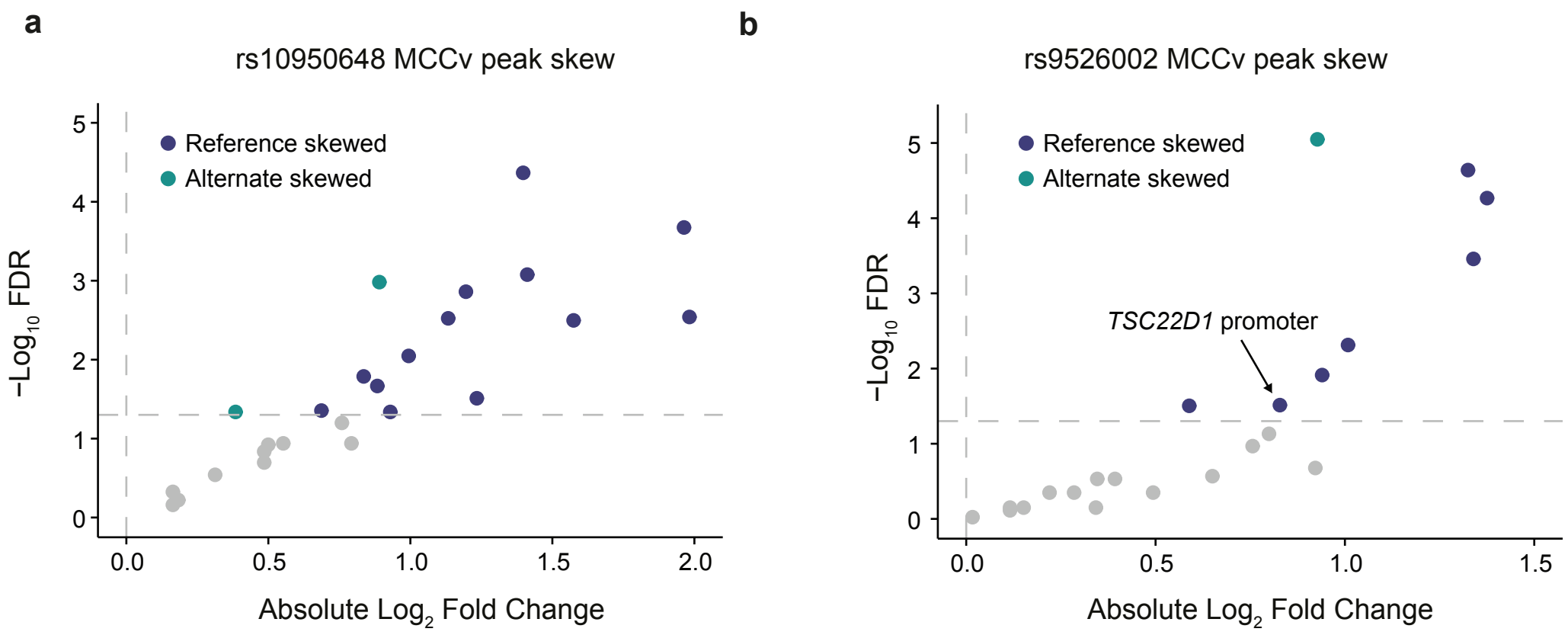

Supplementary Figure 2 | a, Absolute $\log_2$ fold change of junction counts between reference and alternate alleles at Lotron MCC called peaks for rs10950648 (n = 12, paired Student's t-test, two-sided, local FDR estimate with fdrtools). **b,** Absolute $\log_2$ fold change of junction counts between reference and alternate alleles at Lotron MCC called peaks for rs9526002 (n = 12, paired Student's t-test, two-sided, local FDR estimate with fdrtools).

Supplementary Figure 3

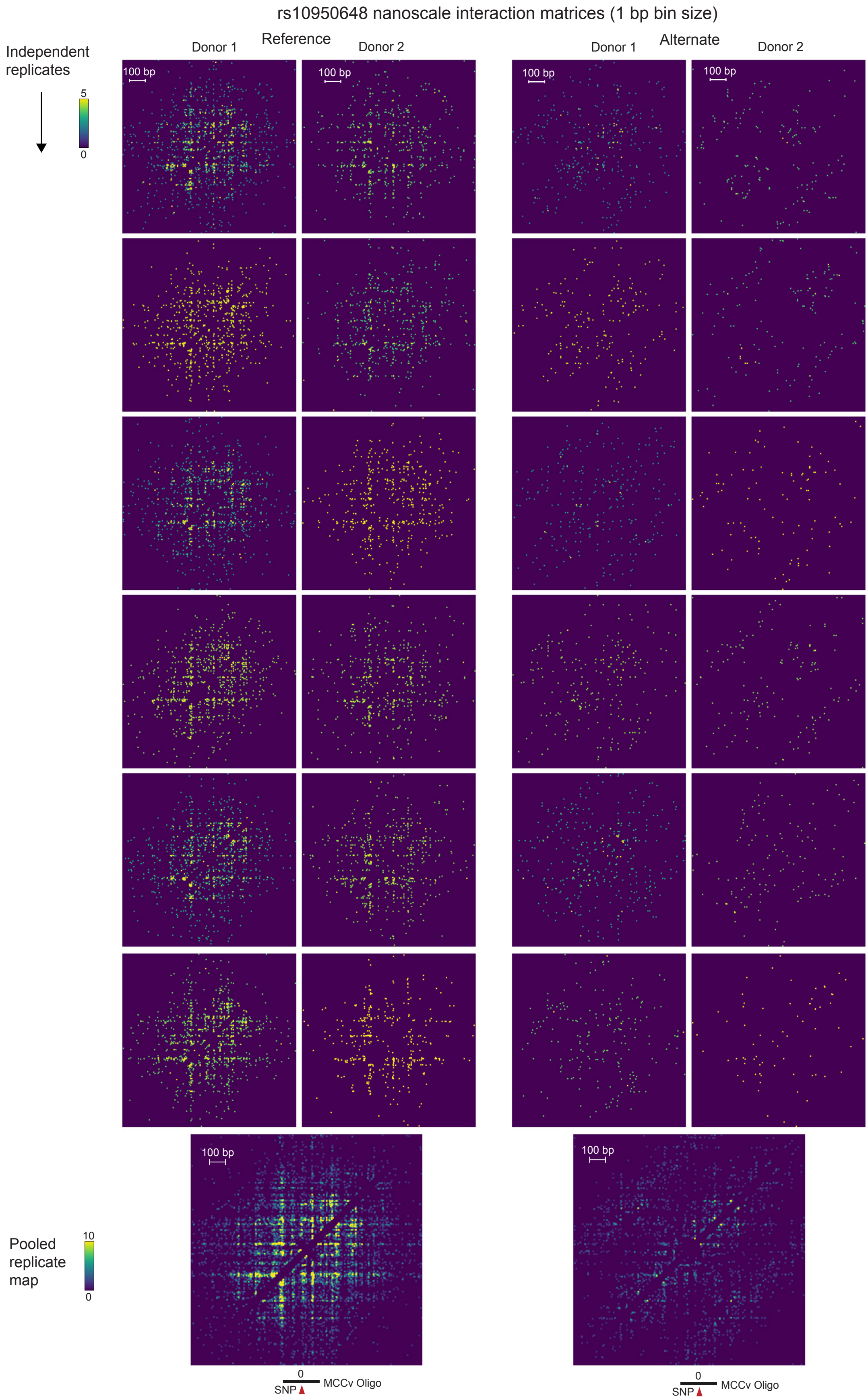

Supplementary Figure 3 | Nanoscale interaction matrices (400 bp window) from rs10950648 variant alleles (reference and alternate), including independent MCC replicates from the same donor (n = 6) across two donors (total n = 12). The final merged matrices are included for comparison.

Supplementary Figure 4

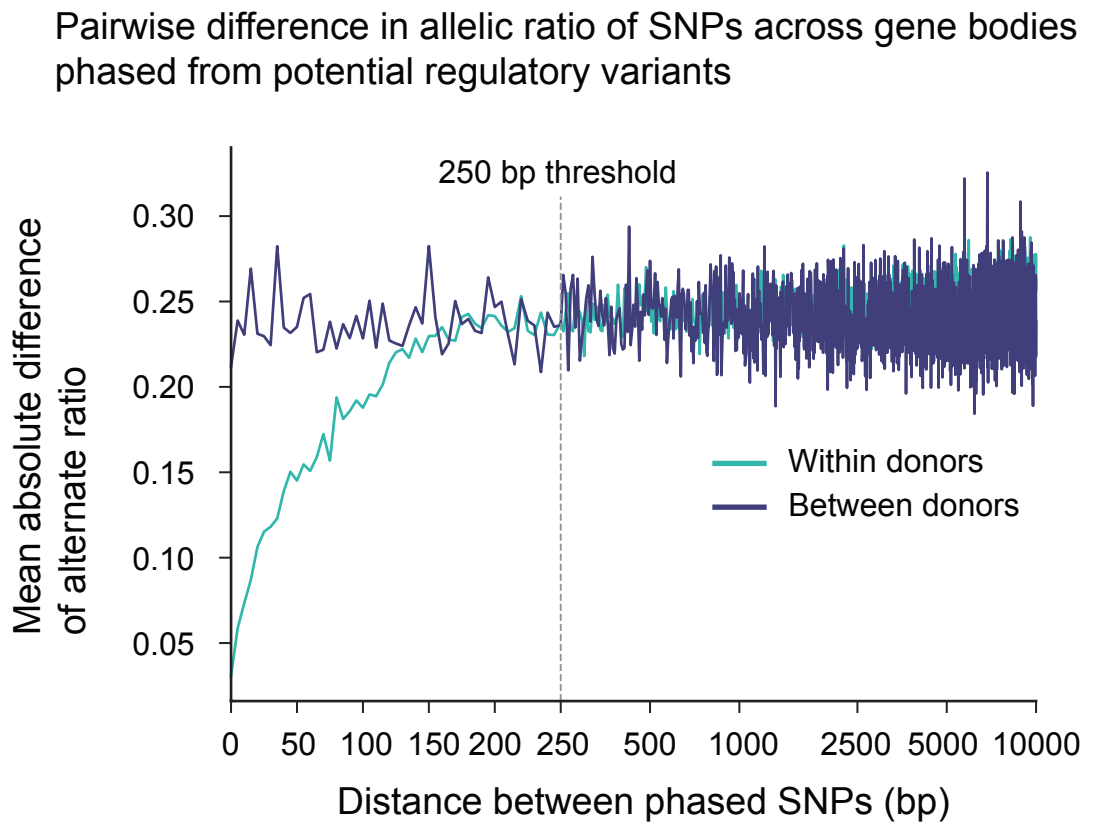

Supplementary Figure 4 | The absolute difference in alternate ratio (alternate counts/total counts) between pairs of SNPs with different distances within the same gene body. This was compared within single donors (green) and between donors (purple). The distance threshold cutoff used in the RNA skew analysis to define independent SNPs is shown as a dashed grey line. For each SNP-gene pair, all pairwise combinations of SNPs in the gene body were analysed within and between donors, and the absolute difference in the alternate ratio read fraction was computed for each pair. Pairs were binned by inter-SNP distance in 5 bp windows, points represent the mean absolute difference per bin.

Supplementary Figure 5

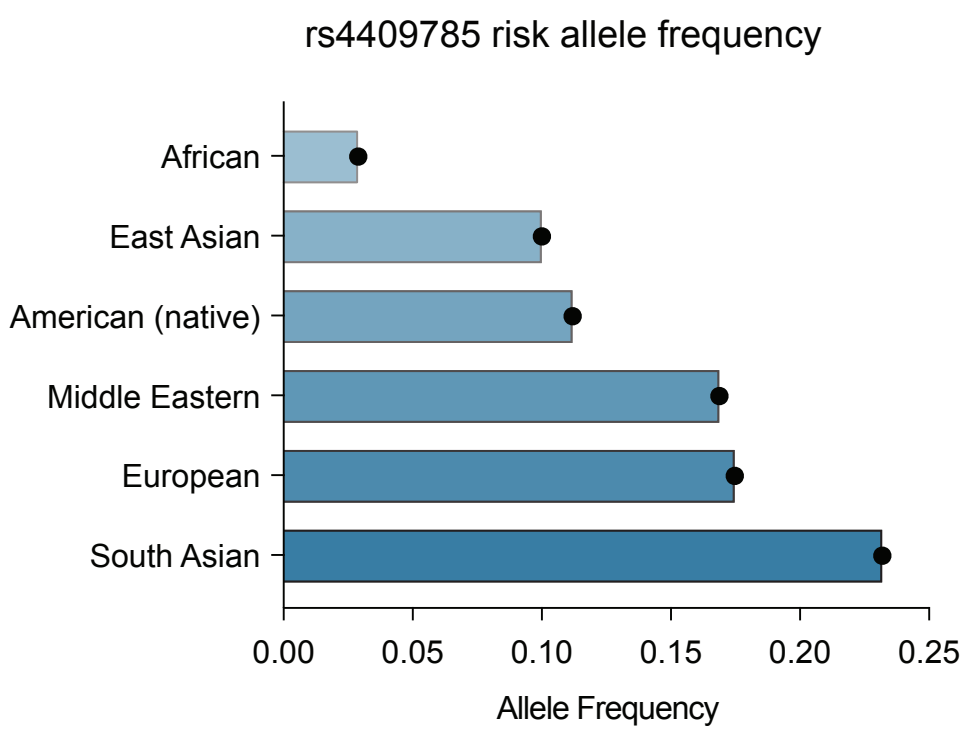

Supplementary Figure 5 | Calculated risk allele (alternate, C) frequency from PheWAS analysis in different genetic populations within the All of Us database for rs4409785.

Supplementary Figure 6

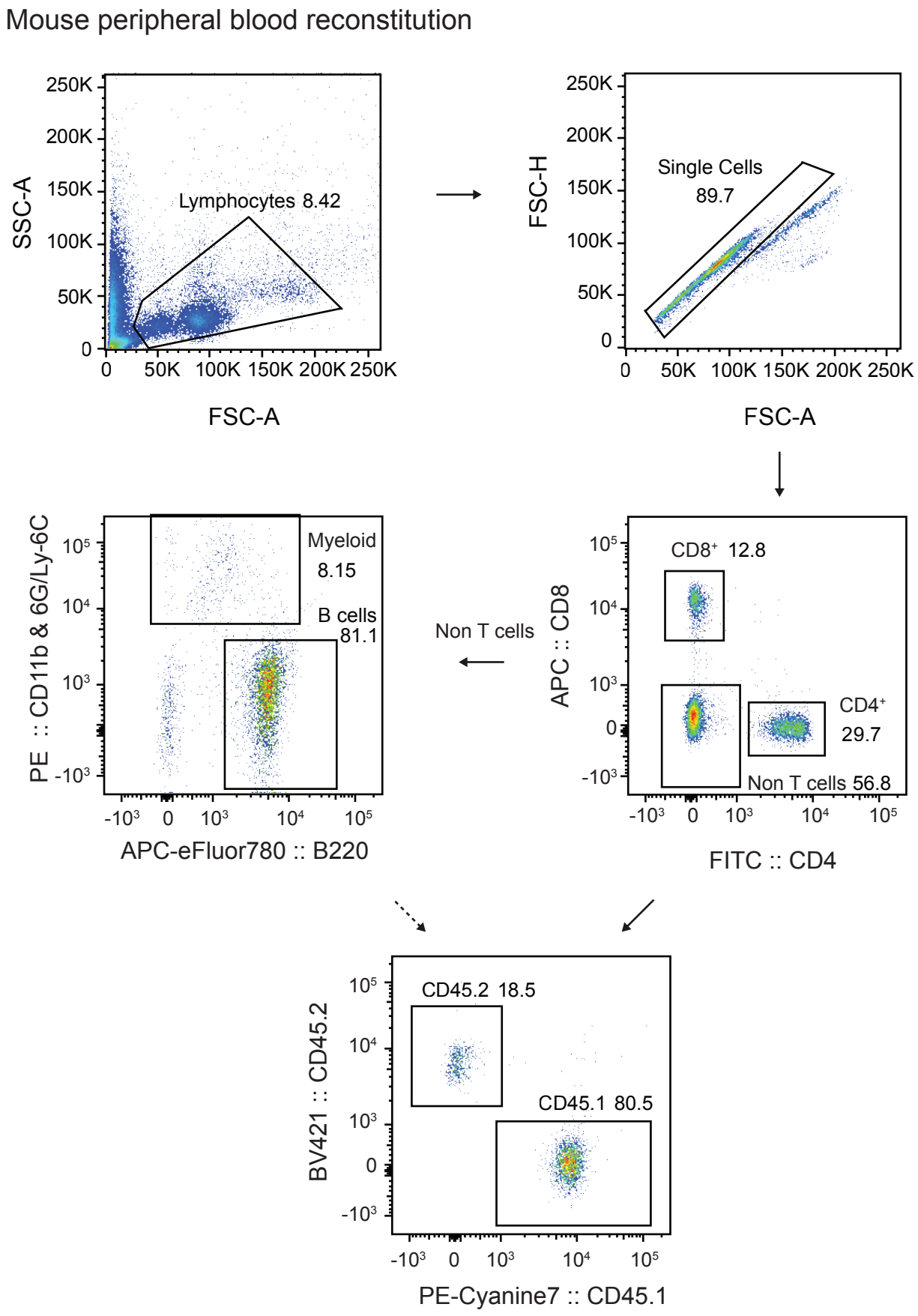

Supplementary Figure 6 | Flow cytometry gating strategy for peripheral blood reconstitution for **Extended Data Fig. 10b**. Lymphocyte singlets were gated into T cell and non-T cell subpopulations, whilst CD45 allele gates donor versus recipient cells.

Supplementary Figure 7

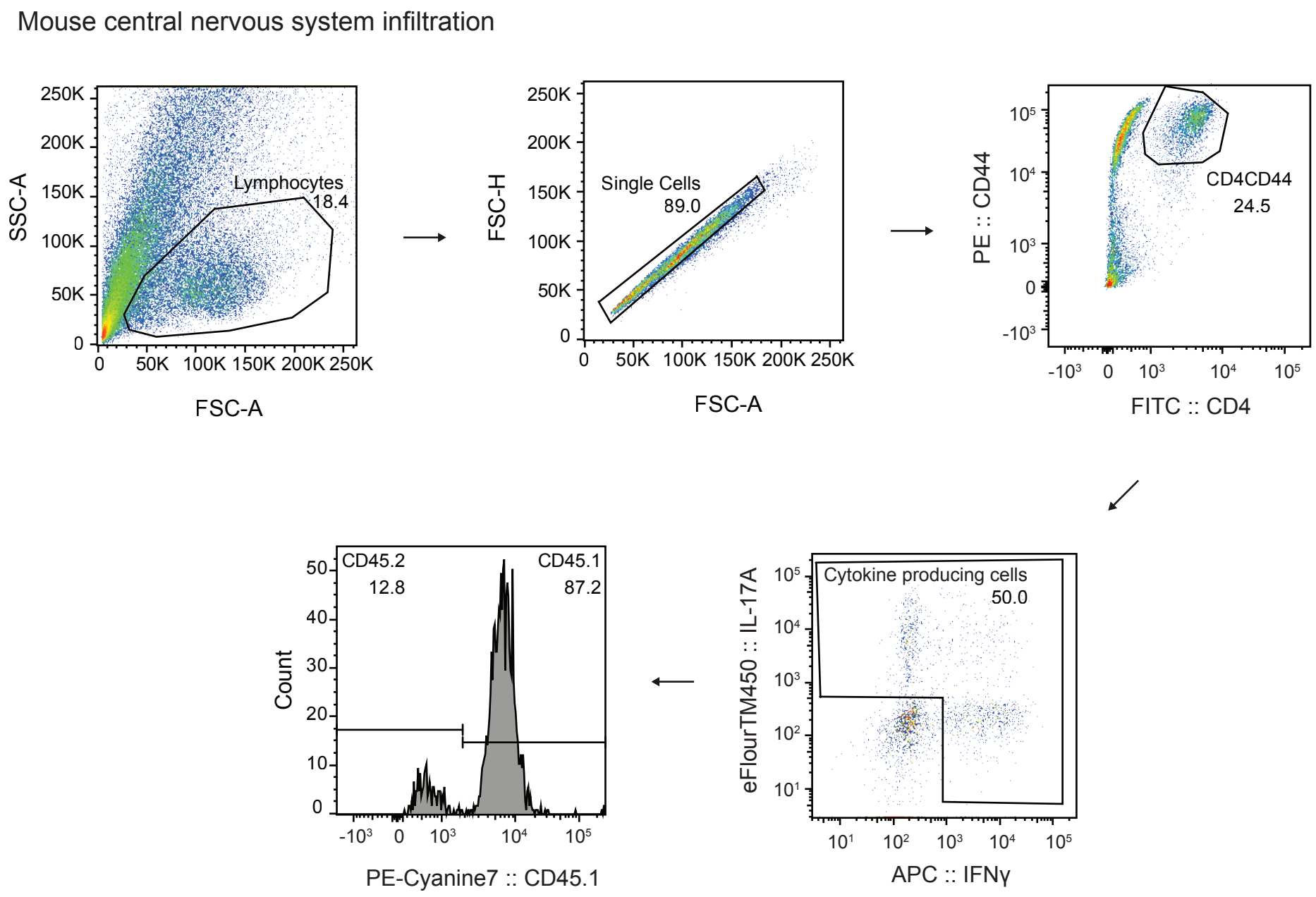

Supplementary Figure 7 | Flow cytometry gating strategy for mouse central nervous system infiltration by activated T cells for **Extended Data Fig. 10e**. Lymphocyte singlets were gated into cytokine-positive activated T cells.

Supplementary Figure 8

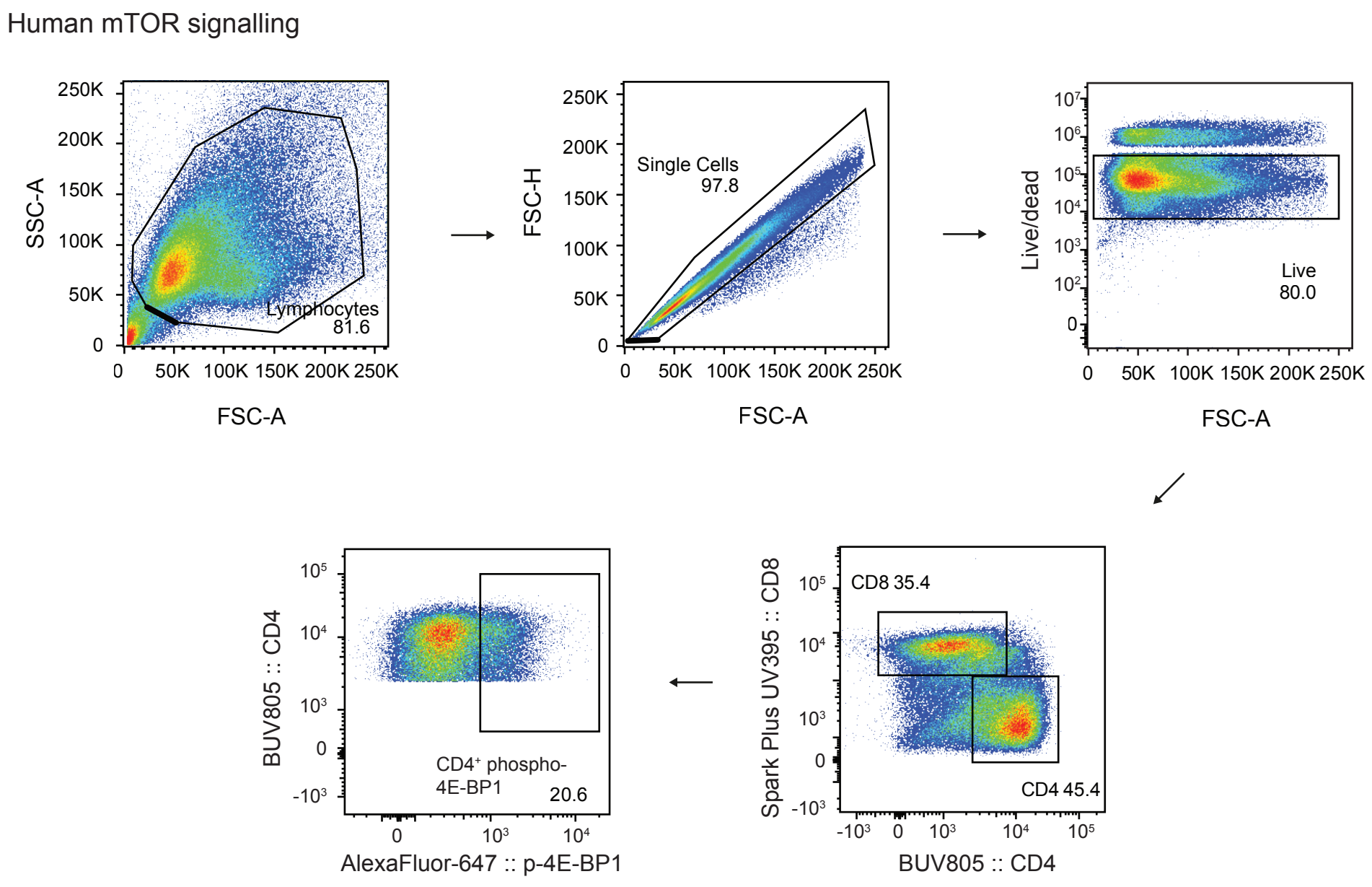

Supplementary Figure 8 | Flow cytometry gating strategy for human mTOR signalling in T cells for **Fig. 7d** and **Extended Data Fig. 10j.** mTOR signal was measured by the percentage of activated CD4 T cells positive for phospho-4E-BP1^{+}.

Supplementary Notes

Supplementary Note 1

Filtering used to calculate the enrichment of allelically imbalanced genes prioritised by MCCv

We aimed to demonstrate that genes linked to a variant which demonstrates changes in MCCv contacts are more likely to have imbalanced allelic expression.

1. We nominated 251 protein-coding genes from 405 variants. These were derived from the level of MCC contacts only, without utilising the single-allele components of the data. This nomination is based on the frequency of contact with the gene promoter. The drop to 251 is a mixture of three things – firstly, we could not detect significant contacts between 14% of SNPs and a gene promoter (due to a mixture of poor oligo probe capture efficiency, inclusion of CTCF variants, which often do not make contacts with promoters (e.g. *SESN3* variant) and inclusion of some non-T cell enhancer variants as controls). Of the remaining 347 variants, 77 (22.2%) contacted the same gene leaving 270 primary-nominated genes based on bulk 3C contact data and of these 251 are protein coding.
2. Figure 3f-g then details our exploration of single-allele analysis applied to the 241 (out of 405) variants, which were heterozygous in at least one donor.
3. For Figure 3f, we tested for allelic imbalance in paired RNA-seq data at all primary and secondary nominated genes linked to a heterozygous SNP in an enhancer. We tested 102 SNP gene pairs (from 88 SNPs) as certain variants had both primary and secondary genes pass phasing requirements. The drop from 241 heterozygous variants is due to our strict requirements for successful phasing of the enhancer variant with the gene-body haplotype (which required at least 25 gene-body SNPs separated by 250 bp).
4. Figure 3g details our exploration of whether variants with changes in 3C contact between alleles make it more likely that they will be linked to allelically imbalanced gene expression beyond background signal. First, we wanted to calculate a genome-wide background. For this, we calculated that within our donors an average of 10.0% of genes displayed allelic imbalance within the RNA-seq dataset. We also calculated a second background by estimating the percentage of genes which displayed allelic imbalance, which contacted variants with no evidence of allelic MCCv skew (8.9%, 17 out of 190 genes). This rate is similar if other criteria are used (for example, at loci where there is a detectable RNA skew and a change in chromatin structure with the primary or secondary gene, the rate of allelic RNA imbalance for other, more weakly contacting genes is 9.5%).
5. We then calculated the percentage of variant-gene pairs with changes in allelic MCC contacts and calculated enrichment scores relative to the genome-wide background. 125 of 241 heterozygous variants displayed evidence of 3C skew. These were intersected with the 102 MCC linked genes at which we could potentially detect RNA skew (i.e. contained at least 25 gene body SNPs). 56 of these genes were contacting a SNP with a change in MCC contacts and 46 were linked to unchanged SNPs. Overall, 35.5% (16 of 45, 1 in 2.81) of variants with a change in MCC contacts were associated with RNA skew at either the primary or secondary gene (30.3% of primary nominated and 26.1% of secondary nominated genes). Using the percentage of primary nominated genes with RNA skew linked to SNPs with 3C changes, this represents a 3.0-fold enrichment over the genome-wide background.
6. The 3.0-fold enrichment over background corresponds to a 33% false discovery rate (FDR). However, an FDR calculation requires a well-defined null, i.e. a background rate that reflects truly non-functional events. In this case, the observed ~10% genome-wide rate of allelic skew is unlikely to represent purely technical noise and likely includes a substantial proportion of biologically meaningful regulatory variation. As a result, this FDR is likely to be a conservative estimate of the true FDR.

Supplementary Note 2

Discussion of the MCCv throughput and bottlenecks

The number of variants that can be analysed in a single MCCv experiment is limited by the number of probes per capture. The limit of probes per capture is up to 1000[6], however the parallelised sequencing library preparation protocol means that each MCCv library can potentially be used in 10 or more capture reactions with different targets. This increases the potential throughput of the method, as separate probe-set captures can be performed simultaneously, with minimal impact on labour costs. Thus, for a given MCCv library, ~10 separate 1000 probe sets could be used, increasing the theoretical number of variant assays to 100,000. The main bottleneck of this approach will be the sequencing costs which will scale linearly with the number of variants. Variants require 100,000 reads for reliable MCCv coverage.

Supplementary Methods

Micro-C library generation, sequencing and analysis

Micro-C was performed using the Dovetail Micro-C Kit (v2.0 protocol for Tissue/Blood, May 2024) on 3x technical replicates of 1 million stimulated $CD4^+$ T cells isolated from a single donor. Crosslinking, chromatin digestion, end repair, and proximity ligation steps were carried out according to the manufacturer's instructions. Following reversal of crosslinks and DNA purification, sequencing libraries were prepared with supplied adapters and reagents and amplified via PCR. Libraries were sequenced by paired-end sequencing with a 150-cycle high-output NextSeq 500 kit (Illumina).

Sequencing data were processed following the Micro-C analysis guidelines described by the manufacturer. Paired-end reads were aligned to the hg38 reference genome using bwa-mem[7] with -5SP flags to support chimeric alignments. The output was piped through the pairtools suite: alignments were parsed with a minimum mapping quality of 40, sorted, deduplicated (with duplicate statistics collected), and split to generate a valid pairs file. In parallel, alignments were output as a sorted and indexed BAM file using samtools. Each technical replicate was sequenced to a minimum depth of 350 million paired-end reads. Valid pairs from all three replicates were merged to generate a combined contact map. Hi-C files were generated using Juicer tools[8] (v1.22.01), with the pre command.

Cell type selection

Genetic enrichment analysis of the lead SNPs to identify likely causal cell types was performed using CREST-GV[9]. In brief, lead SNPs from all GWAS signals were intersected with single-cell ATAC sequencing from PBMCs. An enrichment score was calculated as the degree of intersection in a specific cluster versus background.

ATAC-sequencing

150,000 cells were harvested, centrifuged at 500g for 5 minutes before being resuspended in ATAC lysis buffer (10 mM Tris-HCL, pH 7.5, 10 mM NaCl, 3 mM $MgCl_2$, 0.1% Igepal CA-630) and immediately span at 4°C for 10 minutes at 500g. The resulting nuclear pellets were PBS washed before being resuspended in a tagmentation buffer (20 mM Tris-HCl pH 7.5, 10 mM $MgCl_2$, 20% Dimethyl Formamide) with Tn5 transposase (Illumina, 20034197). This reaction was incubated for 30 minutes at 37°C. The reaction was quenched was Buffer PB of the Qiagen MinElute DNA purification kit (Qiagen 28004) and DNA was purified following the manufacturer's instructions. The purified DNA was then amplified using the Q5 high-fidelity mastermix (NEB M0491S) with Illumina Nextera Sequencing primers. Libraries were sequenced by paired-end sequencing with a 75-cycle high-output NextSeq 500 kit (Illumina).

Where indicated, ATAC-seq was combined with an MCC-based capture enrichment (see Oligonucleotide hybridisation, capture and sequencing). KAPA universal enhancing oligos (Roche, cat. No. 09075836001) included in the HyperCapture Target Enrichment Kit reagents (Roche, cat. No. 9075828001), were replaced with xGen™ Universal Blockers NXT (IDT, cat. No. 1079584) to match NextSeq-based library preparation of ATAC-seq libraries.

ATAC, RNA, ChIP-seq analysis

NGS libraries generated in-house for this manuscript or downloaded from GEO were analysed using the CATCH-UP pipeline[10]. In brief for ATAC and ChIP-seq data, reads were trimmed with Trim Galore (Babraham Institute), before being aligned with Bowtie2[11]. Aligned BAMs were deduplicated with Picard before being converted to BigWigs using Deeptools[12] BamCoverage and visualized on the UCSC genome browser[13]. Peaks were called using Lanceotron[14]. For RNA-seq analysis reads were aligned with STAR[15]. In both cases, the hg38 genome build was used for alignment.

ATAC-seq data were split by allele using pysam. ATAC footprints were generated using TOBIAS[16] with default parameters with allele-split bam files as inputs, before being visualised on the UCSC genome browser as BigWigs.

Statistical analysis of MCCv nanoscale matrices

At each locus, MCCv matrices were first read in by basepairC.core::read_replicates, but no filtering was applied at this point (lower threshold was set to zero and upper threshold was set to infinity). These matrices were made symmetric, equalising read counts with read pairs on either side of the diagonal to ensure that reads found at a given pair were given the same read counts regardless of order. These matrices were combined into a single matrix with one column per sample, with two columns for location 1 and location 2 in the matrix. Counts and percentages of nonzero pixels were tabulated to give an indication of sparsity and sample quality. After reading the data, the matrices were library normalised to equalize mean and variance between samples to ensure that a fair comparison was made without regard to sample bias using the trimmed mean of M-values (TMM) algorithm[17] with filtering option set to "none". A long, deduplicated data frame was constructed containing columns for sample, allele, and counts.

In order to determine the size of a practically significant effect, we used a simulation to estimate the size of a randomly generated one. A simulation was done using a sham treatment that was constructed by randomly shuffling vectors that were 50% 0 and 50% 1 to produce a random allele assignment (sham treatment) that mirrored the experimental design. This was done ten times and random seeds were saved for reproducibility. Model starting values were selected from an average using bootstrapping 1% of the sample, with 5 bootstraps to increase convergence speed. A quasi-Poisson generalized linear model was then modelled using the counts as the dependent variable and the randomly assigned allele as the covariate (sham treatment). The individual sham treatment β-coefficients and standard errors across these 10 randomly assigned models were saved and averaged to give a representation of an effect size that would be produced by random sample bias rather than true signal. Following this, a standard quasi-Poisson GLM was used to compare the true data, using the counts as the outcome and the binary allele (reference or not) as the covariate of interest. The quasi-Poisson GLM was chosen to ensure that overdispersion did not affect the conclusions of the analysis, similar to a negative binomial GLM, while using a glm framework (speedglm) that more stably handles larger datasets than the standard glm.nb can.

MCCv peak quantification comparison

Merged MCCv bigWig tracks for each SNP were peak called with LotronMCC. Peaks were then filtered using a custom python script (> 1.5 kB from the capture site, > 50 bp in width, and within 2 mB of the capture point). Peaks were used as windows for counting the number of PCR-deduplicated unique junctions per allele. These counts were normalised to the total number of *cis*-unique ligation junctions from the corresponding viewpoint in the reference and alternate allele respectively. On average, variant alleles have 20% differences in total *cis*-unique ligation junctions.

Variants whose total *cis* junction count difference exceeded this range would be further investigated for technical problems in the experiment usually resulting in exclusion from the analysis or down-sampling of the allele. This normalisation step is intended to control for global differences in sequencing depth and overall ligation junction capture between alleles, including differences driven by MNase accessibility which has been extensively explored previously[18]. As a result, the log-fold-change values reported likely reflect relative differences in the distribution of contacts across peaks, rather than absolute differences in the total number of contacts generated by each allele. Statistically significant allele-specific effects arise primarily when there is a redistribution of contacts, such that specific peaks show a disproportionate gain or loss of ligation junctions relative to the total *cis*-contact signal for that allele.

Per experiment, a minimum of six independent MCC libraries are generated, resulting in a 6 by 6 comparison of junction counts per allele, which were compared statistically using a paired two-sided Student's t-test. A Kolmogorov-Smirnov test was used to assess normality of count data at a given peak between alleles. To account for multiple hypothesis testing, we estimated local false discovery rates using the fdrtool R package[19]. Nominal *p*-values were supplied directly to the fdrtool function (statistic = "pvalue"), which models the empirical null distribution to infer local FDRs.

Validation of causal gene nomination strategy

To assess the biological plausibility of the inferred causal gene lists, we tested for over-representation of GO Biological Process terms using the clusterProfiler R package[20]. Enrichment significance was assessed by over-representation testing as implemented in clusterProfiler::enrichGO, with Benjamini–Hochberg correction applied to control the false discovery rate. Causal gene lists were assessed for essentiality using the DepMap database[21], classification as a drug target or approved drug using the OpenTargets database[22], and whether they have a known mutation leading to a primary immunodeficiency (Genomics England). A background set of genes (all genes ± 1 Mb from captured SNPs) was similarly assessed to estimate an enrichment of our nominated gene list.

Preparation of T cell base editing components

In vitro transcription templates for the BE4max were generated using PCR amplification of the BE4max plasmid (pCMV_BE4max) using primer IVT-F and IVT-R (Supplementary Table 3) with Q5 high-fidelity master mix (NEB M0492) according to manufacture's instructions. BE mRNA was transcribed using the HiScribe T7 mRNA Kit with CleanCap Reagent AG (NEB E2080S). To suppress the innate immune response in the T cells after nucleofection, uridine was replaced with N1-methylpseudouridine (Trilink). Single guide RNAs to the CTCF and control sites were bought from Synthego with 2'-O-methyl analogs (OMe) on the first and last three bases and 3' phosphorothioate internucleotide linkages (PS) between the first three and last two bases (Supplementary Table 3).

Mouse cKit$^+$ isolation, culture and editing

Mouse cKit$^+$ cells were isolated and cultured as previously described[23,24]. Briefly, 8–12-week-old B6.SLJ-Ptprca Pepcb/BoyJ donor mice were culled and bone-marrow cells were isolated from tibia, femur and pelvis, before staining with a c-Kit antibody. Enrichment was achieved with anti-APC magnetic beads and LS columns (Miltenyi Biotec). CKit+ cells were cultured in a serum-

albumin-free polyvinyl alcohol (PVA) based system using a medium composed of F12 medium, 1% ITSX, 10mM HEPES, 1% P/S/G, 100 ng ml^{-1} mouse TPO, 10 ng ml^{-1} mouse SCF and 0.1% PVA (P8136, 363081). Cells were cultured in CellBIND 24-well plates (Corning) at a density of 1 x 10^6 cells per ml. Media changes were done every 2-3 days. After 7 days in culture, 1 x 10^6 cells were nucleofected with the P3 kit (Lonza) with 30 µg of Alt-R S.p. Cas9 Nuclease V3 (IDT) and 16µg of sgRNA designed to Sesn3 or Rosa26 with the EO100 program. Cells were immediately transferred back into $cKit^+$ media and after 6 hours a media change was conducted to wash out the P3 solution. After 24 hours, editing efficiency was measured with locus specific NGS primers as previously described.

Mouse CNS infiltration

For the CNS infiltration flow-cytometry, mononuclear cells from the CNS were cultured in RPMI (Gibco) supplemented with 10% FBS, BD GolgiStop™ (BD Biosciences) and BD GolgiPlug™ (BD Biosciences), and stimulated with 10 ng/ml phorbol 12-myristate 13-acetate (PMA) and 1 µg/ml ionomycin for 6 hours prior to staining. Cells were then blocked with Rat anti-mouse CD16/CD32 (BD Biosciences). Intracellular cytokine stainings were performed using the Cytofix/Cytoperm Fixation and Permeabilization kit (BD Biosciences) with FITC-anti-CD4 (eBioscience, 11-0042-85) and PE anti-CD44 (eBioscience, 12-0441-82), PE-Cyanine7 anti-mouse CD45.1 (BioLegend, 110730), APC anti-mouse IFN-gamma (eBioscience, 17-7311-82) and eFluor 450 anti-mouse IL-17A (eBioscience, 48-7177-82) (Supplementary Fig. 8 and Supplementary Table 4). All the stained cells were analysed with a BD LSRFortessa X-20 cell analyser.

Endogenous tagging of *SESN3*

A 3X FLAG tagged *SESN3* Jurkat cell line was generated using homology-directed genome editing with asymmetric single-stranded donor oligonucleotides (ssODNs)[25]. Briefly, 100 pmole of gRNA, 110 pmole of ssODN donor, 100 pmole ALT-R spCas9 (IDT 1081058), and 1 µL of HDR enhancer v2 (IDT 10007910) were mixed and incubated at room temperature for 10 min. 200,000 Jurkat cells were resuspended in 20 µL of prepared Lonza SE cell line solution with supplement (Lonza V4XC-1032) before being added to the HDR mix. This was transferred to a 20 µL Lonza cuvette well and nucleofected using CL-120 preset. Nucleofected cells were transferred into 1 mL of growth medium (see Methods) and allowed to recover for 7 days. Cells were FACS sorted into single colonies in a 96-well plate. Clones were genotyped using tag flanking primers followed by Sanger sequencing. A final clone was chosen based on tagged protein levels by Western blotting.

Supplementary References

1. Machiela, M.J. & Chanock, S.J. LDlink: a web-based application for exploring population-specific haplotype structure and linking correlated alleles of possible functional variants. *Bioinformatics* **31**, 3555-7 (2015).
2. Wang, K., Li, M. & Hakonarson, H. ANNOVAR: functional annotation of genetic variants from high-throughput sequencing data. *Nucleic Acids Research* **38**, e164-e164 (2010).
3. Jaganathan, K. *et al.* Predicting Splicing from Primary Sequence with Deep Learning. *Cell* **176**, 535-548.e24 (2019).
4. Chen, Y. & Wang, X. miRDB: an online database for prediction of functional microRNA targets. *Nucleic Acids Res* **48**, D127-D131 (2020).
5. Dreos, R., Ambrosini, G., Cavin Perier, R. & Bucher, P. EPD and EPDnew, high-quality promoter resources in the next-generation sequencing era. *Nucleic Acids Res* **41**, D157-64 (2013).
6. Downes, D.J. *et al.* High-resolution targeted 3C interrogation of cis-regulatory element organization at genome-wide scale. *Nat Commun* **12**, 531 (2021).
7. Li, H. & Durbin, R. Fast and accurate short read alignment with Burrows-Wheeler transform. *Bioinformatics* **25**, 1754-60 (2009).
8. Durand, N.C. *et al.* Juicer Provides a One-Click System for Analyzing Loop-Resolution Hi-C Experiments. *Cell Syst* **3**, 95-8 (2016).
9. Riva, S.G. *et al.* CREST-GV: Cell types Ranking and Enrichment Score for selecTive Genetic Variants. *medRxiv*, 2025.07.29.25332311 (2025).
10. Riva, S.G., Georgiades, E., Gur, E.R., Baxter, M. & Hughes, J.R. CATCH-UP: A High-Throughput Upstream-Pipeline for Bulk ATAC-Seq and ChIP-Seq Data. *Journal of visualized experiments : JoVE* (2023).
11. Langmead, B. & Salzberg, S.L. Fast gapped-read alignment with Bowtie 2. *Nature methods* **9**, 357–359 (2012).
12. Ramírez, F., Dündar, F., Diehl, S., Grüning, B.A. & Manke, T. deepTools: a flexible platform for exploring deep-sequencing data. *Nucleic Acids Research* **42**, W187-W191 (2014).
13. Raney, B.J. *et al.* The UCSC Genome Browser database: 2024 update. *Nucleic Acids Research* **52**, D1082-D1088 (2024).
14. Hentges, L.D. *et al.* LanceOtron: a deep learning peak caller for genome sequencing experiments. *Bioinformatics* **38**, 4255-4263 (2022).
15. Dobin, A. *et al.* STAR: ultrafast universal RNA-seq aligner. *Bioinformatics (Oxford, England)* **29**, 15-21 (2013).
16. Bentsen, M. *et al.* ATAC-seq footprinting unravels kinetics of transcription factor binding during zygotic genome activation. *Nat Commun* **11**, 4267 (2020).
17. Lun, A.T. & Smyth, G.K. csaw: a Bioconductor package for differential binding analysis of ChIP-seq data using sliding windows. *Nucleic Acids Res* **44**, e45 (2016).
18. Hua, P. *et al.* Defining genome architecture at base-pair resolution. *Nature* **595**, 125-129 (2021).
19. Strimmer, K. fdrtool: a versatile R package for estimating local and tail area-based false discovery rates. *Bioinformatics* **24**, 1461-2 (2008).
20. Wu, T. *et al.* clusterProfiler 4.0: A universal enrichment tool for interpreting omics data. *Innovation (Camb)* **2**, 100141 (2021).
21. Pacini, C. *et al.* A comprehensive clinically informed map of dependencies in cancer cells and framework for target prioritization. *Cancer Cell* **42**, 301-316 e9 (2024).
22. Koscielny, G. *et al.* Open Targets: a platform for therapeutic target identification and validation. *Nucleic Acids Res* **45**, D985-D994 (2017).
23. Wilkinson, A.C. *et al.* Long-term ex vivo haematopoietic-stem-cell expansion allows nonconditioned transplantation. *Nature* **571**, 117-121 (2019).
24. Wilkinson, A.C., Ishida, R., Nakauchi, H. & Yamazaki, S. Long-term ex vivo expansion of mouse hematopoietic stem cells. *Nature Protocols* **15**, 628-648 (2020).
25. Richardson, C.D., Ray, G.J., DeWitt, M.A., Curie, G.L. & Corn, J.E. Enhancing homology-directed genome editing by catalytically active and inactive CRISPR-Cas9 using asymmetric donor DNA. *Nat Biotechnol* **34**, 339-44 (2016).
