# Supplementary material for: Single-allele nanoscale mapping of regulatory variants
Source: Nat Genet. 2026 Oct 2;58(10):2660–72. doi: 10.1038/s41588-026-02776-8 (PMC13649568; doi:10.1038/s41588-026-02776-8)

Unprocessed western blot gel images

Figure 7e

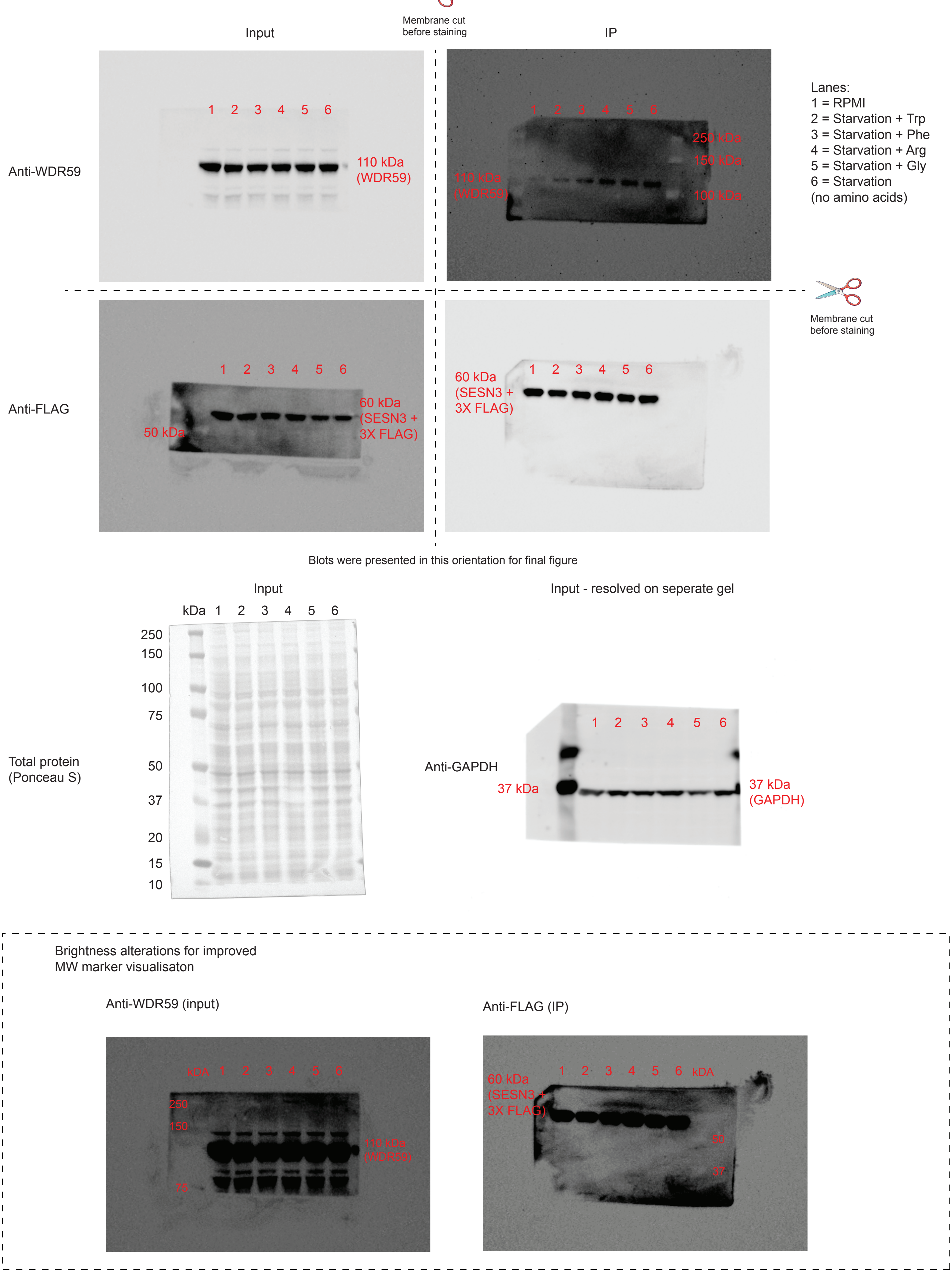

Extended Data Figure 10k

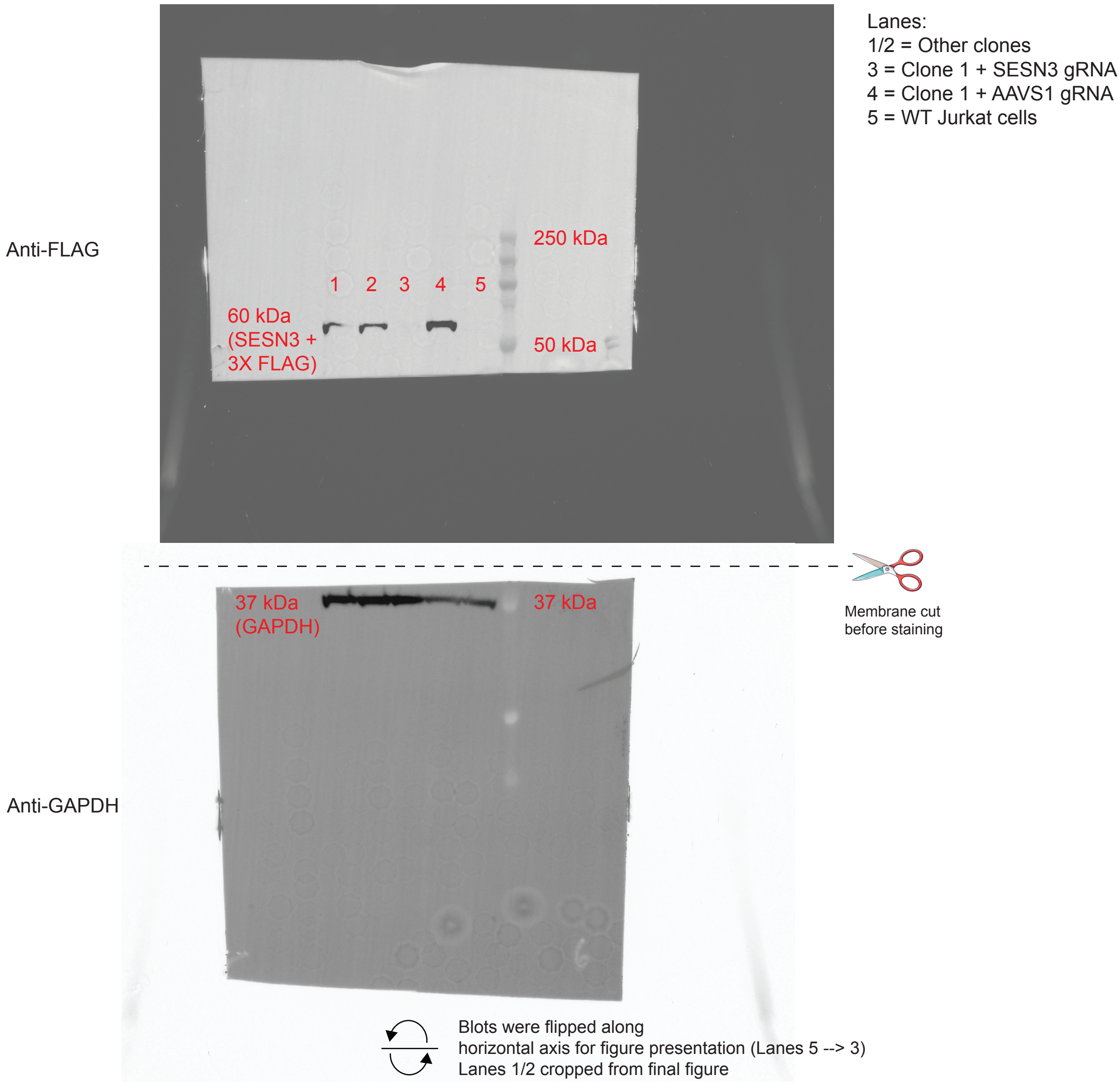

Supplement: Supplementary file 2 [file 41588_2026_2776_MOESM22_ESM.pdf]
