# Supplementary material for: Single-allele nanoscale mapping of regulatory variants
Source: Nat Genet. 2026 Oct 2;58(10):2660–72. doi: 10.1038/s41588-026-02776-8 (PMC13649568; doi:10.1038/s41588-026-02776-8)
Supplement: Supplementary file 3 [file 41588_2026_2776_MOESM2_ESM.pdf]

nature portfolio

Corresponding author(s): Thomas Milne, Jim Hughes, James Davies

Last updated by author(s): Jul 24, 2026

# Reporting Summary

Nature Portfolio wishes to improve the reproducibility of the work that we publish. This form provides structure for consistency and transparency in reporting. For further information on Nature Portfolio policies, see our Editorial Policies and the Editorial Policy Checklist.

## Statistics

For all statistical analyses, confirm that the following items are present in the figure legend, table legend, main text, or Methods section.

| n/a | Confirmed | |
|---|---|---|
| ☐ | ☒ | The exact sample size (*n*) for each experimental group/condition, given as a discrete number and unit of measurement |
| ☐ | ☒ | A statement on whether measurements were taken from distinct samples or whether the same sample was measured repeatedly |
| ☐ | ☒ | The statistical test(s) used AND whether they are one- or two-sided<br>*Only common tests should be described solely by name; describe more complex techniques in the Methods section.* |
| ☒ | ☐ | A description of all covariates tested |
| ☐ | ☒ | A description of any assumptions or corrections, such as tests of normality and adjustment for multiple comparisons |
| ☐ | ☒ | A full description of the statistical parameters including central tendency (e.g. means) or other basic estimates (e.g. regression coefficient) AND variation (e.g. standard deviation) or associated estimates of uncertainty (e.g. confidence intervals) |
| ☐ | ☒ | For null hypothesis testing, the test statistic (e.g. *F*, *t*, *r*) with confidence intervals, effect sizes, degrees of freedom and *P* value noted<br>*Give P values as exact values whenever suitable.* |
| ☒ | ☐ | For Bayesian analysis, information on the choice of priors and Markov chain Monte Carlo settings |
| ☒ | ☐ | For hierarchical and complex designs, identification of the appropriate level for tests and full reporting of outcomes |
| ☒ | ☐ | Estimates of effect sizes (e.g. Cohen's *d*, Pearson's *r*), indicating how they were calculated |

*Our web collection on statistics for biologists contains articles on many of the points above.*

## Software and code

Policy information about availability of computer code

| | |
|---|---|
| Data collection | Next generation high throughput sequencing (Illumina platoform) , flow cytometry (FACSDiva 8.0 LSRII Fortessa) |
| Data analysis | -No new algorithms were created in this study.<br>- MCC was processed with TrimGalore (v0 .. 3.1), FLASH (vl.2.11), BLAT (v35), bowtie2 (v2.3.5), MCCsplitter.pl (vl) and MCCanalyser.pl (vl). MCCsplitter.pl and MCCanalyser.pl are available for academic use through the Oxford University Innovation software store https://processinnovation.ox.ac.uk/software/p/16529a/micro-capture-c-academic/1.<br>- Scripts used for allele-specific MCC analysis (MCC_SNP_splitter.py) are available for academic use through the Oxford University Innovation software store https://processinnovation.ox.ac.uk/software/p/16529a/micro-capture-c-academic/1.<br>- Peak calling for MCC data using Lotron MCC is available for academic use through the Oxford University Innovation software store https://processinnovation.ox.ac.uk/software/p/16529a/micro-capture-c-academic/1.<br>- Nanoscale heatmaps were compared using basepairC, which has been made available at https://github.com/jamesdalg/basepairC<br>- ATAC, ChIP, and RNA sequencing was processed using the CATCH-UP pipeline (https://github.com/Genome-FunctionInitiative-Oxford/UpStreamPipeline/ tree/main/genetics/CATCH-UP).<br>- Allelic skew for the aforementioned methods was performed using bcftools mpileup (v1.9).<br>- Micro-C was analysed using bwa-mem, pairtools, Juicer tools (v1.22.01).<br>- Amplicon sequencing for CRISPR editing efficiencies were analysed with CRISPRESSO v2 (http://crispresso2.pinellolab.org).<br>- lmmunofluorescence images were processed in Fiji (v2.l.0, https://imagej.net/Fiji).<br>-Downstream GWAS signal processing was conducted using the SNP_prioritization pipeline (https://github.com/Davies-Genomics-and-Genome-Editing-Lab/snp_prioritization_pipeline) which utilises publicly available tools such as LDlink API (https://ldlink.nci.nih.gov/), annovar (https://annovar.openbioinformatics.org/en/latest/), spliceAI (v1.3.1), pybigwig (v0.3.22), pybedtools (v0.9.1).<br>- Statistical analysis was performed in prism v10 and python package SciPy (v1.17.1) |

-Flow cytometry were analysed with FlowJo (v 10).
-Power calculations were performed using the R pwr package.

For manuscripts utilizing custom algorithms or software that are central to the research but not yet described in published literature, software must be made available to editors and reviewers. We strongly encourage code deposition in a community repository (e.g. GitHub). See the Nature Portfolio guidelines for submitting code & software for further information.

## Data

Policy information about availability of data

All manuscripts must include a data availability statement. This statement should provide the following information, where applicable:

- Accession codes, unique identifiers, or web links for publicly available datasets
- A description of any restrictions on data availability
- For clinical datasets or third party data, please ensure that the statement adheres to our policy

All sequencing data have been submitted to the NCBI Gene Expression Omnibus: (GSE301652, GSE301653, GSE301655, GSE301656, GSE31755). Previously published data are available under the accession codes listed (Supplementary Table 5). Processed nanoscale heatmaps have been uploaded to Zenodo (https://doi.org/10.5281/zenodo.18404259). All data generated or analysed during this study are included in this published article and its supplementary information. Source data are provided with this paper.

## Research involving human participants, their data, or biological material

Policy information about studies with human participants or human data. See also policy information about sex, gender (identity/presentation), and sexual orientation and race, ethnicity and racism.

| | |
|---|---|
| Reporting on sex and gender | Findings are expected to apply equally to both males and females. |
| Reporting on race, ethnicity, or other socially relevant groupings | No information on race or ethnicity of study participants was obtained. |
| Population characteristics | All samples were obtained from healthy donors. |
| Recruitment | Healthy volunteers age 18-70, were recruited from the local population. There was no immediate benefit from partaking in the study and self-selection bias was not expected to impact the results of the study. |
| Ethics oversight | Peripheral blood was collected from healthy donors with written consent and ethical approval (REC Ref. no. R8800/RE001 and R45381/RE001). ) from the University of Oxford Medical Sciences Interdivisional Research Ethics Committee |

Note that full information on the approval of the study protocol must also be provided in the manuscript.

# Field-specific reporting

Please select the one below that is the best fit for your research. If you are not sure, read the appropriate sections before making your selection.

☒ Life sciences ☐ Behavioural & social sciences ☐ Ecological, evolutionary & environmental sciences

For a reference copy of the document with all sections, see nature.com/documents/nr-reporting-summary-flat.pdf

# Life sciences study design

All studies must disclose on these points even when the disclosure is negative.

Sample size

For the Micro Capture-C experiments in cell lines, 3 flasks of cells were fixed on which 2 independent experiments were performed with different concentrations of MNase for a total of 6 replicates. This sample size was chosen to allow for statistical analysis and to control for different concentrations of MNase as well as to maximise the sensitivity of the assay. For the primary cell data 3 healthy biological donor activated CD4+ T cells were fixed. To these 6 independent library replicates (with differing MNase concentrations) were performed for a total of 18 MCC libraries. This was in order to maximise the sensitivity and resolution of the assay when performing single allele data analysis. For the editing MCCv experiment 6 independent replicates from 1 biological donor was performed on the mixed population of edit outcome cells. Micro-C was performed on 1 biological donor with three technical replicate experiments (starting post-fixation). MCC ultra experiments were performed on six independent replicates from 1 biological donor. Three experiments of poly A minus RNA was performed on 3 biological healthy donors. For ex vivo mTOR signalling experiments data was collected from 5 independent healthy donors over two experiments. For in vitro gene editing efficiency studies of T cells, the sample size reflects the number of gene editing replicates performed and was sufficient to determine the overall editing efficiency according to previous studies (DOI: 10.1038/s41586-021-03609-w). All peripheral blood samples from biological donors were obtained with ethical approval by venesection from volunteers without self-reported autoimmune or other chronic immunological/inflammatory disorders recruited through the Investigation of Haematopoiesis in Healthy Volunteers Study (CUREC1 R88300/RE001).

For in vitro editing of mouse HSCs, the sample size reflects pooling all mouse HSCs used in each individual batch of transplantation. For all

| | |
|---|---|
| | mouse experiments sample sizes were calculated in advance using the R pwr package and data collected from previous studies or pilot data. For clinical score and weight observed during EAE induction, the sample size indicates the number of mice involved in the induction. For studies related to samples collected from mouse, the sample size reflects the number of mice dissected for sample collection. |
| Data exclusions | Mice that developed skin lesions and total hair loss (alopecia) during EAE induction were culled and excluded from analysis in keeping with the Hooke Lab EAE product information. In the ex vivo T cell experiments, donor T cells which did not expand during the activation phase were excluded. No other data was excluded in this study. |
| Replication | Whenever possible, all studies were done in multiple biological replicates. For Micro Capture-C data 3 biological donors were used with 6 replicates to ensure higher sensitivity of the assay. Quality control was performed largely through measurement of DNA concentrations and fragment sizes during experiments and samples were only taken forward for sequencing when there was evidence of good digestion and ligation with 3C library manufacture and fragment sizes were correct during sequencing library preparation and oligonucleotide capture. Micro-C and RNA data generated were replicated three times for each biological donor. Immunoblotting was replicated at least 2 times. Flow cytometry data was replicated at least two times and within each experiment multiple biological donors were used and separate nucleofections were performed. |
| Randomization | For human genomic data random grouping of samples was not relevant to this study as comparison was between distinct cell-types, tissues, and genotypes. All mice involved in the study were randomly divided into each individual groups. Mice from each group were premixed before experiments to minimize cage effects. |
| Blinding | For human studies, blinding was not relevant to the study as groupings were predetermined by inherent cell-type or genotype and thus not subject to bias. Generation and processing of data followed identical protocols or pipelines for all samples to minimize bias. For mice studies, each mice were given a unique ID before sample harvesting and processing. Mice from each group were premixed before experiments to minimum cage effects and provide a blinded fashion during scoring pf EAE induced mice. |

# Reporting for specific materials, systems and methods

We require information from authors about some types of materials, experimental systems and methods used in many studies. Here, indicate whether each material, system or method listed is relevant to your study. If you are not sure if a list item applies to your research, read the appropriate section before selecting a response.

## Materials & experimental systems

| n/a | Involved in the study | |
|---|---|---|
| ☐ | ☒ | Antibodies |
| ☐ | ☒ | Eukaryotic cell lines |
| ☒ | ☐ | Palaeontology and archaeology |
| ☐ | ☒ | Animals and other organisms |
| ☒ | ☐ | Clinical data |
| ☒ | ☐ | Dual use research of concern |
| ☒ | ☐ | Plants |

## Methods

| n/a | Involved in the study | |
|---|---|---|
| ☒ | ☐ | ChIP-seq |
| ☐ | ☒ | Flow cytometry |
| ☒ | ☐ | MRI-based neuroimaging |

## Antibodies

Antibodies used

Details are also provided in the manuscript. The following antibodies were used: antigen (Clone), company (Catalog number), Conjugation (Lot, Concentration).

FACS:
Mouse data –
For antibodies used in flow cytometry on mouse PBMCs and splenocytes: mouse CD4 (RM4-5), ebioscienceTM (11-0042-85), FITC (11-0042-85, 1:100); mouse CD45.1 (A20), Biolegend (110730), PE-cyanine7 (B357466, 1:100); mouse CD44 (IM7), ebioscienceTM (12-0441-82), PE (2489317, 1:100); mouse CD11b (M1/70), ebioscienceTM (12-0112-82), PE (2460179, 1:200); mouse Ly-6G/Ly-6C (RB6-8C5), ebioscienceTM (12-5931-82), PE (2416206, 1:200); mouse IL-17A (2653028), ebioscienceTM (48-7177-82), eFluorTM 450 (eBio1787, 1:200); mouse IFN gamma (XGM1.2), ebioscienceTM (17-7311082), APC (2660425, 1:200); mouse B220 (RA3-6B2), ebioscienceTM (47-0452-82), APC-eFluor 780 (2410100, 1:200); mouse 45.2 (104), Biolegend (109832), BV421 (B368573, 1:200).
Human data –
For antibodies used in flow cytometry on human T cells: CD8 (RPA-T8), Biolegend (301079), Spark Plus UV395 (B431920, 1:100); Viability (NA), Biolegend (423107), Zombie UV (B422234, 1:500); CD4 (SK3), BD Biosciences (612888), BUV805 (4057007, 1:100); CD45RA (HI100), Biolegend (304141), BV510 (B422231, 1:25); CD69 (FN50), Biolegend (310903), FITC (B417168, 1:50); pS6 (D57.2.2E), Cell signaling (5316S), PE (12, 1:50); CCR7 (G043H7), Biolegend (353235), PE/Dazzle594 (B428210, 1:25); p-4E-BP1 (236B4), Cell signaling (5123S), AlexaFluor-647 (10, 1:50); HLADR (L243), Biolegend (307625), AF-700 (B407512, 1:100).

Immunoblotting:

Antibodies used for FLAG IP in human Jurkat cells: Flag M2 (1:1000), Sigma (F1804); WDR59 (D4Z7A, 1:1000), Cell Signalling Technology (53385S); GAPDH (1:8000), Cell Signaling (97166S); Anti-Mouse HRP (1:10000), Sigma (A4416); Anti-Rabbit HRP (1:10000), Sigma (A6667); Anti-Mouse Fluorescent (1:10000), Abcam (ab2167765).

Validation | All antibodies are communally purchased and have been validated by the manufacturers.

# Eukaryotic cell lines

Policy information about cell lines and Sex and Gender in Research

| | |
|---|---|
| Cell line source(s) | Cell line name: Jurkat clone E6-1 Source: ATCC (TIB-152), Passage:8-20. |
| Authentication | Authentication was not undertaken but cell lines were obtained from widely used sources. |
| Mycoplasma contamination | All cell lines were tested for mycoplasma contamination by PCR and the results are all negative. |
| Commonly misidentified lines (See ICLAC register) | NA – Jurkat clone E6-1 is not a commonly misidentified line. |

# Animals and other research organisms

Policy information about studies involving animals; ARRIVE guidelines recommended for reporting animal research, and Sex and Gender in Research

| | |
|---|---|
| Laboratory animals | Animal species: Mus musculus<br>Strain: C57BL/6J (Transplant and EAE)<br>Strain: B6.SLJ-Ptprca Pepcb/BoyJ (HSC extraction)<br><br>The animals used in EAE studies were typically 9-13 weeks old. The animals used for extracting HSCs were typically 8-12 weeks old. The animals used as recipient for mouse HSC transplant were typically 3-4 weeks old. |
| Wild animals | NA |
| Reporting on sex | All in the animals involved in EAE studies were females. The animals used for extracting HSCs were typically males. |
| Field-collected samples | NA |
| Ethics oversight | EAE and HSC transplant studies were performed under the project license PP8589020 approved by the UK home office. |

Note that full information on the approval of the study protocol must also be provided in the manuscript.

# Plants

| | |
|---|---|
| Seed stocks | NA |
| Novel plant genotypes | NA |
| Authentication | NA |

# Flow Cytometry

## Plots

Confirm that:

☒ The axis labels state the marker and fluorochrome used (e.g. CD4-FITC).

☒ The axis scales are clearly visible. Include numbers along axes only for bottom left plot of group (a 'group' is an analysis of identical markers).

☒ All plots are contour plots with outliers or pseudocolor plots.

☒ A numerical value for number of cells or percentage (with statistics) is provided.

## Methodology

| | |
|---|---|
| Sample preparation | For mouse cell flow cytometry: single mononuclear cell suspensions derived from Spleen, CNS and draining lymph nodes were stained for 30 minutes in FACS buffer (2% FBS, 5mM EDTA in PBS) containing surfacing antibodies at optimal |

concentration listed in the antibody section. Then cells used for detecting surface antigen were centrifuged at 300g for 5 minutes at 4 degree and washed twice with FACS buffer. For cells used for intracellular cytokine detection, cells were fixed and permeabilised according to the manufacturer's instruction (BD BioSciences Cytofix/Cytoperm, #554714); for intracellular staining, antibodies were incubated in permeabilization buffer for 30 minutes at 4 degrees.
For human T cell flow cytometry: single mononuclear cell suspensions derived from T cell cultures were stained for 15 minutes in the dark at room temperature. Cells were then washed in FACS buffer (PBS, 2% FBS). Cells were then fixed, permeabilised and intracellularly stained using using the True-Nuclear Transcription Factor buffer set (Biolegend, 424401) following manufacturer's instructions. In brief, 200ul of TrueNuclearFix 1X was applied for 45 mins in the dark at room temperature before two 200ul wash True-Nuclear™ 1X Perm Buffer. Intracellular stain was performed in True-Nuclear 1x Perm Buffer for 30 mins at room temperature in the dark. Cells were washed and resuspended in 200ul of FACS for being acquired on the LSR Fortessa X50.

Instrument

BD LSR II, BD LSR Fortessa X50.

Software

Data collection - FACSDiva 8.0 LSRII Fortessa
Data analysis - FlowJo V10

Cell population abundance

N/A - no sorting was performed during this study.

Gating strategy

Example gating strategies are provided in supplementary data file. Generally, all cells were gated on FSC and SSC to exclude debris. Then, single cells were gated using FSC-A and FSC-H.
For cells used to study reconstitution rate in PBMCs /spleen/draining lymph nodes after transplant, T cells were gated out with CD4 and CD8 marker, B cells were gated out with marker B220 while myeloid cells were gated out with Ly-6C/Ly-6G and CD11b. For cells used in intracellular staining, activated T cells were gated out via high expression of CD4 and CD44 marker.
For human T cell studies mTOR+ T cells were gated on CD4 and activation markers CD69/CD45RA/HLADR, before gating on p-4E-BP1.

☒ Tick this box to confirm that a figure exemplifying the gating strategy is provided in the Supplementary Information.
