# Supplementary material for: Single-allele nanoscale mapping of regulatory variants
Source: Nat Genet. 2026 Oct 2;58(10):2660–72. doi: 10.1038/s41588-026-02776-8 (PMC13649568; doi:10.1038/s41588-026-02776-8)
Supplement: Supplementary file 4 [file 41588_2026_2776_MOESM3_ESM.pdf]

nature portfolio

Peer Review File

Single-allele nanoscale mapping of regulatory variants

Corresponding Author: Professor James Davies

Version 0:

Decision Letter:

5th Nov 2025

Dear James,

Your Article, "Single-allele nanoscale mapping of regulatory variant function", has now been seen by 3 referees. I sincerely apologise for the long review process.

You will see from the reviewers' comments below that while they find your work of interest, some important points are raised. We are interested in the possibility of publishing your study in Nature Genetics, but would like to consider your response to these concerns in the form of a revised manuscript before we make a final decision on publication.

Please note that while reviewer #3 has suggested potentially removing the CTCF part of the paper, this is optional. While perhaps a bit preliminary, I personally find these results interesting. Please let me know if you'd like to discuss this further.

We therefore invite you to revise your manuscript taking into account all reviewer and editor comments. Please highlight all changes in the manuscript text file. At this stage we will need you to upload a copy of the manuscript in MS Word .docx or similar editable format.

We are committed to providing a fair and constructive peer-review process. Do not hesitate to contact me if there are specific requests from the reviewers that you believe are technically impossible or unlikely to yield a meaningful outcome. I would be happy to discuss the reviewers' comments in detail.

When revising your manuscript:

*1) Include a “Response to referees” document detailing, point-by-point, how you addressed each referee comment. If no action was taken to address a point, you must provide a compelling argument. This response will be sent back to the referees along with the revised manuscript.

*2) If you have not done so already please begin to revise your manuscript so that it conforms to our Article format instructions, available
here.
Refer also to any guidelines provided in this letter.

*3) Include a revised version of any required Reporting Summary: https://www.nature.com/documents/nr-reporting-summary.pdf
It will be available to referees (and, potentially, statisticians) to aid in their evaluation if the manuscript goes back for peer review.
A revised checklist is essential for re-review of the paper.

Please be aware of our guidelines on digital image standards.

EXTENDED DATA FIGURES

When re-submitting your manuscript, please ensure that any supplementary figures and tables that are crucial to the manuscript’s conclusions are converted into Extended Data figures and tables to increase visibility of these data. Extended Data figures and tables are online-only (present in the online PDF and full-text HTML versions of the paper), peer-reviewed display items that provide essential background to the article but are not included in the main article due to space

constraints. A maximum of ten Extended Data display items (figures and tables) is permitted.

Please use the link below to submit your revised manuscript and related files:

Link Redacted

Note: This URL links to your confidential home page and associated information about manuscripts you may have submitted, or that you are reviewing for us. If you wish to forward this email to co-authors, please delete the link to your homepage.

We hope to receive your revised manuscript within four to eight weeks. If you cannot send it within this time, please let us know.

Please do not hesitate to contact me if you have any questions or would like to discuss these revisions further.

Nature Genetics is committed to improving transparency in authorship. As part of our efforts in this direction, we are now requesting that all authors identified as ‘corresponding author’ on published papers create and link their Open Researcher and Contributor Identifier (ORCID) with their account on the Manuscript Tracking System (MTS), prior to acceptance. ORCID helps the scientific community achieve unambiguous attribution of all scholarly contributions. You can create and link your ORCID from the home page of the MTS by clicking on ‘Modify my Springer Nature account’. For more information please visit please visit www.springernature.com/orcid.

We look forward to seeing the revised manuscript and thank you for the opportunity to review your work.

Sincerely,

Tiago

Tiago Faial, PhD
Chief Editor
Nature Genetics
https://orcid.org/0000-0003-0864-1200

Reviewers' Comments:

Reviewer #1 (Remarks to the Author):

This study by Hamley et al. reports the extension of Micro Capture-C to study noncoding variants (“MCCv”) and its application to study variants associated with inflammatory diseases. The authors show that analyzing allele-specific 3D contacts (through hybrid capture of genomic regions containing variants) can identify effects on noncoding variants both at the ‘nanoscale’ (nearby nucleosomes, ~500bp) and at longer range (kilobases to megabases). The authors apply MMCv to ~400 variants associated with inflammatory diseases that overlap enhancers in activated human T cells, and illustrate numerous examples where the 3D contact links an element to a gene and where the allele-specific information shows an association with the variant. One such locus is the SESN3 locus, where a risk variant creates a new CTCF motif that blocks a super-enhancer from contacting the SENS3 promoter. Knocking out Sens3 in mouse T cells affects disease phenotypes in the EAE mouse model of autoimmunity, and the authors conduct experiments to show that this may be mediated by changes in mTOR signaling and tryptophan sensing.

Altogether this is an exciting study. The MCC data looks very clean and have some striking effects from allele-specific analysis. The results for the SENS3 locus appear to reveal some novel function of this gene in autoimmunity, and illustrate what can be learned by applying MMCv to study GWAS variants. Overall, well done!

Before publication, however, additional analyses should be conducted and major improvements made to the description and/or application of statistical and computational methods, and to the analysis of GWAS variants:

1. The GitHub link in the ‘Code Availability’ section is not publicly accessible. It should be made so for review, and may have helped to answer some of the following questions. That said, the Methods should also be more detailed and not rely on the Code to provide all of these answers.

1a. A central point in this paper is comparing 3D contacts between two alleles in the same cell population. However, I did not find any methods description of how the authors are quantifying changes in contacts, e.g. between two alleles or between two viewpoints. Fig. 1g appears to present the results of a ‘paired t-test’, but there is no description that I can find that explains what is being quantified or what is being paired. An ‘FDR correction’ is cited, but the tool cited appears to have various parameters and the methods do not describe what parameters were used.

1b. Beyond Fig. 1g, there do not appear to be many statistics reported regarding the magnitude or significance of differences for specific contacts (e.g., Fig 2c, Fig. 3d,e, etc.). In many cases the magnitude of the difference seems large and likely robust. Nonetheless this should be quantified

1c. The GWAS variant prioritization involves selecting GWAS signals that include variants that overlap enhancers or CTCF sites. Again, details for this analysis are very light. Extrapolating from Fig 2b and the Methods, it seems that the approach for selection was to look at GWAS loci where all variants within r^2 of 0.7 overlap enhancers or CTCF sites (and not have variants overlapping coding variants or splice sites), and then select those variants that overlap. How are CTCF sites and enhancers defined?

1d. 'We used a variant-promoter contact frequency ranking to nominate 251 unique causal genes' (Line 185) — What does that mean, exactly? Take the single promoter per locus with the strongest contact frequency?

2. The authors should conduct additional analysis of the variants they selected for study, the variants that had allelic effects in MCCv, and the 251 genes prioritized. In general, for these results to be actionable for downstream experiments or pre-clinical investigation, it is critical to estimate how enriched they are versus some appropriate background. Because of confounding from linkage disequilibrium, the fact that contact != regulation, and the multi-way relationships between enhancers and genes, it could be very common that variants have allele-specific effects on 3D contacts and link to genes that are not causally related to disease.

2a. For the selected variants — authors should report the enrichment of GWAS variants by their definition (e.g., r^2 > 0.7) in each category (enhancers, CTCF sites, coding, splice site), versus e.g. the set of all common variants. E.g. Enrichment = (Fraction of considered GWAS variants that overlap enhancers in T cells) / (Fraction of all common variants that overlap enhancers in T cells)

2b. For the variants that have allelic effects — Are there control sets of variants that were selected, such that a similar calculation could be performed? Alternatively, if there is fine-mapping available for any of the variants studied, you could show the 'Fraction of high-probability variants that have allelic effects' vs 'Fraction of low-probability variants that have allelic effects'

2c. The 251 'unique causal genes' should be similarly evaluated. E.g. Extended Data Fig. 2i reports the number that Approved Drug targets, but not the enrichment. For example, Enrichment here could be calculated as 'Fraction of prioritized genes that are Drug targets' / 'Fraction of all candidate genes in these loci … e.g. all genes within 1Mb, perhaps… that are Drug targets'. It would also be useful to compare these to predictions from other prediction methods, such as OpenTargets L2G and PoPS (Weeks et al. Nature Genetics)

3. Could the authors explain how exactly we should interpret the allele-specific data, in light of any statistical normalization or differences in MNase accessibility between alleles? For example, if allele A is more accessible to MNase and therefore creates more total contacts than allele B, but has the identical profile how is that represented/interpreted in the log-fold-change allelic analysis?

Other comments and questions:

• '10 million human genomes have been sequenced' (page 3). Citation?
• 'more enhancer activity' (page 5). What are you defining as enhancer activity, here? 'more accessibility' might be more precise here, if I understand correctly
• "minimizes experimental noise" (line 145) — I'm not sure I caught what this is referring to
• Capture oligos were designed either centered around the SNP or the peak of the regulatory element (Methods, page 17). Can you provide some rationale for why to design one way or another in the future?
• Fig. 2f — define 'Gene Ratio'?

Reviewer #1 (Remarks on code availability):

The code was not available for review - the link provided was not valid.

Reviewer #2 (Remarks to the Author):

In their paper, Hamley et al. describe a technology called MCCv that was developed to exploit high-res 3D genome conformation measurements to help identify functional genetic variants (e.g. SNPs) in regulatory elements. The latter remains a major challenge in the field, and hence this is an important pursuit.

The authors have a long-standing interest in this topic, which started with the Hughes et al. 2014 Capture-C paper in which they also used improved C-technology to link SNPs to putative target genes. Now they take advantage of their more recently developed MCC technique (PMID: 34108683). Major advance of MCC is its basepair resolution, which allows for the generation of allele-specific maps and combined phasing of other omics data.

The overall approach of using (high-res) 3D genome conformation to link disease-associated SNPs to candidate causal genes is very valid but not necessarily very novel. However, the authors do take this approach to a next level by tailoring their recently developed MCC technology to the ‘SNP-gene assigning problem’. Technically, the work is very impressive and robust, leaving this reviewer with little doubt about the added value of the MCCv platform to the field. The paper contains mostly anecdotal evidence from selected loci, but this is fine considering the topic and the aim to showcase the power of the technology. The authors finish with a more in-depth analysis of the function of a GWAS/MCCv hit (SESN3), which in my opinion is a bit weaker than the rest of the manuscript.

Comments:
- Although MCCv is a very valuable addition for post-GWAS follow-up studies, the authors to my opinion often overstate the novelty of the methodology itself. In the end, MCCv is MCC with a few modifications, and I think it doesn’t need to be ‘hyped’ all the time in that way.
- The authors generate SESN3-KO primary CD4+ T cells, but then seem to switch back to Jurkat cells for the biochemistry. Fair enough for showing the protein-protein interactions, but this functional follow-up part would be much more convincing if the authors could directly show altered responses of these SESN3-KO T cells to relevant activation signals.
- I find the model drawn in Fig.6k to be confusing. The GWAS variant leads to a moderate reduction in SESN3 – that’s clear – but how does that then lead to excessive/chronic immune activation? Because that’s the expected outcome of the variant given its risk-increasing association with auto-immune diseases.
- Lines 378-380: I actually believe GWAS variant with gain-of-function outcomes have been described before (e.g. PMID: 29056226). Seems better to rephrase.

Reviewer #3 (Remarks to the Author):

This manuscript consists of two major parts. First, it describes MCCv, a new take on capture-microC to identify potentially functional sequence variants and their target gene(s) in the human genome. The second part dives deep into a single SNP that was found to disrupt/create a CTCF site, studying its effects by genome editing and even mouse experiments. Below I will discuss the MCCv section in detail. While the CTCF part in itself is well done and interesting, I feel that it is not a good idea to combine the two parts in one paper. In my view, there is substantially more work to be done to further validate and benchmark the MCCv technology (see below); the manuscript should focus on this and not be overloaded/side-tracked with the biology of a particular variant CTCF site - this will be a nice story on its own. Less can be more!

The MCCv approach has two major aspects. First, like other high-resolution "C" methods, it can identify distal contacts of a putative regulatory element. As was shown before, "C" methods can be allele-specific; here, by applying capture-microC (previously reported by the authors and other labs) with probes that pull down sets of pre-selected SNPs, the authors explore whether they can identify cis-linked genes that may be differentially regulated by the SNP variants. While some earlier papers have explored "C" methods for this purpose, the capture approach presented here seems to provide superior resolution. Second, the manuscript emphasises observed "nano-scale" changes in 3D structure immediately around the SNP. This is highlighted as a novelty in the beginning of the manuscript (and in title/abstract), but plays only a minor role later on (and I did not find it compelling, see below). A third useful feature of the method is that the data can also be used to phase the probed genomic loci, which makes it possible to link the data to allele-specific gene expression.

As the authors mention in their introduction, it is still a challenge to identify functional non-coding SNPs and to identify the target genes. So, in principle, there is a need for new approaches, and MCCv may be a good addition to the noncoding SNP annotation toolbox. However, I have several technical concerns at this point. Most importantly, systematic benchmarking of the method is needed.

Major points:

1. Much of the manuscript leans on the assumption that 3D contacts between distal elements are predictive of functional regulatory interactions. While there are certainly a lot of anecdotes that support this, the critical question here is: how reliable is this assumption? Of 100 of such contacts, how many have an actual effect on gene expression? Is it 2, 10, 50 or 90%? Are most contacts functionally relevant, or mere bystander effects? This question is relevant here, because the MCCv method is based on the contact-equals-function assumption. The current manuscript does not do a good job to rigorously test this assumption, as it only highlights anecdotal examples. Systematic benchmarking is really needed. This should include an estimate of predictive power and an estimate of false positive rates. Here's what I propose :
Figure 3k appears to show data that can serve a benchmarking purpose to a large extent, but the authors barely mention it. If I understood it correctly, figure 3k shows the RNA-seq allelic (im)balance of all genes with an allele-skewed microC contact with one of the probed SNPs. From these 236 SNP-gene pairs, 21 have significantly imbalanced expression (q-value < 0.05). So, from this basic analysis, it seems that the predictive power for functional interactions is roughly 21/236 = 9%. This number is worth highlighting in the manuscript.
However, this does not fully address the false-positive rate: genes may have significantly imbalanced expression by other causes, for example due to one of the many other SNPs that are in the same LD block. Thus, it needs to be estimated what this "background" rate of imbalanced expression is. For this, one will need a set of control genes that according to MCCv are *not* linked to an imbalanced SNP. For this control set, the imbalance rate also needs to be determined. Let's imagine this rate is 3%; then the false-discovery rate (incorrect assignment of the gene to the SNP) is about 3/9 = 33%. Note that this is different from the FDR on the y-axis of figure 3k, which only estimates the FDR in calling the gene expression imbalanced.
I strongly recommend that the authors include such a quantitative benchmarking calculation. What I propose above is just

one possibility (although it seems very feasible, as all data seems to be there); there may be other ways to approach it.

2a. At this stage the nano-scale patterns are not compelling to me. To the untrained eye, Figure 1d looks extremely noisy and it is not clear what kind of objective statistical analysis (if any) the authors applied to decide that the ref and alt alleles really differ. Given the apparently very high noise levels, the cartoon interpretations seem too speculative to me. There is also no evaluation of the reproducibility of this pattern: how do *independent biological* replicates compare? What did the other 44 tested SNPs look like? An extended "gallery" figure is needed to show all 45, including genomic coordinates (and the processed data should be made available). This will enable the reader to get a clear view on the kind of nano-patterns that can be obtained. A statistical test to evaluate allelic differences will be needed. Extended Figure 1h (MYB/TAL1) also looks very noisy, and reproducibility is not documented - even the nucleosome stripes seem virtually absent: how do the authors explain this?

2b. Conceptually, the starting point of figure 1 seems set up as a self-fulfilling prophecy. The 45 SNPs were chosen based on very strong allelic differences in ATAC-seq signals (Extended figure 1c). Given that the active allele typically has a nucleosome-free region, it is not so surprising that MCCv sees a difference in the fine cross-linking pattern. The real test is: if no prior selection was done based on ATAC-seq, would nano-scale patterns be discernable and useful to identify functional regulatory elements? Again, this would require an objective statistical scoring system to gauge the nano-patterns. It would also require rigorous benchmarking against ATAC-seq as method to detect allelic skews at nano-scale, because ATAC-seq is a much simpler assay than MCCv.

3. Figure 1g: FDR scores of peaks are calculated (by which method? please specify), but it is not clear whether the underlying statistical model takes reproducibility between independent biological replicates into account.

4. Figure 1i: It is not clear how many biological replicates this analysis is based on. If the error bars indicate the distribution of values of the 15 or 16 variants, then a t-test is not appropriate, because these variants are linked and hence not independent measurements (independence is a requirement for statistical testing). Instead, multiple biological replicates are needed, variant data from a single gene should be somehow combined/averaged per replicate, and then a t-test (or other test) across biological replicates should assess statistical significance. Same applies to figures 3i and 5h.

5. In figure 3k about half of the significant genes have a ref/alt skew, the other half a alt/ref skew. Does this match the skews in the nano-patterns (e.g. as shown in 3f,g)?

6. Please comment in the Discussion on the throughput limits of MCCv. What is the maximum number of SNPs that can be tested in parallel? In some experiments as many as 6 technical x 3 biological replicates were done. What does this mean in terms of labor, costs and scalability?

Minor points:

7. Legend Fig 1c: "Note that the active (alternate) alleles do not follow a nucleosomal pattern" - to my eye there is a clear nucleosomal pattern, it is just weaker. In the "nano-structure" heatmaps it may also be useful to add arrows or other marks to the structures that the reader should pay attention to; it took me several minutes to spot the (putative) differences.

8. Line 136: text: it is not clear whether the MYB/TAL1 observation was part of the same set of 45 SNPs, or a newly designed experiment altogether.

9. Extended Figure 2f does not speak for itself, and the legend is too brief. From this info I could not gather how the algorithm works.

10. Lines 211-213: "This approach revealed interactions between transcription factor binding sites on the reference allele": over-interpretation, at this point there is no evidence that these are TF binding sites.

11. Figure 3k [related to major point above] : the text and figure legend are a bit confusing: main text says "We extended this analysis to all genes with an MCCv contact", legend says "allelic expression of genes prioritised by MCC contact data". So is it MCC or MCCv? Please also spell out what "prioritised" means.

12. Throughout the manuscript some elements are referred to "enhancer" even though their enhancer function is not shown (yet). Better to refer to them as "putative enhancer" or similar wording.

13. The comparisons to conventional microC seem not entirely fair, as it depends largely on the relative sequencing depth. What if the microC samples were sequenced much deeper? If this is cost-prohibitive, then it would be good to explain what number of reads would be required to match the depth of MCCv.

14. It may be appropriate to cite and discuss PMID 39289357.

=======Reviewed by Bas van Steensel. Review assignment accepted: 17 Aug 2025; completed: 21 Aug 2025. It is my standard policy to sign *all* of my manuscript reviews, regardless of my comments and recommendations. All correspondence should go via the editor. PLEASE DO NOT REMOVE THIS DISCLOSURE. ============

Version 1:

Decision Letter:

27th Feb 2026

Dear James,

Your Article, entitled "Single-allele nanoscale mapping of regulatory variant function", has now been seen by the 3 original referees. You will see from their comments below that while they find your work improved, some important points are raised. We are interested in the possibility of publishing your study in Nature Genetics, but would like to consider your response to these concerns in the form of a revised manuscript before we make a final decision on publication.

We therefore invite you to revise your manuscript taking into account all reviewer comments. Please highlight all changes in the manuscript text file. At this stage we will need you to upload a copy of the manuscript in MS Word .docx or similar editable format.

We are committed to providing a fair and constructive peer-review process. Do not hesitate to contact me if there are specific requests from the reviewers that you believe are technically impossible or unlikely to yield a meaningful outcome.

When revising your manuscript:

*1) Include a “Response to referees” document detailing, point-by-point, how you addressed each referee comment. If no action was taken to address a point, you must provide a compelling argument. This response may be sent back to the referee(s) along with the revised manuscript.

*2) If you have not done so already please begin to revise your manuscript so that it conforms to our Article format instructions, available
here.
Refer also to any guidelines provided in this letter.

*3) Include a revised version of any required Reporting Summary: https://www.nature.com/documents/nr-reporting-summary.pdf
It will be available to referees (and, potentially, statisticians) to aid in their evaluation if the manuscript goes back for peer review.
A revised checklist is essential for re-review of the paper.

Please be aware of our guidelines on digital image standards.

EXTENDED DATA FIGURES

When re-submitting your manuscript, please ensure that any supplementary figures and tables that are crucial to the manuscript’s conclusions are converted into Extended Data figures and tables to increase visibility of these data. Extended Data figures and tables are online-only (present in the online PDF and full-text HTML versions of the paper), peer-reviewed display items that provide essential background to the article but are not included in the main article due to space constraints. A maximum of ten Extended Data display items (figures and tables) is permitted.

Please use the link below to submit your revised manuscript and related files:

Link Redacted

Note: This URL links to your confidential home page and associated information about manuscripts you may have submitted, or that you are reviewing for us. If you wish to forward this email to co-authors, please delete the link to your homepage.

We hope to receive your revised manuscript within eight weeks. If you cannot send it within this time, please let us know.

Please do not hesitate to contact me if you have any questions or would like to discuss these revisions further.

Nature Genetics is committed to improving transparency in authorship. As part of our efforts in this direction, we are now requesting that all authors identified as ‘corresponding author’ on published papers create and link their Open Researcher and Contributor Identifier (ORCID) with their account on the Manuscript Tracking System (MTS), prior to acceptance. ORCID helps the scientific community achieve unambiguous attribution of all scholarly contributions. You can create and link your ORCID from the home page of the MTS by clicking on ‘Modify my Springer Nature account’. For more information please visit please visit www.springernature.com/orcid.

We look forward to seeing the revised manuscript and thank you for the opportunity to review your work.

Sincerely,

Tiago

Tiago Faial, PhD
Chief Editor
Nature Genetics
https://orcid.org/0000-0003-0864-1200

Reviewers' Comments:

Reviewer #1 (Remarks to the Author):

The authors have made numerous improvements and addressed all of my comments.

Additional suggestions:
• For the new Fig 2h, could the authors add “closest gene” (and perhaps “2nd closest gene”) as a baseline for comparison?
• Re: Point 3, thanks for the explanation. Please add this explanation to Methods?

Reviewer #1 (Remarks on code availability):

The code repositories are now available and have some documentation. I did not attempt to run code.

Reviewer #2 (Remarks to the Author):

The authors have appropriately addressed all my comments.

Reviewer #2 (Remarks on code availability):

I did not review the code as it goes beyond my own expertise. Of note: this research group has an outstanding reputation when it comes to developing new analysis software for C-technologies.

Reviewer #3 (Remarks to the Author):

The revised manuscript has improved substantially, see specific points below. Whether the SESN3 part should be included is indeed up to the Editor, but the description of the MCCv method and data is now very condensed and not always easy to understand. More room for this key part would benefit the overall clarity.

One major remark: Title and Abstract very strongly state that this is a variant-to-function assay, and that target genes from 405 cis-regulatory elements are identified. This is rather misleading; strictly speaking, it is a variant-to-contact assay, and it seems that functional contacts are detected at rather low frequencies and with rather high FDR. I recommend that title and abstract clarify this by providing specific numbers; please see point 1 below.

Regarding previous major comments (numbering as in previous report):

1. I am pleased that a quantitative analysis was included figure 3k. But the main text glosses over this critical aspect: it is mentioned in only one sentence (lines 266-269) that does not clarify which proportion of tested elements can now be assigned to a target gene, at which estimated FDR. The figure legend is also rather cryptic, and the Methods do not seem to spell out how figure 3k was obtained. So, I am still struggling to understand: which proportion of enhancer variants with allelic imbalance of promoter contacts shows a significant RNA imbalance of the inferred target gene, at which FDR? This is key to the MCCv method and should be spelled out in the main text, to support the strong claims in Title and Abstract (see above).

If I understood it correctly, starting with 405 unique enhancer variants, 251 could be linked to a primary nominated gene (lines 207-209). But then the figure legend says that only 48 primary nominated genes for variants with evidence of MCCv changes were tested in figure 3k... Please clarify. Furthermore, figure 3k shows that only ~29% of these 48 show allele-specific expression, i.e., only 14 genes? And of the 25 secondary nominated genes ~24%, so 6 genes? Hence, starting with 405 enhancer variants, 20 genes could be linked to a target gene with an approximate FDR of ~25% (considering the random background of 7-9%)?? If I am mistaken with these calculations - then please spell out the correct calculations in the

main text. If I am correct, then also please spell out in the main text (and abstract) that MCCv finds that roughly 1 in 20 enhancer variants can be assigned functionally to a target gene, at an FDR of ~25%. These numbers are key!

Let me emphasize that I would not be disappointed if only 1 out of 20 enhancer variants has a detectable effect. This may be real biology, and such estimates are enormously useful for the community. An FDR of 25% is borderline acceptable, and perhaps the authors could indicate how they expect this to be improved in future versions of MCCv.

2a. Addition of the new statistical test is a major improvement. The "gallery" (now much expanded) of patterns is now available from Zenodo; it would be good to refer to this supplementary dataset in the main text.

2b. OK!

3. OK!

4. Even though the SNPs are covered by distinct RNA fragments, the fragments are still from the same gene and hence they are not independent (if the second half of the gene produces more RNA, then of course the first half does this too). Averaging multiple SNPs in a gene certainly adds robustness to the allele-specific RNA estimate (per replicate) but it cannot substitute for statistical analysis across multiple independent replicates. So, I still think the p-values are incorrect and overly optimistic...
BTW: Figure panel 1i now only has labels, no data.

5. I could not find the results mentioned in the response in the revised manuscript. Which figure?

6. I found part of this response in the Discussion; perhaps the details can be added to the Methods?

New minor points:
- Color key missing in figures 1c,d
- Figure 1i only has labels, data is missing
- Legend extended fig 1h erroneously says i instead of h
- line 125: text should state where the 42 maps can be found

=======Reviewed by Bas van Steensel. Review assignment accepted: 4 Feb 2026; completed: 14 Feb 2026. It is my standard policy to sign *all* my manuscript reviews, regardless of my comments and recommendations. All correspondence should go via the editor. PLEASE DO NOT REMOVE THIS DISCLOSURE. ============

Reviewer #3 (Remarks on code availability):

I could not access the code without registering on an Oxford University site. While I disclose my identity as a reviewer I do not think it is appropriate to require this. Code should be made available through Github, which is the standard platform.

Version 2:

Decision Letter:

Our ref: NG-A69906R1

16th Apr 2026

Dear James,

Thank you for submitting your revised manuscript entitled "Single-allele nanoscale mapping of regulatory variants" (NG-A69906R1). It has now been seen by reviewer #3 and their comments are below. Taking into consideration the reviewer's feedback, we'll be happy in principle to publish your manuscript in Nature Genetics, pending minor revisions to satisfy the referee's final points and to comply with our editorial and formatting guidelines.

We are now performing detailed checks on your paper and will send you a checklist detailing our editorial and formatting requirements soon. Please do not upload the final materials and make any revisions until you receive this additional information from us.

Thank you again for your interest in Nature Genetics. Please do not hesitate to contact me if you have any questions.

Congratulations!

Sincerely,

Tiago

Tiago Faial, PhD
Chief Editor
Nature Genetics
https://orcid.org/0000-0003-0864-1200

Reviewer #3 (Remarks to the Author):

0 (not numbered in previous exchange): Whether it is appropriate to call MCCv a variant-to-function assay: Consider the parallel with protein biology. A protein has a certain folding, which we usually refer to as the structure of the protein, not its function. Likewise, enhancer structure and chromatin folding are structural features, not function. But I leave it up to the editor to decide whether structural features can be called "function", and hence whether it is appropriate to call MCCv a variant-to-*function* assay, even though only a minority of identified variants show effects on RNA expression when tested.

Point 1. The explanation clarifies the calculations for this reviewer, but not really for the reader? It is a complex series of filters, admittedly too complex for the main text. But presently, the Results do not make it easy for the reader to understand this. The main text could perhaps highlight the key numbers in a simple short summary (e.g., we started with 405 variants; for various technical reasons ** dropped out; in the end we tested ** variants and found 45 variants a change in MCC contacts, of which 16 were confirmed by allele-specific RNA-seq). Note that I may mis-quote the numbers; I had to gather them from different parts of the Results text. A more detailed step-by-step explanation as in the rebuttal could then be provided as SuppInfo.
The argument that the FDR cannot be estimated weakens the case for the reliability of MCCv, to be honest. It seems best to adhere to the most conservative estimate, which is 33% based on the background measurements -- I do think that the reader should be made explicitly aware of this FDR estimate and how it is determined. Once again, I do not think that publication should depend on whether these numbers are high or low, but full clarity and transparency are important.

2-6 and minor points: OK.

=======Reviewed by Bas van Steensel. Review assignment accepted: 31 March 2026; completed: 3 April 2026. It is my standard policy to sign *all* my manuscript reviews, regardless of my comments and recommendations. All correspondence should go via the editor. PLEASE DO NOT REMOVE THIS DISCLOSURE. ============

Open Access This Peer Review File is licensed under a Creative Commons Attribution 4.0 International License, which permits use, sharing, adaptation, distribution and reproduction in any medium or format, as long as you give appropriate credit to the original author(s) and the source, provide a link to the Creative Commons license, and indicate if changes were made.
In cases where reviewers are anonymous, credit should be given to 'Anonymous Referee' and the source.
The images or other third party material in this Peer Review File are included in the article's Creative Commons license, unless indicated otherwise in a credit line to the material. If material is not included in the article's Creative Commons license and your intended use is not permitted by statutory regulation or exceeds the permitted use, you will need to obtain

permission directly from the copyright holder.
To view a copy of this license, visit https://creativecommons.org/licenses/by/4.0/

Thank you for reviewing our manuscript, '*Single-allele nanoscale mapping of regulatory variant function*". We very much appreciate the time you have taken to review our study and your constructive comments, which we believe have resulted in substantial improvement of our study. We have added several new experiments and analyses, which strengthen the study and we have revised the manuscript to improve clarity.

The major additional experiments and analyses are as follows:

1. **All code has been made available**, in response to the request from Reviewer 1. The code is now downloadable from the Nature manuscript central. The GitHub repository is now publicly available and our commercially licenced code is also available for academic use through the University of Oxford Software store.
2. **We have improved the statistical analysis throughout the manuscript including development of a customised statistical package (basepairC)**, in response to comments from Reviewers 1&3. We have been developing a statistical package for comparison of base pair resolution heatmaps, which is being prepared for independent publication. This has been used to analyse the single allele nanoscale heatmaps and this confirms statistically significant changes in the patterns between active and inactive alleles.
3. **We have generated MCCv data from an additional 176 sites with different chromatin states,** in response to comments from Reviewer 1 and Reviewer 3. This includes analysis of inactive and active chromatin states including a large number of control SNPs without identified function. This shows that the MCCv contacts have minimal changes at control enhancer SNPs (which are likely non-functional) compared to disease associated SNPs. In addition, we find that disease associated SNPs that are located outside of enhancers do not alter MCCv contacts.
4. **We have included analysis of enrichment of the functional variants and genes we have identified with background SNPs**, in response to comments from Reviewers 1&3. This included analysis of all common variants found in dbSNP compared with the variants associated with IMIDs.
5. **We have improved Figure 6 to clarify the mechanism of action of *SESN3***, in response to comments from Reviewer 2. We believe that this has resulted in improved clarity of these findings.
6. **We have undertaken thorough benchmarking of MCCv with allele-specific expression data**, in response to comments from Reviewer 3. This shows that SNPs which result in a change in MCCv signal are significantly more likely to be linked to genes with allelic imbalance in gene expression.
7. **We have undertaken further experimental work to improve the analysis of the single allele nanoscale maps**, in response to comments from Reviewer 3. This included additional experimental work to define the 2.5 kb region around the variants at base pair resolution, to aid interpretation of the MCCv heat maps.
8. **We have compared conventional footprinting with ATAC-seq and MCC, with the nanoscale structures**, in response to comments from Reviewer 3. This included developing a targeted capture method for ATAC-seq, which shows that even with deep coverage (~1000x) limited allele specific information can be obtained.

Point by point response to reviewer's comments (in blue)

Reviewer 1

This study by Hamley et al. reports the extension of Micro Capture-C to study noncoding variants ("MCCv") and its application to study variants associated with inflammatory diseases. The authors show that analyzing allele-specific 3D contacts (through hybrid capture of genomic regions containing variants) can identify effects on noncoding variants both at the 'nanoscale' (nearby nucleosomes, ~500bp) and at longer range (kilobases to megabases). The authors apply MCCv to ~400 variants associated with inflammatory diseases that overlap enhancers in activated human T cells, and illustrate numerous examples where the 3D contact links an element to a gene and where the allele-specific information shows an association with the variant. One such locus is the SESN3 locus, where a risk variant creates a new CTCF motif that blocks a super-enhancer from contacting the SENS3 promoter. Knocking out Sens3 in mouse T cells affects disease phenotypes in the EAE mouse model of autoimmunity, and the authors conduct experiments to show that this may be mediated by changes in mTOR signaling and tryptophan sensing.

Altogether this is an exciting study. The MCC data looks very clean and have some striking effects from allele-specific analysis. The results for the SENS3 locus appear to reveal some novel function of this gene in autoimmunity, and illustrate what can be learned by applying MCCv to study GWAS variants. Overall, well done!

We thank the reviewer for their very encouraging comments regarding our study.

Before publication, however, additional analyses should be conducted and major improvements made to the description and/or application of statistical and computational methods, and to the analysis of GWAS variants:

1. The GitHub link in the 'Code Availability' section is not publicly accessible. It should be made so for review, and may have helped to answer some of the following questions. That said, the Methods should also be more detailed and not rely on the Code to provide all of these answers.

We apologise that the code was not available for review. We are very happy to share the code with the reviewer and have uploaded all of the code to the Nature Manuscript Central as a supplemental file. The central engine of the MCC code has been licenced commercially and is available through the Oxford University Software Store but this would compromise reviewer anonymity. We have therefore included this component as supplementary material in the submission for review purposes. The SNP prioritisation pipeline and basepairC package, which are not subject to licensing restrictions, are now publicly available at the following GitHub repositories:

- https://github.com/Davies-Genomics-and-Genome-Editing-Lab/snp_prioritization_pipeline
- https://github.com/jamesdalg/basepairC

In addition, we have expanded the Methods section to provide a more detailed description of the MCC workflow and analysis steps, so that key aspects of the approach are fully described independently of the code.

1a. A central point in this paper is comparing 3D contacts between two alleles in the same cell population. However, I did not find any methods description of how the authors are quantifying changes in contacts, e.g. between two alleles or between two viewpoints. Fig. 1g appears to present the results of a 'paired t-test', but there is no description that I can find that explains what is being quantified or what is being paired. An 'FDR correction' is cited, but the tool cited appears to have various parameters and the methods do not describe what parameters were used.

We thank the reviewer for highlighting this and have amended the methods section to increase clarity of figures relating to quantification of MCCv count differences between alleles (e.g. Figure 1g). This can be found in the "MCCv peak quantification comparison" section.

In summary, the following steps were used in this analysis:

1. Peaks were called with a custom MCC specific peak calling algorithm Lotron MCC (available for academics through the OU software store; details of the algorithm are available in the methods section and software documentation).
2. Significant peaks are filtered based on distance (> 1.5 kb from the capture site, > 50 bp in width, and within 2 Mb of the capture point).
3. The number of unique PCR duplicate filtered ligation junctions over each peak was quantified for the corresponding viewpoint in both the reference and the alternate allele.
4. These counts were normalised to the total number of *cis*-unique ligation junctions from the corresponding viewpoint in the reference and alternate allele.
5. At a minimum we have 6 replicates (1 donor being heterozygous for Figure 3 or 1 cell line e.g. Jurkat for Figure 1), thus we have a 6 x 6 paired comparison which we compare using a paired Student's T test. A Kolmogorov-Smirnov test was used to show that the data was normally distributed between replicates.
6. To correct for multiple testing the R package fdrtools was used (https://doi.org/10.1093/bioinformatics/btn209). This is a widely used package (> 600 citations), which estimates the local false discovery rates from the empirical distribution of *p*-values within the data.

1b. Beyond Fig. 1g, there do not appear to be many statistics reported regarding the magnitude or significance of differences for specific contacts (e.g., Fig 2c, Fig. 3d,e, etc.). In many cases the magnitude of the difference seems large and likely robust. Nonetheless this should be quantified.

We thank the reviewer for this comment and have now undertaken further analysis and have added additional data to the paper (**Extended Data Figure 3g** (rs10950648), **Extended Data Figure 3h** (rs9526003), and **Extended Data Figure 4h** (rs4409785)).

For the allele specific data, we now show quantification of the junctions within MCC peaks for individual SNPs and formally test their statistical significance as described above and in the Methods section “MCCv peak quantification comparison”.

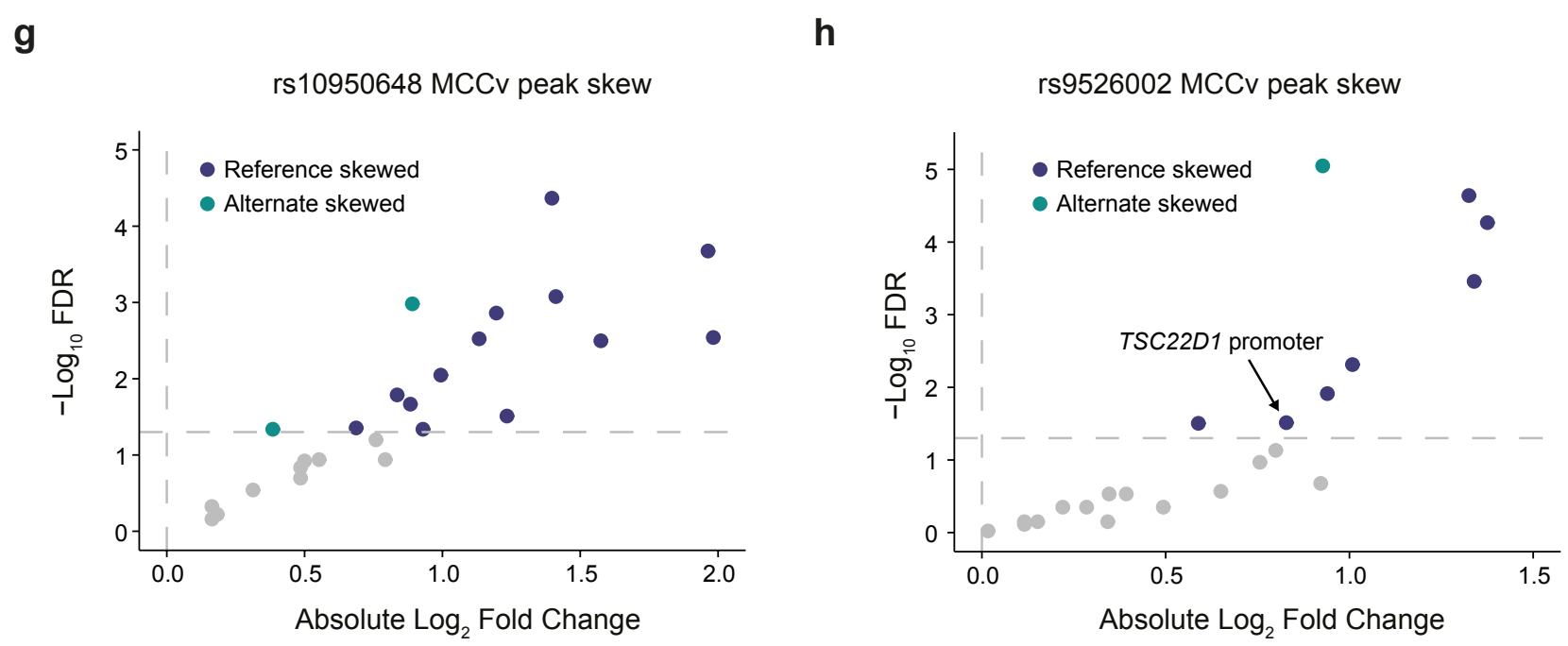

Extended Data Fig. 3g and h. g, Absolute $\log_2$ fold change of junction counts between reference and alternate alleles at called Lotron MCC peak for rs10950648 (n = 12, paired Student’s T-test, two-sided, local FDR estimate with fdrtools). **h,** as in **g** but for rs9526002. The *TSC22D1* promoter peak is highlighted as being significant.

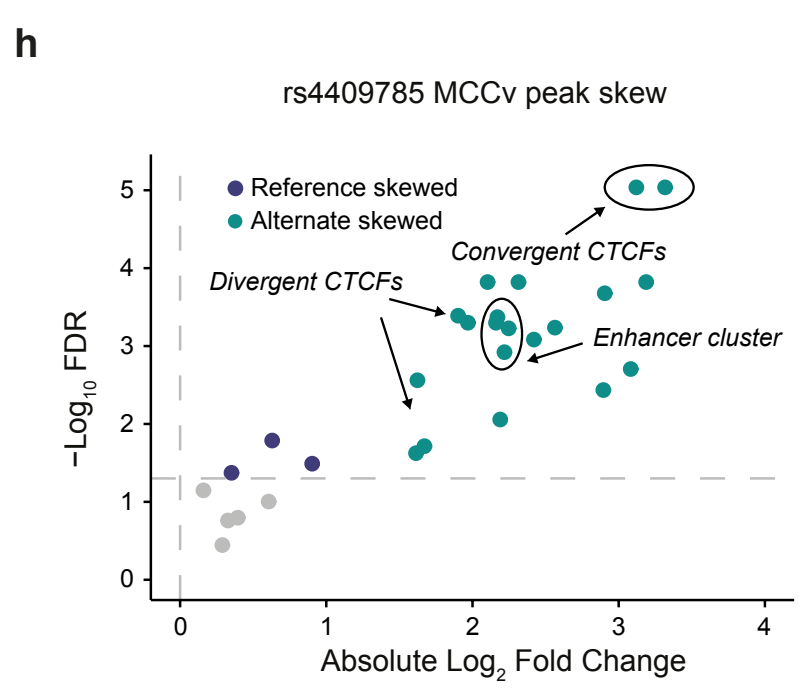

Extended Data Fig. 4h Absolute $\log_2$ fold change of junction counts between reference and alternate alleles at called Lotron MCC peak for rs4409785 (n = 6, paired Student’s T-test, two-sided, local FDR estimate with fdrtools). Key CTCF peaks as well as the peaks belonging to the *SESN3* super-enhancer are highlighted as being significant.

For Figure 2c, which shows bulk (non-allele-specific) MCC data, we quantified normalized junction counts at three gene promoters for each variant. These analyses are now shown in **Figure 2d**. This quantification confirms that rs75084216 (eczema-associated) preferentially contacts *FASL*, whereas other disease-associated variants in the locus do not contact FASL or the nearest gene *TNFS18*, but instead form long-range contacts with *TNFS4*.

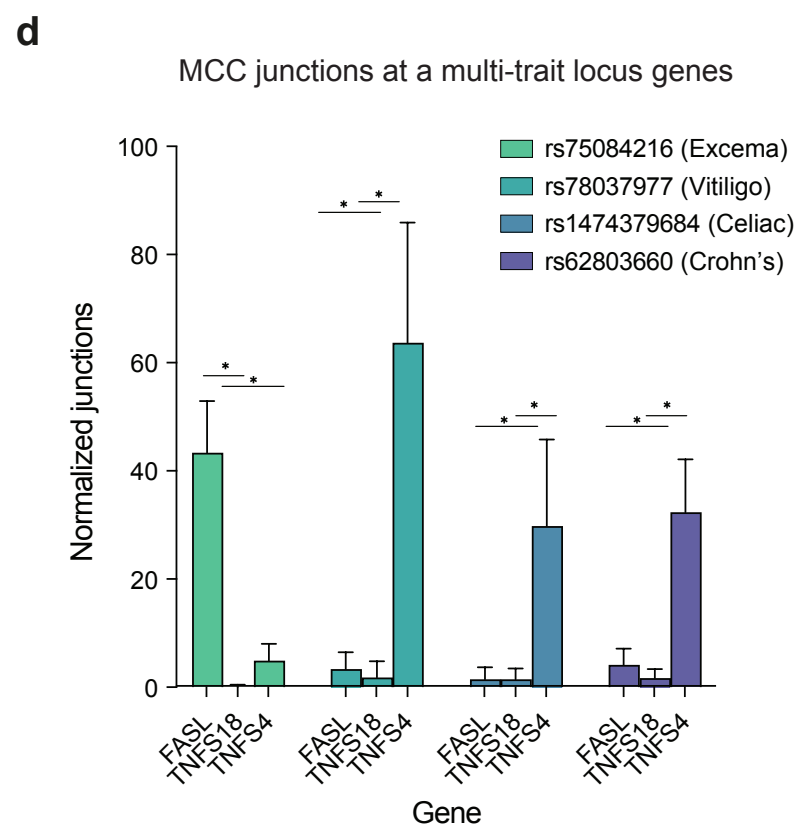

Fig. 2d. Quantification of normalized MCC junctions at the 3 genes in the multi-trait locus for all 4 disease associated variants (n = 18, mean ± s.e.m., paired Student's T test, two-sided, two-stage step-up method for multiple test correction, * corresponds to $p \leq 1 \times 10^{-6}$).

Together, these additions provide quantitative support for the contact differences highlighted in Figures 2c, 3d, 3e and related panels.

1c. The GWAS variant prioritization involves selecting GWAS signals that include variants that overlap enhancers or CTCF sites. Again, details for this analysis are very light. Extrapolating from Fig 2b and the Methods, it seems that the approach for selection was to look at GWAS loci where all variants within r^2 of 0.7 overlap enhancers or CTCF sites (and not have variants overlapping coding variants or splice sites), and then select those variants that overlap. How are CTCF sites and enhancers defined?

We thank the reviewer for this comment and have substantially expanded the description of this analysis in the Methods (section "GWAS Variant Prioritization") and in the Extended Data Figure 2 figure legend.

For each GWAS signal we defined haplotypes by imputing variants in linkage disequilibrium with the lead variant with an r^2 of 0.7 using LD link (https://doi.org/10.1093/bioinformatics/btv402). All variants within each haplotype were annotated as either: coding, splicing, 3'UTR, promoter, enhancer, or CTCF based on the following criteria:

I. Coding – non-synonymous variants labelled with ANNOVAR (https://doi.org/10.1093/nar/gkq603) run with default parameters on the hg38 genome build.
II. Splicing – variants were scored with spliceAI (https://doi.org/10.1016/j.cell.2018.12.015) and those variants with a delta score > 0.2 (donor or acceptor) were annotated as splicing variants.
III. 3'UTR – ANNOVAR defined 3'UTR variants were intersected with a list of known miRNA binding sites (https://doi.org/10.1093/nar/gkz757).
IV. Promoter/Enhancer – variants were intersected with cell type specific ATAC, which had peaks called using Lanceotron (https://doi.org/10.1093/bioinformatics/btac525) and filtered for high quality peaks (peak score > 0.5). The data were filtered for high quality intersections

(within 100bp of the peak summit and signal coverage at variant position >= average signal coverage of the peak). Promoter variants were annotated with CAGE-seq intersection for transcription start site identification. Distal ATAC-seq peaks lacking CAGE signal were classified as putative enhancers.

V. CTCF – intersection with deeply sequenced CTCF ChIP-seq data from 50 healthy donors. Peaks were called using Lanceotron and filtered for high quality peaks (peak score > 0.5). Intersections were filtered as above.

For haplotypes with enhancer variants, viewpoints were designed to the enhancer variant and captured in activated $CD4^+$ T cells in 3 healthy donors. This included haplotypes for which there was a secondary molecular mechanism (e.g. coding or splicing), beyond the potential enhancer mechanism.

1d. 'We used a variant-promoter contact frequency ranking to nominate 251 unique causal genes' (Line 185) — What does that mean, exactly? Take the single promoter per locus with the strongest contact frequency?

This is correct - we selected a single gene promoter per captured SNP based on the strongest variant–promoter contact frequency. We have modified the text and associated figure to improve clarity. Furthermore, we have added a methods section ("Nomination of causal genes") on our strategy to assign potential causal genes as well as to detail the downstream validation analysis. For each SNP, we initially called MCCv peaks using Lotron MCC, which is a tool that we specifically developed for peak calling MCC data. We then identified all of the promoter peaks for each variant using CAGE-seq defined transcriptional start sites. We quantified the *cis*-normalised ligation junction counts at each promoter peak. For a given SNP, its associated promoter peaks were then ranked and the gene promoter with the highest number of junctions was identified (primary nominated gene).

2. The authors should conduct additional analysis of the variants they selected for study, the variants that had allelic effects in MCCv, and the 251 genes prioritized. In general, for these results to be actionable for downstream experiments or pre-clinical investigation, it is critical to estimate how enriched they are versus some appropriate background. Because of confounding from linkage disequilibrium, the fact that contact != regulation, and the multi-way relationships between enhancers and genes, it could be very common that variants have allele-specific effects on 3D contacts and link to genes that are not causally related to disease.

2a. For the selected variants — authors should report the enrichment of GWAS variants by their definition (e.g., r^2 > 0.7) in each category (enhancers, CTCF sites, coding, splice site), versus e.g. the set of all common variants. E.g. Enrichment = (Fraction of considered GWAS variants that overlap enhancers in T cells) / (Fraction of all common variants that overlap enhancers in T cells).

We thank the reviewer for this insightful comment and agree that utilising a background set of common SNPs will add more context to the output of the SNP prioritisation pipeline. To address this, we ran the dbSNP151 common dataset (a total of around 15

million common variants) through the SNP prioritisation pipeline to get “background scores”. We saw the greatest enrichment in enhancer variants, as well as significant enrichment in promoter and splicing variants. 3’UTR variants were significantly anti-enriched. These new analysis have been added as part of **Extended Data Figure 2e**.

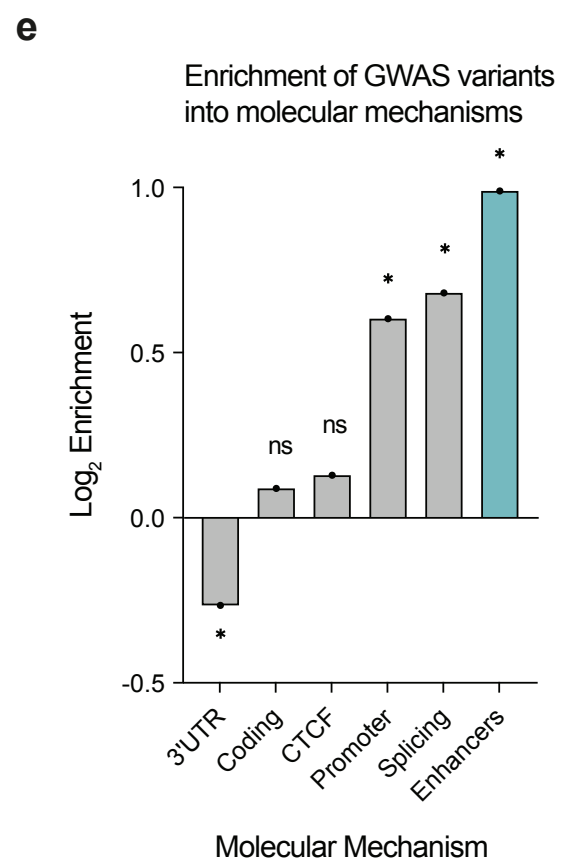

Extended Data Fig. 2e. Enrichment scores of IMID-associated SNPs for each molecular mechanism tested in the SNP prioritisation pipeline (n = 124,000) compared to dbSNP151 common variants across the genome (n = 15,000,000). Enrichment scores were calculated as: fraction of GWAS variants that can be explained by the tested molecular mechanism over fraction of total common variants explained by tested molecular mechanism (Chi-squared test, 3’UTR (p = 0.001), coding (p = 0.145), CTCF (p = 0.203), promoter ($p = 3.68 \times 10^{-10}$), splicing ($p = 7.97 \times 10^{-13}$, enhancer (4.67×10^{-53})).

2b. For the variants that have allelic effects — Are there control sets of variants that were selected, such that a similar calculation could be performed? Alternatively, if there is fine-mapping available for any of the variants studied, you could show the ‘Fraction of high-probability variants that have allelic effects’ vs ‘Fraction of low-probability variants that have allelic effects’.

We thank the reviewer for this helpful comment and have undertaken further experimental work including generating MCCv data from an additional 158 SNP based viewpoints (allowing quantification of an additional 1530 associated MCCv peaks) to address the reviewer’s comment.

In our initial allele specific MCCv analysis, there was at least one statistically significant change in peak junction count for 54.6% of variants.

The expanded dataset allowed us to implement several complementary control strategies that directly address the reviewer’s question:

1. **Cell type controls**. Our initial MCCv dataset included 14 heterozygous viewpoints from monocyte rather than T cell enhancer variants from IMID GWAS signals. Only 1 of these 14 SNPs (7.1%) had a statistically significant difference in peak junction counts between alleles, a 7.7-fold reduction compared to the captured disease-associated enhancer variants in the correct cell type.
2. **Haplotype controls**. To control for other SNPs within the GWAS haplotype which do not intersect with open chromatin we performed an additional MCCv

experiment at 82 sites. Significant changes in the allele specific MCCv profiles were only seen at 6 of 82 (7.3%), a 7.5-fold reduction compared to the captured disease-associated enhancer variants in the haplotype.

3. **Enhancer controls**. Finally, we generated additional MCCv data at 76 non-disease associated SNPs, which intersect with T cell enhancers identified by ATAC-seq. Here we saw differences in the allele-specific MCCv peaks from 25% of variants (19 of 76), a 2.2-fold reduction compared to the captured disease-associated enhancer variants.

All of these different control approaches demonstrate that disease associated enhancer variants have greater changes in MCCv peak junction counts between alleles compared to background SNPs. This analysis has been incorporated in the manuscript as part of **Figure 3c** and details of the heterozygous control sites included has been added to the Methods section "GWAS variant prioritization".

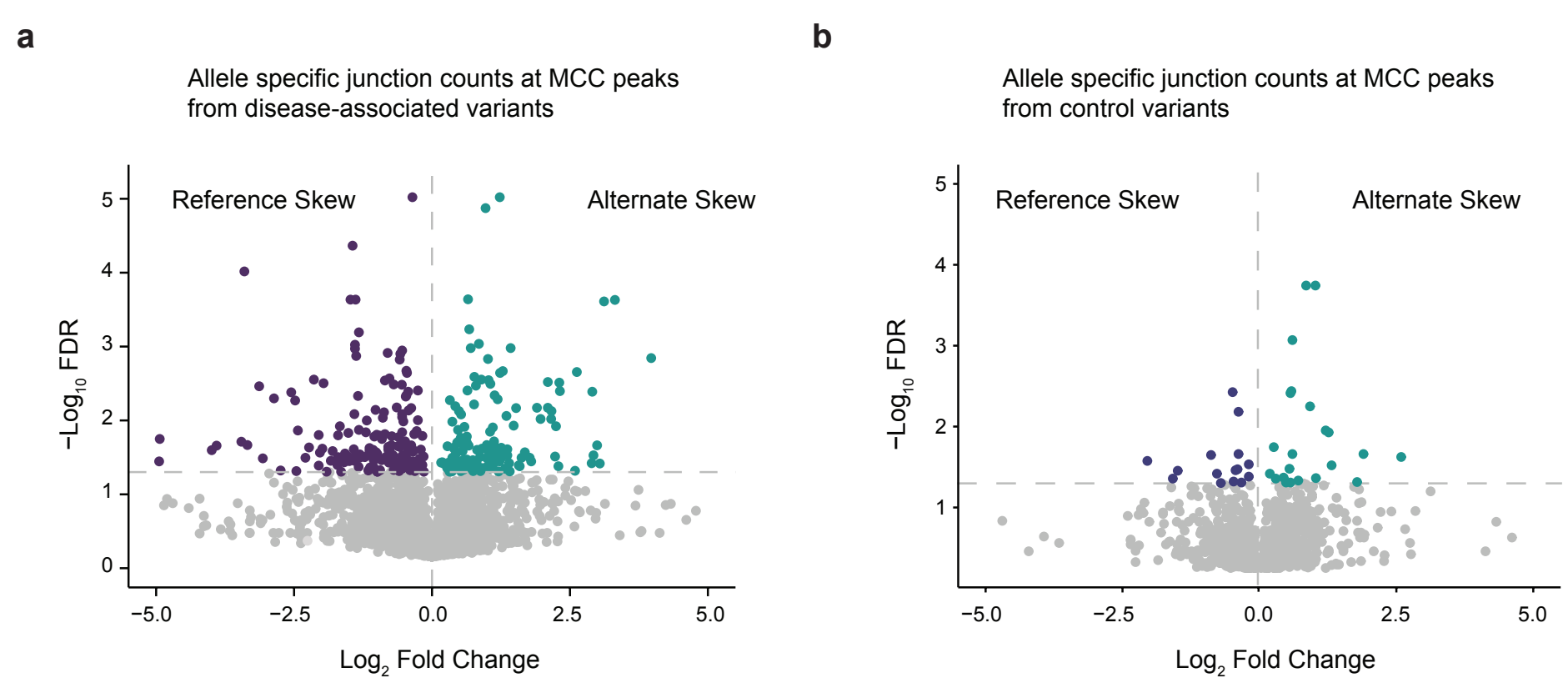

Figure R1 a, Volcano plot summarising total MCCv data for the IMID-associated variants. Junction counts at peaks called by Lotron MCC were compared between reference and alternate alleles (n = 6 or 12 or 18 per datapoint depending on number of donors with heterozygosity, Student's t-test, two-sided, paired, Student's t-test, two-sided, paired, fdr correction (local fdr estimation (fdrtools)). **b,** Volcano plot summarising MCCv data for three classes of controls (cell type, haplotype and enhancer controls). Cell type controls are SNPs which intersect with monocyte open chromatin but not T cell open chromatin; haplotype controls are additional variants within the GWAS haplotype; and enhancer controls are non-disease associated variants which intersect T cell open chromatin. Peaks called by Lotron MCC were compared between reference and alternate MCCv junction counts for all peaks for all SNPs with allelic data (n = 6 or 12 per datapoint depending on number of donors with heterozygosity, Student's t-test, two-sided, paired, fdr correction (local fdr estimation (fdrtools)).

c

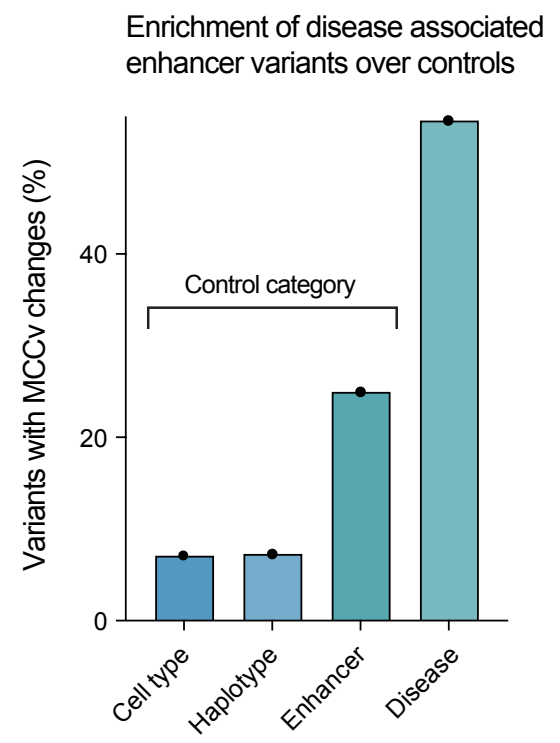

Fig. 3c. Percentage of variants with ≥ 1 statistically significant MCCv peak change between alleles in a variety of control variant categories (Cell type, Haplotype, Enhancer) as well as for the disease-associated T cell regulatory variants (Disease) (Chi-squared test, disease variants n = 227 – enhancer enrichment 2.2, p = 0.000014, n = 76; haplotype enrichment 7.5, $p = 2.73 \times 10^{-13}$, n = 82; cell type enrichment 7.7, p = 0.0015, n = 14). Cell-type controls are IMID-variants in monocyte enhancers; haplotype controls are other SNPs in the haplotype that do not intersect $CD4^{+}$ T cell open chromatin; enhancer controls are variants which intersect $CD4^{+}$ T cell open chromatin but are not associated with IMIDs.

2c. The 251 'unique causal genes' should be similarly evaluated. E.g. Fig. 2i reports the number that Approved Drug targets, but not the enrichment. For example, Enrichment here could be calculated as 'Fraction of prioritized genes that are Drug targets' / 'Fraction of all candidate genes in these loci ... e.g. all genes within 1Mb, perhaps... that are Drug targets'. It would also be useful to compare these to predictions from other prediction methods, such as OpenTargets L2G and PoPS (Weeks et al. Nature Genetics).

We agree with the reviewer that enrichment analysis provides a more informative assessment of gene prioritisation than raw counts and have thus have replaced Extended Data Figure 2i (raw counts) with Figure 2g (enrichment over background gene set). Enrichment was calculated as the fraction of prioritised genes belonging to a given annotation category divided by the fraction of all candidate genes within ±1 Mb of the corresponding SNPs belonging to that category. We have added details of this analysis to the Methods section "Nomination of causal genes". Using this framework, we evaluated enrichment for drug targets, genes linked to approved drugs, and genes with a known coding mutation linked to primary immunodeficiency.

This revised analysis demonstrates that MCCv primary nominated genes (previously gene 1) are significantly enriched for all three categories relative to the local genomic background, whereas secondary nominated genes (gene 2) show weaker or no enrichment. These results support the conclusion that ranking genes by variant–promoter contact frequency preferentially identifies more plausible causal genes.

To further increase the confidence of our prioritised set, we also looked at enrichment for essentiality using the Depmap database and found that our primary nominated gene list was anti-correlated with essentiality whilst the secondary nominated genes were partially enriched. This suggests that MCCv is not simply selecting broadly essential or housekeeping genes but instead prioritises genes with more specific roles in immune biology.

h

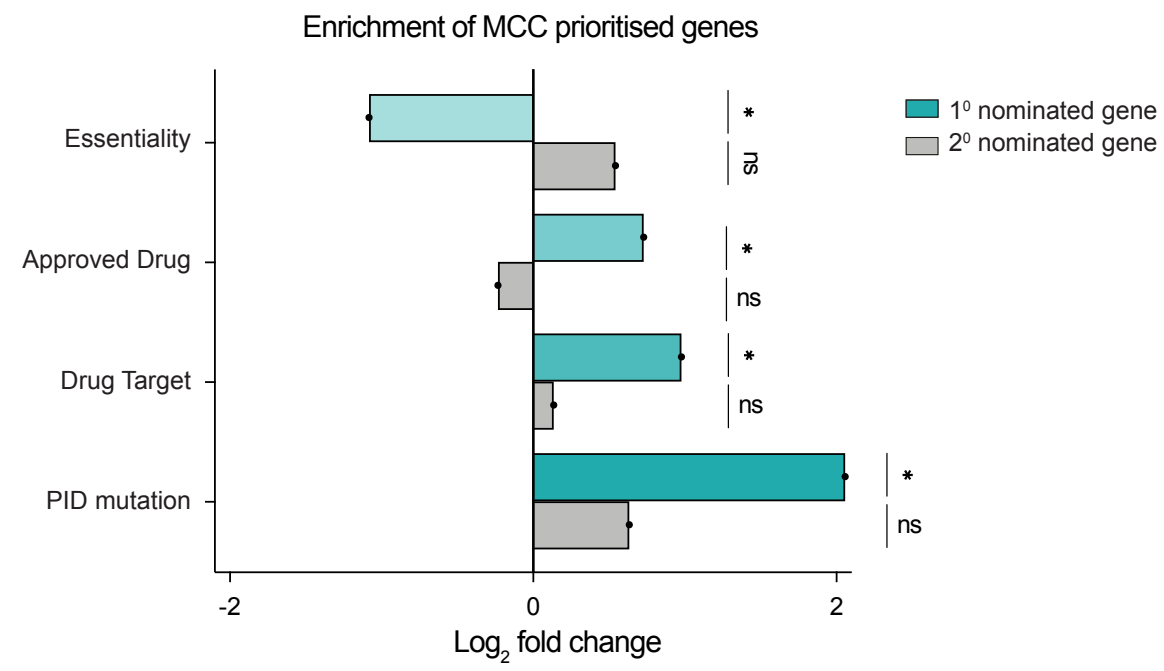

Fig. 2h. Enrichment of MCC prioritised genes (rank 1 genes) into different gene categories compared to background (all genes +- 1 Mb from captured variants). Essentiality was assigned based on Depmap essentiality screening. Drug target was any gene with a drug in a clinical trial, whilst approved drugs were assigned for genes with a drug in phase 4 clinical trials. Primary immunodeficiency mutation was assigned using a curated database of PID associations (sourced from Genomics England) (Essentiality – $p = 0.0173$ (gene 1), $p = 0.136$ (gene 2), Approved drug – $p = 0.0379$ (gene 1), $p = 0.840$ (gene 2), Drug target – $p = 2.65 \times 10^{-5}$ (gene 1), $p = 0.849$ (gene 2), PID mutation – $p = 2.14E-21$ (gene 1), $p = 0.232$ (gene 2), Chi-squared test).

For comparison with existing gene prioritisation approaches, we analysed prioritised genes from OpenTargets L2G, as suggested by the reviewer. When considering the single highest-scoring L2G gene per variant–trait pair, MCCv and L2G prioritised the same gene in 56% of cases. This concordance increased to 67% when considering the top two L2G-ranked genes. This has been highlighted in the manuscript text. PoPS prioritisation was not included here due to the requirement for full summary statistics to run the pipeline, which were not available for all the included GWAS studies.

3. Could the authors explain how exactly we should interpret the allele-specific data, in light of any statistical normalization or differences in MNase accessibility between alleles? For example, if allele A is more accessible to MNase and therefore creates more total contacts than allele B, but has the identical profile how is that represented/interpreted in the log-fold-change allelic analysis?

We thank the reviewer for raising this important point. We have previously undertaken extensive analysis of the potential bias in MCC (see Hua et al., Nature 2021, https://doi.org/10.1038/s41586-021-03639-4 Extended Data Fig. 2) and found that this is a relatively minor problem. This is in part because we undertake very careful titration of the MNase concentration to ensure that chromatin is digested so that the mononucleosomal band (~200 bp) predominates.
In addition, all contact profiles including the allele-specific contact profiles are normalised based on the total number of *cis*-unique ligation junctions per viewpoint/SNP. On average, variant alleles have 20% differences in total *cis*-unique ligation junctions and thus our average normalisation factor is within this range. If the difference in junction count exceeds this we would investigate for technical problems in the experiment and either exclude the data from analysis (if there were poor oligonucleotide capture, for example) or we would down sample the data. This normalisation step is intended to control for global differences in sequencing depth and overall ligation junction capture between alleles, including differences driven by MNase accessibility. As a result, we

believe the log-fold-change values reported reflect relative differences in the distribution of contacts across peaks, rather than absolute differences in the total number of contacts generated by each allele. Statistically significant allele-specific effects arise primarily when there is a redistribution of contacts, such that specific peaks show a disproportionate gain or loss of ligation junctions relative to the total *cis*-contact signal for that allele.

Other comments and questions:

• '10 million human genomes have been sequenced' (page 3). Citation?

We thank the reviewer for pointing this out. We have added references to key sequencing databases (UK biobank, All of US, Genomics England etc.) and have adjusted the wording to increase the clarity.

• 'more enhancer activity' (page 5). What are you defining as enhancer activity, here? 'more accessibility' might be more precise here, if I understand correctly

The reviewer is correct, 'more accessibility' is a more precise description of how we selected the variants. To further increase confidence that these variants were indeed having an effect on enhancer activity, we have now included allelic skew in H3K27ac ChIP-seq at the same sites and added these new data to **Extended Fig 1b**. Although this further analysis has strengthened the evidence that these are indeed enhancer variants, we agree that the term 'more accessibility' is more accurate and we have reworded the manuscript in line with the reviewer's suggestion.

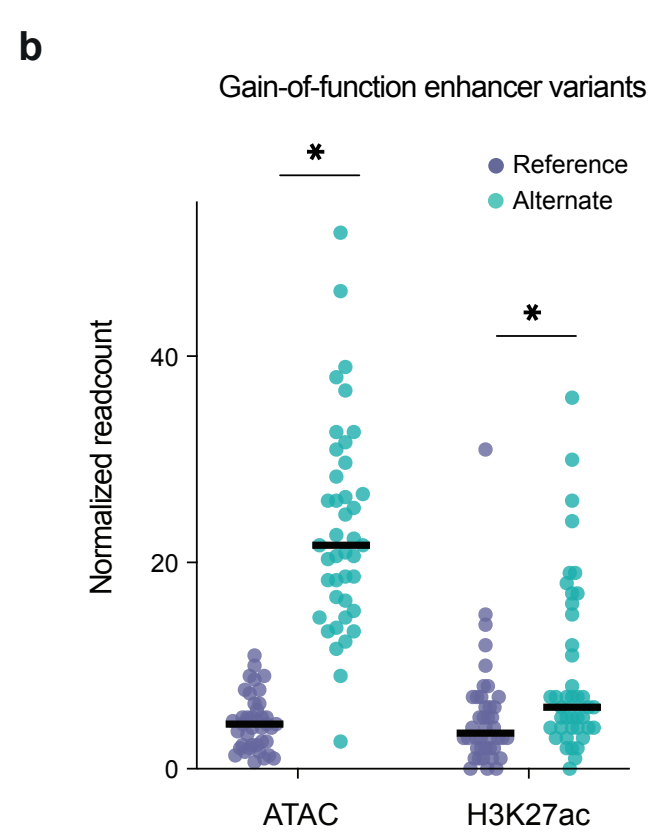

Extended Data Fig. 1b, Skew data for ATAC and H3K27ac ChIP sequencing at the selected 42 enhancer elements demonstrating potential gain-of-function in terms of activity of enhancer (n = 3 (ATAC), n = 3 (H3K27ac), per variant, Reference vs. Alternate comparison – Wilcoxon sign-rank test, two-sided, two-stage step-up procedure for multiple test correction, q < 0.000001 (ATAC) and q = 0.000029 (H3K27ac)).

• "minimizes experimental noise" (line 145) — I'm not sure I caught what this is referring to

We thank the reviewer for this comment and have clarified this point in the manuscript. By "minimizes experimental noise," we refer to the reduction in variability obtained by

comparing alleles within the same sequencing library, rather than comparing data across independently genotyped donors.

• Capture oligos were designed either centered around the SNP or the peak of the regulatory element (Methods, page 17). Can you provide some rationale for why to design one way or another in the future?

We apologise as this was not clear in the methods. When performing MCCv, the 120-bp oligos are always designed with the SNP in the central position if possible. If the oligo cannot be designed in such a way (due to spurious alignment to other genomic regions, sequence repeats, or GC content), we check if an oligo can be designed with the SNP in any other position (120 design iterations). If no SNP-based oligo can be designed, then oligos will be designed to be as close to the summit of the regulatory element (as defined by ATAC-sequencing peaks) as possible. We have amended the methods section "Capture oligonucleotide design" to make this clearer.

• Fig. 2f — define 'Gene Ratio'?

We have amended the figure legend to include a more detailed description of GeneRatio from the output of the enrichGO() function from the clusterProfiler (DOI: 10.1016/j.xinn.2021.100141) R package. Here it is defined as the number of genes in the input list that are annotated to the corresponding term (function) / the number of genes in the input list.

Reviewer #2 (Remarks to the Author):

In their paper, Hamley et al. describe a technology called MCCv that was developed to exploit high-res 3D genome conformation measurements to help identify functional genetic variants (e.g. SNPs) in regulatory elements. The latter remains a major challenge in the field, and hence this is an important pursuit.

The authors have a long-standing interest in this topic, which started with the Hughes et al. 2014 Capture-C paper in which they also used improved C-technology to link SNPs to putative target genes. Now they take advantage of their more recently developed MCC technique (PMID: 34108683). Major advance of MCC is its basepair resolution, which allows for the generation of allele-specific maps and combined phasing of other omics data.

The overall approach of using (high-res) 3D genome conformation to link disease-associated SNPs to candidate causal genes is very valid but not necessarily very novel. However, the authors do take this approach to a next level by tailoring their recently developed MCC technology to the 'SNP-gene assigning problem'.

Technically, the work is very impressive and robust, leaving this reviewer with little doubt about the added value of the MCCv platform to the field. The paper contains mostly anecdotal evidence from selected loci, but this is fine considering the topic and the aim

to showcase the power of the technology. The authors finish with a more in-depth analysis of the function of a GWAS/MCCv hit (SESN3), which in my opinion is a bit weaker than the rest of the manuscript.

We thank the reviewer for their positive comments on the work.

Comments:

- Although MCCv is a very valuable addition for post-GWAS follow-up studies, the authors to my opinion often overstate the novelty of the methodology itself. In the end, MCCv is MCC with a few modifications, and I think it doesn't need to be 'hyped' all the time in that way.

We thank the reviewer for this comment. This was not our intention and we have reviewed manuscript to remove overstatements with regards to the technology.

- The authors generate SESN3-KO primary CD4+ T cells, but then seem to switch back to Jurkat cells for the biochemistry. Fair enough for showing the protein-protein interactions, but this functional follow-up part would be much more convincing if the authors could directly show altered responses of these SESN3-KO T cells to relevant activation signals.

We apologise that this wasn't clear in the manuscript. Only the biochemical studies using co-IP of SESN3 and GATOR2 were done in Jurkat cells because this required engineering of a 3xFLAG tag into the endogenous SESN3 gene. All of the functional analysis of the effects of *SESN3* loss was performed in primary human T cells. We undertook analysis of *SESN3*-KO in primary human CD3+ T cells and show that these have abnormal mTOR pathway signalling compared to WT (AAVS1 edited) human T cells, specifically in conditions with low amino acid levels (**Figure 6i**). We have now clarified this in the figure legend and methods.

- I find the model drawn in Fig.6k to be confusing. The GWAS variant leads to a moderate reduction in SESN3 – that's clear – but how does that then lead to excessive/chronic immune activation? Because that's the expected outcome of the variant given its risk-increasing association with auto-immune diseases.

We thank the reviewer for this comment and apologise that Figure 6k was not sufficiently clear. We agree that the causal logic linking reduced *SESN3* expression to increased immune activation requires clearer explanation and we have improved the figure and accompanying text in the discussion.

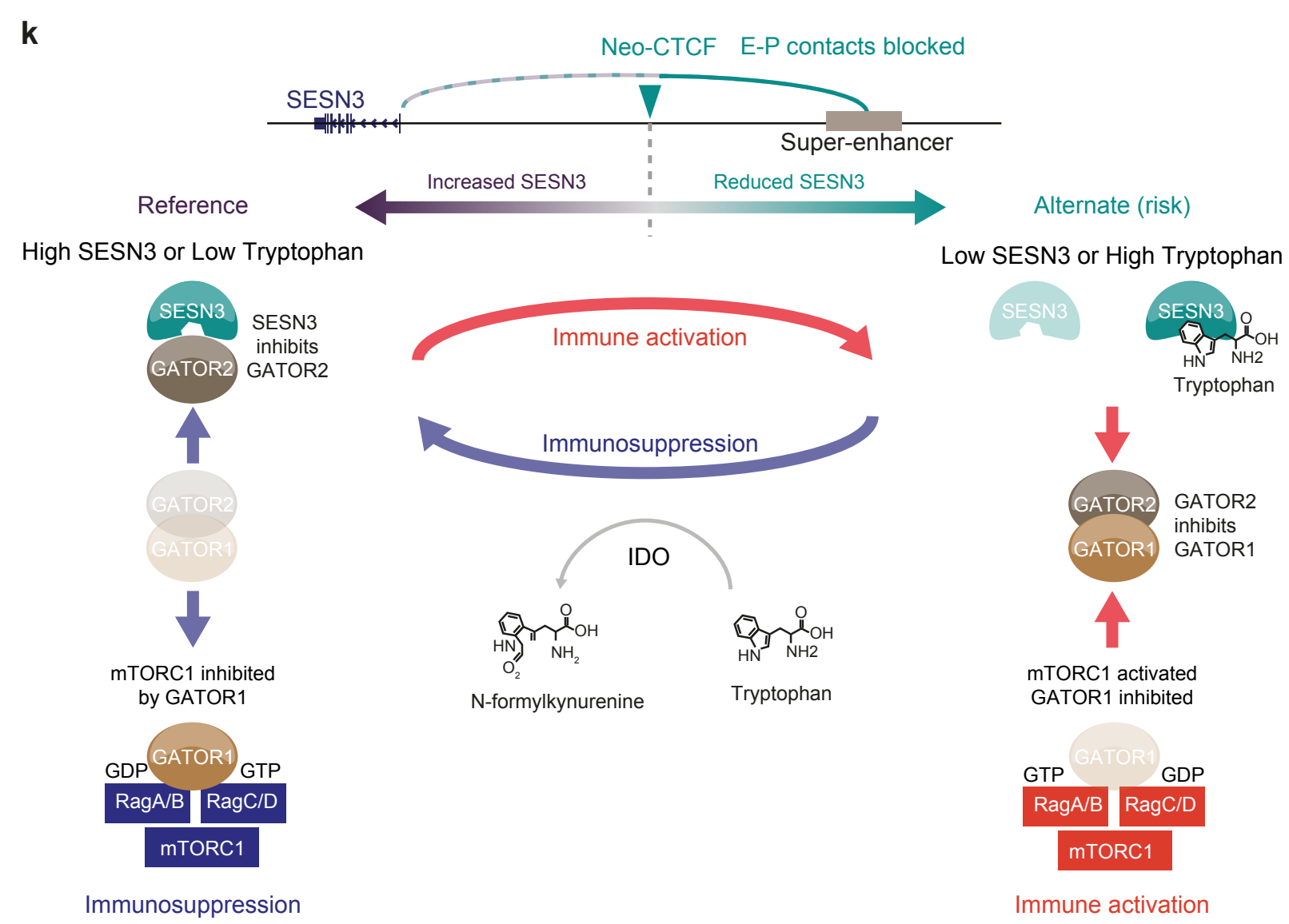

Fig. 6k, Schematic for working model of the effect of rs4409785 on reducing *SESN3* gene expression and in turn how this may affect mTORC1 signalling in starvation conditions, specifically low tryptophan availability.

- Lines 378-380: I actually believe GWAS variant with gain-of-function outcomes have been described before (e.g. PMID: 29056226). Seems better to rephrase.

We thank the reviewer for highlighting this prior work which we now reference in the manuscript. We agree that gain-of-function regulatory GWAS variants have been described previously and have rephrased the text to avoid over-generalisation. In contrast to our study, the variant described in this paper does not alter the CTCF motif itself – it is located 48 bp from the motif (see figure below). In addition, only minor effects on CTCF ChIP-seq are seen. We have reworded the text to make it clear that the novel finding specifically refers to creation of a CTCF motif by a common disease-associated variant.

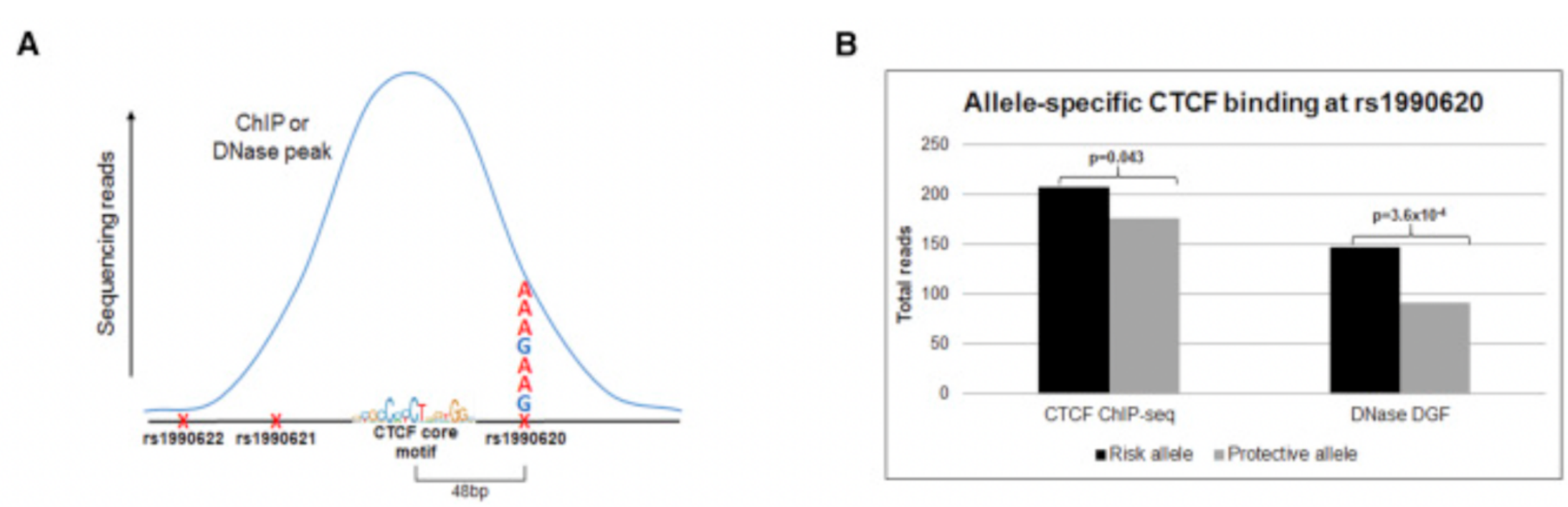

Fig. 5 from Gallagher et al., 2017 (PMID 29056226)

Reviewer #3 (Remarks to the Author):

This manuscript consists of two major parts. First, it describes MCCv, a new take on capture-microC to identify potentially functional sequence variants and their target gene(s) in the human genome. The second part dives deep into a single SNP that was

found to disrupt/create a CTCF site, studying its effects by genome editing and even mouse experiments. Below I will discuss the MCCv section in detail. While the CTCF part in itself is well done and interesting, I feel that it is not a good idea to combine the two parts in one paper. In my view, there is substantially more work to be done to further validate and benchmark the MCCv technology (see below); the manuscript should focus on this and not be overloaded/side-tracked with the biology of a particular variant CTCF site - this will be a nice story on its own. Less can be more!

We thank the reviewer for this suggestion, however, we have discussed this possibility with the editor who feels that the final part of the manuscript should be retained. We also feel that it is important to highlight that individual genetic targets identified by MCCv or other high-throughput approaches require formal validation with multiple orthogonal methods. We also wanted to demonstrate that MCCv can identify new and important biological phenomena.

The MCCv approach has two major aspects. First, like other high-resolution "C" methods, it can identify distal contacts of a putative regulatory element. As was shown before, "C" methods can be allele-specific; here, by applying capture-microC (previously reported by the authors and other labs) with probes that pull down sets of pre-selected SNPs, the authors explore whether they can identify cis-linked genes that may be differentially regulated by the SNP variants. While some earlier papers have explored "C" methods for this purpose, the capture approach presented here seems to provide superior resolution. Second, the manuscript emphasises observed "nano-scale" changes in 3D structure immediately around the SNP. This is highlighted as a novelty in the beginning of the manuscript (and in title/abstract), but plays only a minor role later on (and I did not find it compelling, see below). A third useful feature of the method is that the data can also be used to phase the probed genomic loci, which makes it possible to link the data to allele-specific gene expression.

As the authors mention in their introduction, it is still a challenge to identify functional non-coding SNPs and to identify the target genes. So, in principle, there is a need for new approaches, and MCCv may be a good addition to the noncoding SNP annotation toolbox. However, I have several technical concerns at this point. Most importantly, systematic benchmarking of the method is needed.

We thank the reviewer for this encouraging comment and are very grateful for their suggestion of systematic benchmarking. We believe that this analysis has substantially improved the manuscript and we hope that the reviewer will agree that this shows that MCCv is a good addition to the non-coding SNP annotation toolbox.

Major points:

1. Much of the manuscript leans on the assumption that 3D contacts between distal elements are predictive of functional regulatory interactions. While there are certainly a lot of anecdotes that support this, the critical question here is: how reliable is this assumption? Of 100 of such contacts, how many have an actual effect on gene expression? Is it 2, 10, 50 or 90%? Are most contacts functionally relevant, or mere

bystander effects? This question is relevant here, because the MCCv method is based on the contact-equals-function assumption. The current manuscript does not do a good job to rigorously test this assumption, as it only highlights anecdotal examples.

We thank the reviewer for this thoughtful and important critique. To clarify our views on the assertion that the "*manuscript leans on the assumption that 3D contacts between distal elements are predictive of functional regulatory interactions*". Our belief is not quite in line with this. Rather, our working model is that the absence of detectable contact strongly argues against direct regulation, whereas the presence of contact defines a necessary but not sufficient condition for regulatory potential. This leaves open the possibility that some contacts are permissive or bystander interactions, a point we now make in the discussion: "*This supports a model in which chromatin contact between an enhancer and promoter does not always lead to increases in transcription, however, the absence of contact strongly argues against direct cis-regulation.*"

This is important because it allows the possibility that there are elements that can contact regulatory elements such as the promoters of other genes that are not involved in regulating the gene. We also believe that a nuanced approach has to be undertaken in assessing regulatory function because simple deletion of single regulatory elements does not exclude important regulatory functions. This has been convincingly demonstrated at the alpha globin locus - see Hay et al., *Nature Genetics* 2016, which appears to show that 3 of the 5 enhancers are not functional but the follow up study (Blaney et al., *Cell* 2023) shows that the conserved enhancer elements, which can be deleted individually without altering expression, actually reduce gene expression by 50% if they are all removed. We have also published work showing that gene expression can change with minimal alterations in contacts, for example following depletion of Mediator complex components (see Li et al., *Cell* 2025).

Systematic benchmarking is really needed. This should include an estimate of predictive power and an estimate of false positive rates. Here's what I propose :
Figure 3k appears to show data that can serve a benchmarking purpose to a large extent, but the authors barely mention it. If I understood it correctly, figure 3k shows the RNA-seq allelic (im)balance of all genes with an allele-skewed microC contact with one of the probed SNPs. From these 236 SNP-gene pairs, 21 have significantly imbalanced expression (q-value < 0.05). So, from this basic analysis, it seems that the predictive power for functional interactions is roughly 21/236 = 9%. This number is worth highlighting in the manuscript.

However, this does not fully address the false-positive rate: genes may have significantly imbalanced expression by other causes, for example due to one of the many other SNPs that are in the same LD block. Thus, it needs to be estimated what this "background" rate of imbalanced expression is. For this, one will need a set of control genes that according to MCCv are *not* linked to an imbalanced SNP. For this control set, the imbalance rate also needs to be determined. Let's imagine this rate is 3%; then the false-discovery rate (incorrect assignment of the gene to the SNP) is about 3/9 = 33%. Note that this is different from the FDR on the y-axis of figure 3k, which only estimates the FDR in calling the gene expression imbalanced.

I strongly recommend that the authors include such a quantitative benchmarking calculation. What I propose above is just one possibility (although it seems very feasible, as all data seems to be there); there may be other ways to approach it.

We thank the reviewer for suggesting the need to do systematic benchmarking as well as providing a clear analytical approach for this.

In the original Figure 3k, we included every SNP–gene pair across the donors and we were simply trying to demonstrate allelically imbalanced genes that were linked to disease SNPs. In order to address the reviewer's question, we have improved the analysis to assess the predictive power of the approach. We have now undertaken allele-specific expression of genes which could be reliably phased for both MCCv and RNA. For MCCv, we now include separate analysis for the primary and secondary ranked genes based on strength of contact. In addition, we have improved the RNA-seq analysis such that 25 SNPs within the gene body are required with RNA coverage to be included within the analysis. We ensured that these SNPs were in independent reads by enforcing a minimum distance of the median fragment length in the RNA-seq libraries (153 bp). We have undertaken genome wide analysis of allelic RNA-seq expression to measure a robust background in all 3 donors.

Genome wide analysis showed RNA-seq allelic imbalance at 9.41% of genes across the 3 donors. We also calculated the background suggested by the reviewer, which showed that 7.2% of genes had RNA-seq imbalance based on all SNP–gene pairs where there was no evidence of a significant change in interaction frequency detected by MCCv (enrichment = 0.77, Chi-squared test $p = 0.0991$).

In contrast we found 29.1% RNA-seq allelic imbalance occurred at primary nominated causal genes with changes in MCCv contacts from nominated SNPs. This represents a 3.1-fold enrichment over genome-wide background ($p < 0.0001$) and a 4-fold enrichment over variants with no MCCv change ($p < 0.0001$). Secondary-ranked genes show a weaker enrichment (2.55 ($p = 0.014$) and 3.32 ($p = 0.0021$)), further supporting the ranking strategy. This quantitative benchmarking is now included in the Results and Discussion.

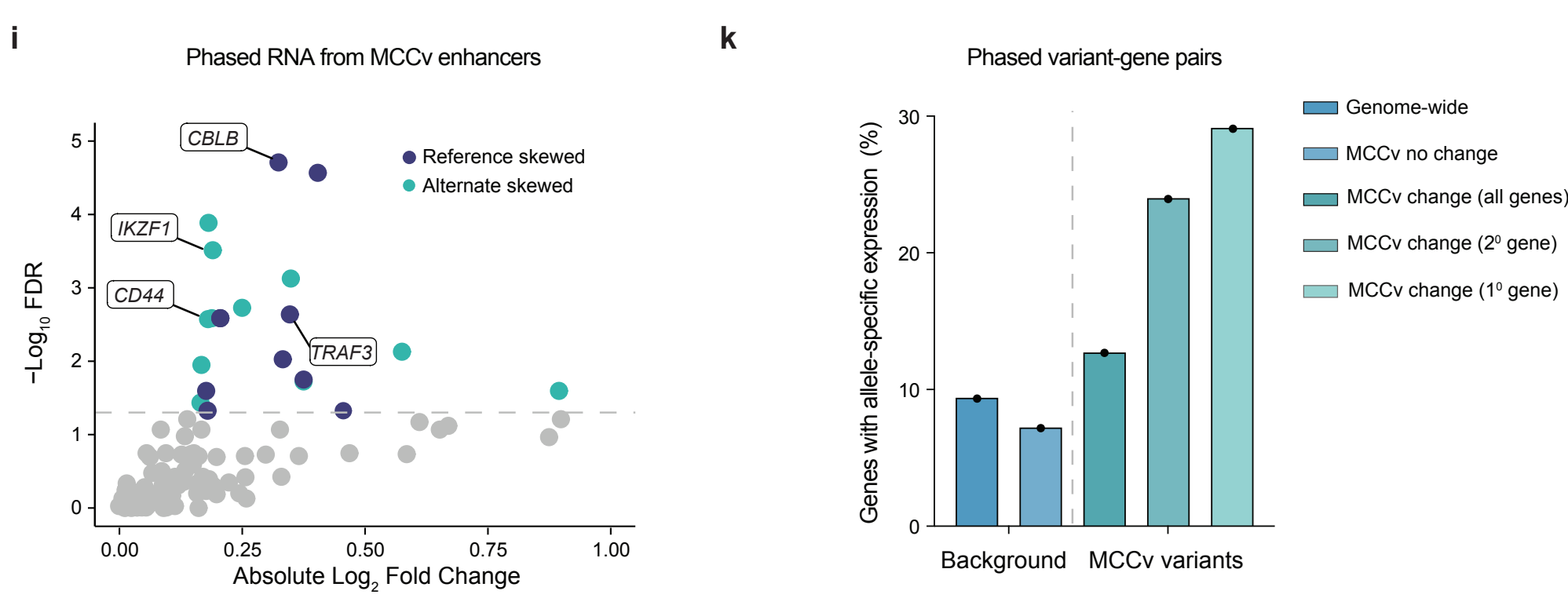

Fig. 3i and k. i, Volcano plot of showing the allelic expression of primary or secondary nominated genes (through MCC based contact frequency ranking system) linked to a heterozygous variant which could be reliably phased (variable n for each gene (n ≥ 25), two-sided Wilcoxon rank-sign test, local FDR estimation using FDRtools with default

parameters). **k,** Percentage of allelically imbalanced genes in a variety of SNP-gene pair categories. Background rate was calculated as the number of allelically imbalanced genes, genome-wide across our three donors. No MCCv change represents all SNPs with no significantly altered MCCv peaks across genotypes and all phased genes linked to those corresponding SNPs (n = 581, enrichment score genome-wide = 0.77, Chi-squared test $p = 0.058$). Variants with a statistically signifcant MCCv change were first tested with all phased genes (n = 660, enrichment score over genome-wide = 1.35 (Chi-squared test $p = 0.002$), enrichment score over MCCv no change = 1.76 (Chi-squared test $p = 0.001$)). These were filtered for only the secondary nominated causal genes (i.e. the gene with second most contacts to corresponding SNP) (n = 25, enrichment score over genome-wide = 2.55 (Chi-squared test $p = 0.014$), enrichment score over MCCv no change = 3.32 (Chi-squared test $p = 0.0021$)). Primary nominated genes for variants with evidence of MCCv changes were tested (n = 48, enrichment score genome-wide = 3.1 (Chi-squared test $p < 0.0001$), enrichment score MCCv no change = 4.03 (Chi-squared test $p < 0.0001$)).

2a. At this stage the nano-scale patterns are not compelling to me. To the untrained eye, Figure 1d looks extremely noisy and it is not clear what kind of objective statistical analysis (if any) the authors applied to decide that the ref and alt alleles really differ. Given the apparently very high noise levels, the cartoon interpretations seem too speculative to me.

We thank the reviewer for raising this. In response to their comment, we have improved the colour representation in the heatmaps to improve clarity (these were originally in CMYK and have been changed to RGB to increase contrast). We have reproduced the data below in greater resolution and included directional windowing of the data. In Figure 1 our intention was to present the raw MCCv nanoscale signal at 1 bp per pixel, rather than a smoothed or processed representation.
In order to circumvent the problem caused by MNase cutting adjacent to DNA bound proteins we have developed a directional windowing system approach based on using a vector from the ligation junction determined by the read directionality of both reads (see Extended Data Fig 3F). We find that this is conceptually difficult to explain and deliberately avoided using this in Figure 1, where many concepts were introduced simultaneously in order to help with figure clarity for the reader.

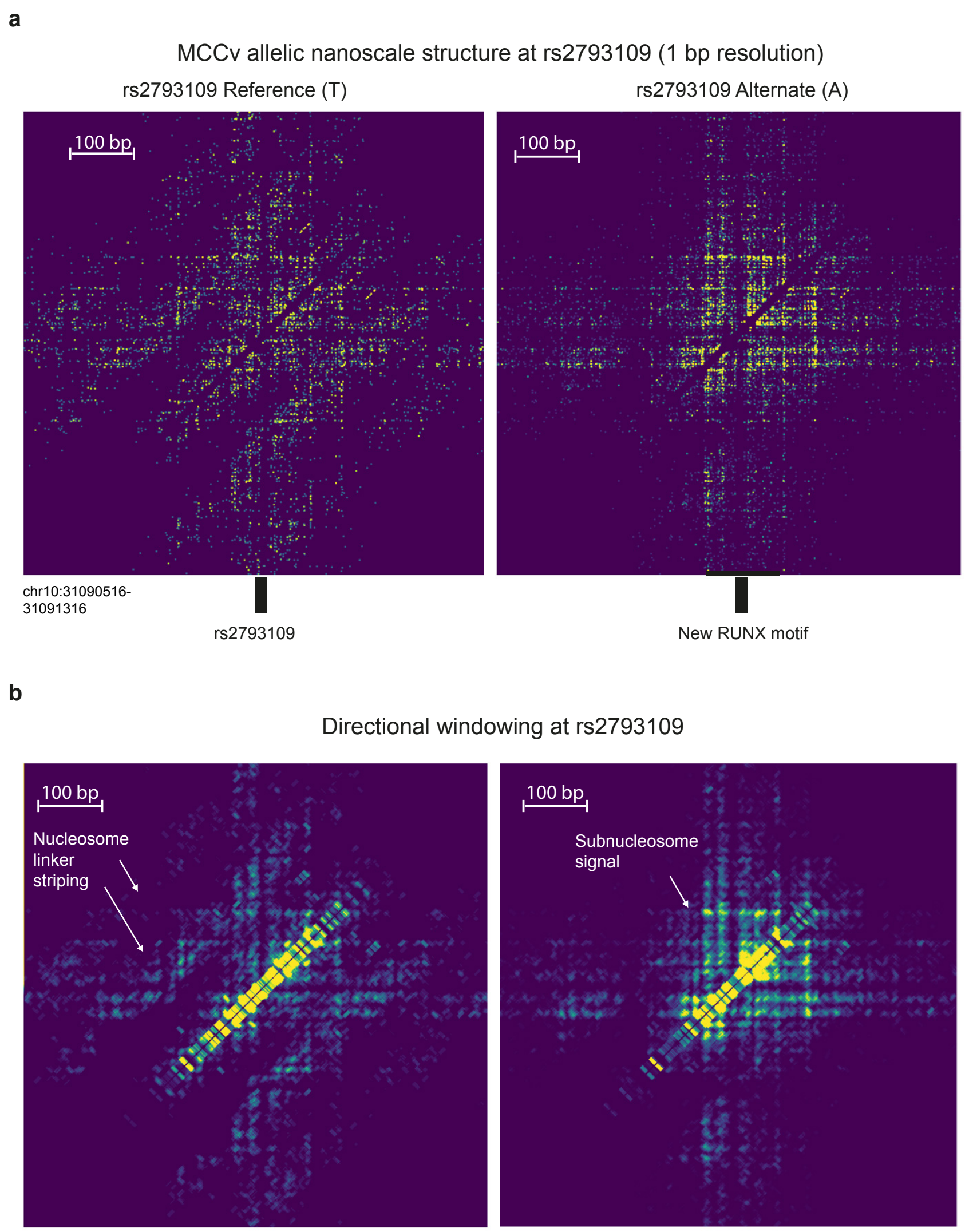

Figure R2. **a,** Nanoscale heatmap visualisation of rs2793109 (1 bp bin size). *Heatmap data is mirrored on the diagonal. Oligo and SNP positions are shown represented by black bar underneath.* ***b,*** *As in* ***a*** *with directional windowing (10 bp windowing).*

To increase our confidence in our interpretations of the nanoscale architectural signal, we generated additional MCC data from an additional 173 sites including 81 in inert chromatin and 92 from active chromatin based on ATAC and ChIP-seq for H3K27ac and H3K4me3. The resulting nanoscale MCCv patterns reproducibly reflect the expected chromatin states and the changes seen allelically at functional variants throughout the manuscript. These new data are now included and described in the revised manuscript as part of Extended Data Figure 1e.

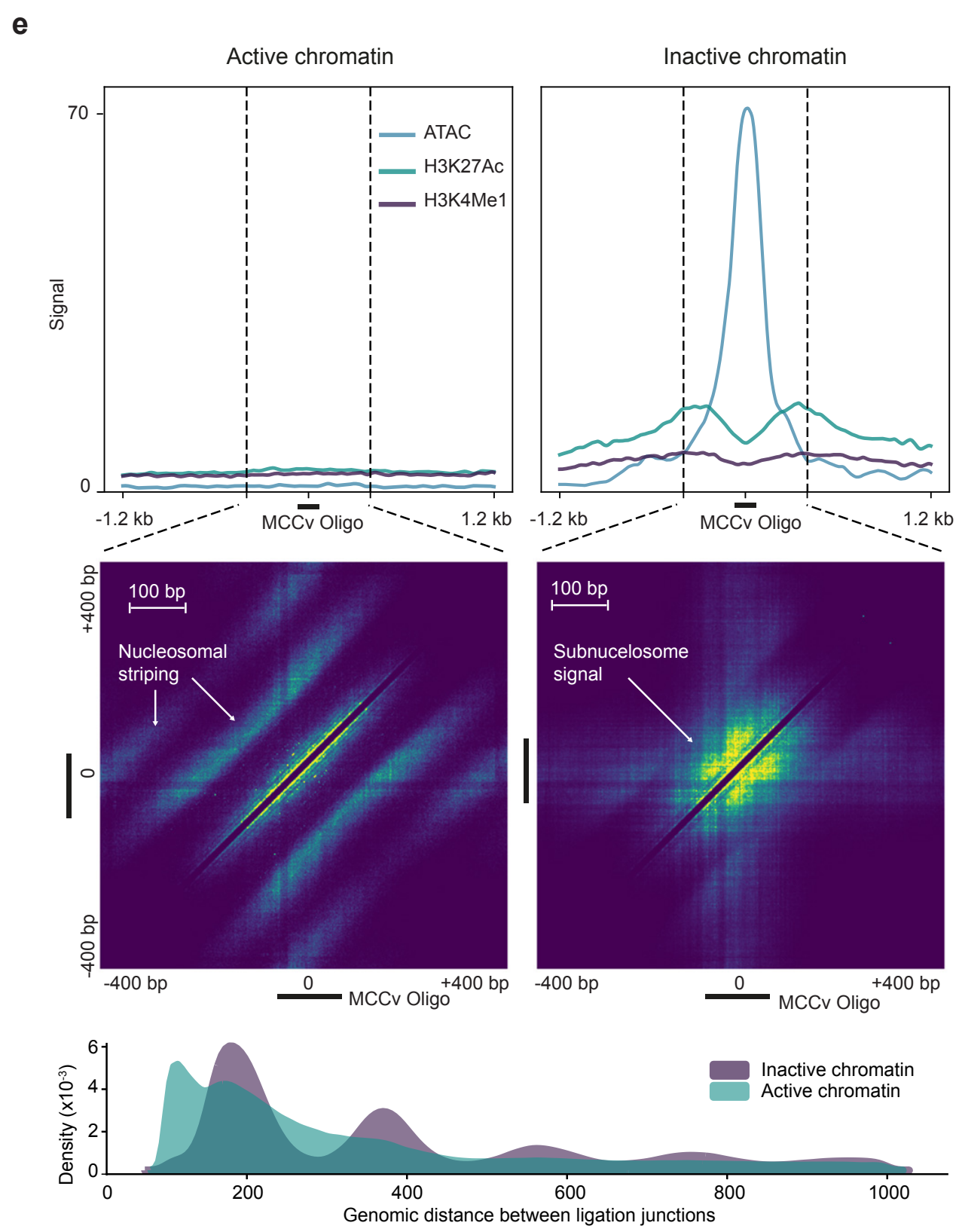

Extended Data Fig. 1e. Control capture of independent sites corresponding to inactive (n = 81) and active chromatin (n = 92). Chromatin signature defined by ATAC-seq and H3K27ac/H3K4me1 ChIP-seq metaplots (2.4 kb region centred around the oligo). Nanoscale metaplots are 1 bp bin size, mirrored on the diagonal, and MCCv oligo position is marked with black bar (800 bp region). Kernal estimate density (KDE) plot shown underneath for the primary genomic distances of short-range unique ligation junction at both inactive chromatin (purple) and active chromatin (green) sites.

We also generated high resolution MCC data from the 2 kb surrounding the variants included in Fig. 3 to provide context for the nanoscale allelic maps and aid with interpretation of these data. We have added these data as part of the **new Extended Data Figure 4 (b-c)**.

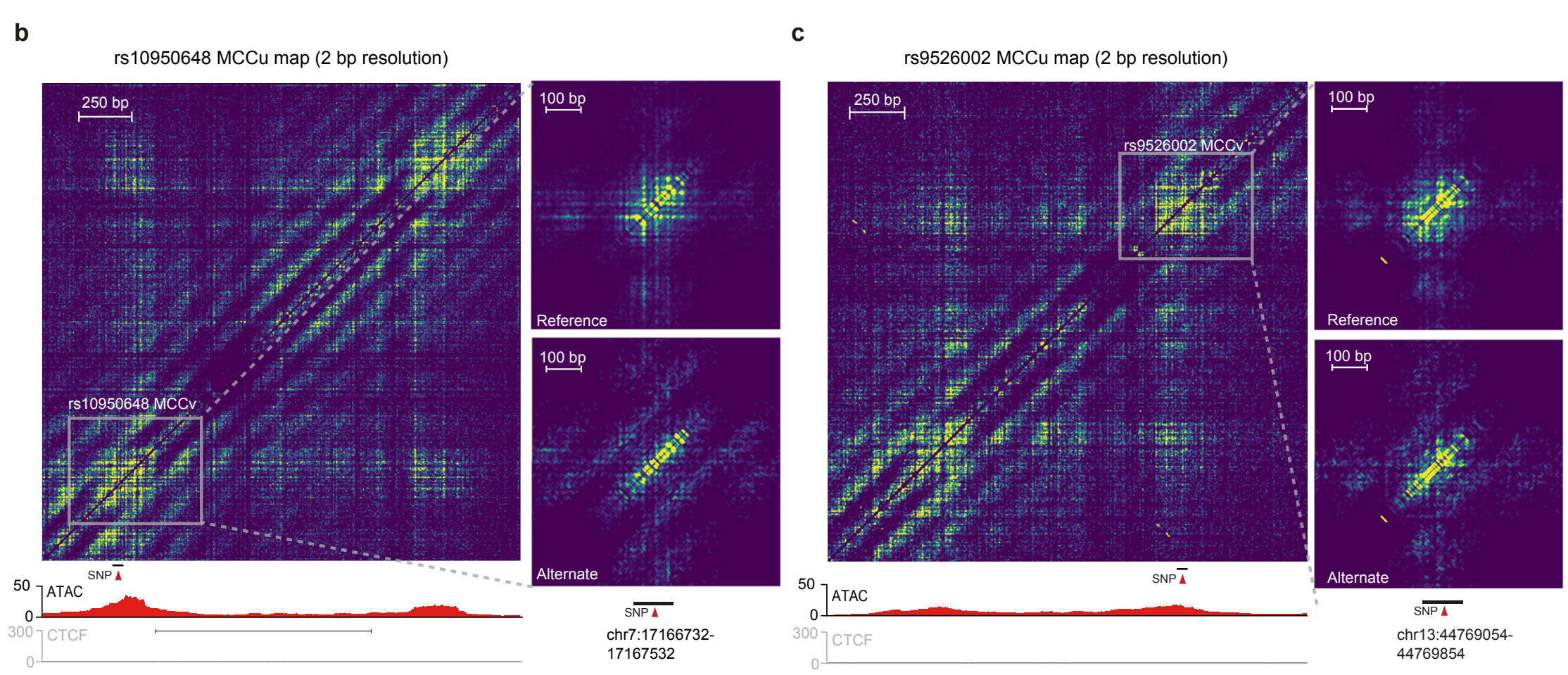

Extended Data Figure 4b and c. MCC ultra tiles over two representative exemplar SNPs (rs10950648 and rs9526002) (n = 6, bin size = 2 bp). Corresponding ATAC ($CD4^+$, unstimulated blue, stimulated red, overlay) and a unified CTCF track are shown underneath. Capture probe/SNP location are shown by horizontal bar and red triangle respectively. Zoom in shows MCCv single allele directional windowed heatmaps for reference and alternate alleles at the respective SNP shown in **Fig. 3f,g**.

There is also no evaluation of the reproducibility of this pattern: how do *independent biological* replicates compare? What did the other 44 tested SNPs look like? An extended "gallery" figure is needed to show all 45, including genomic coordinates (and the processed data should be made available). This will enable the reader to get a clear view on the kind of nano-patterns that can be obtained. A statistical test to evaluate allelic differences will be needed.

We thank the reviewer for this important point regarding reproducibility, transparency, and statistical evaluation of the nanoscale patterns. For full transparency all the nanoscale heatmaps from all experiments have been uploaded to Zenodo (https://doi.org/10.5281/zenodo.18404259) with the genomic coordinates. This includes data from over 500 alleles. We have not been able to create a gallery figure as requested because it is not possible to create a pdf with the required resolution for this many heatmaps, but we hope the full access to the processed nanoscale maps is sufficient.

The maps are made by merging 6 independent MCC replicates (from a single donor or cell line) in order to reduce the sparsity in the signal. Below is an example variant where we show the matrices for both alleles in 12 replicates across two donors, demonstrating increased sparsity but also a shared pattern between the independent replicates. This demonstrates that for the example variant, the nanoscale patterns are more distinct between genotypes than within replicates of the same allele.

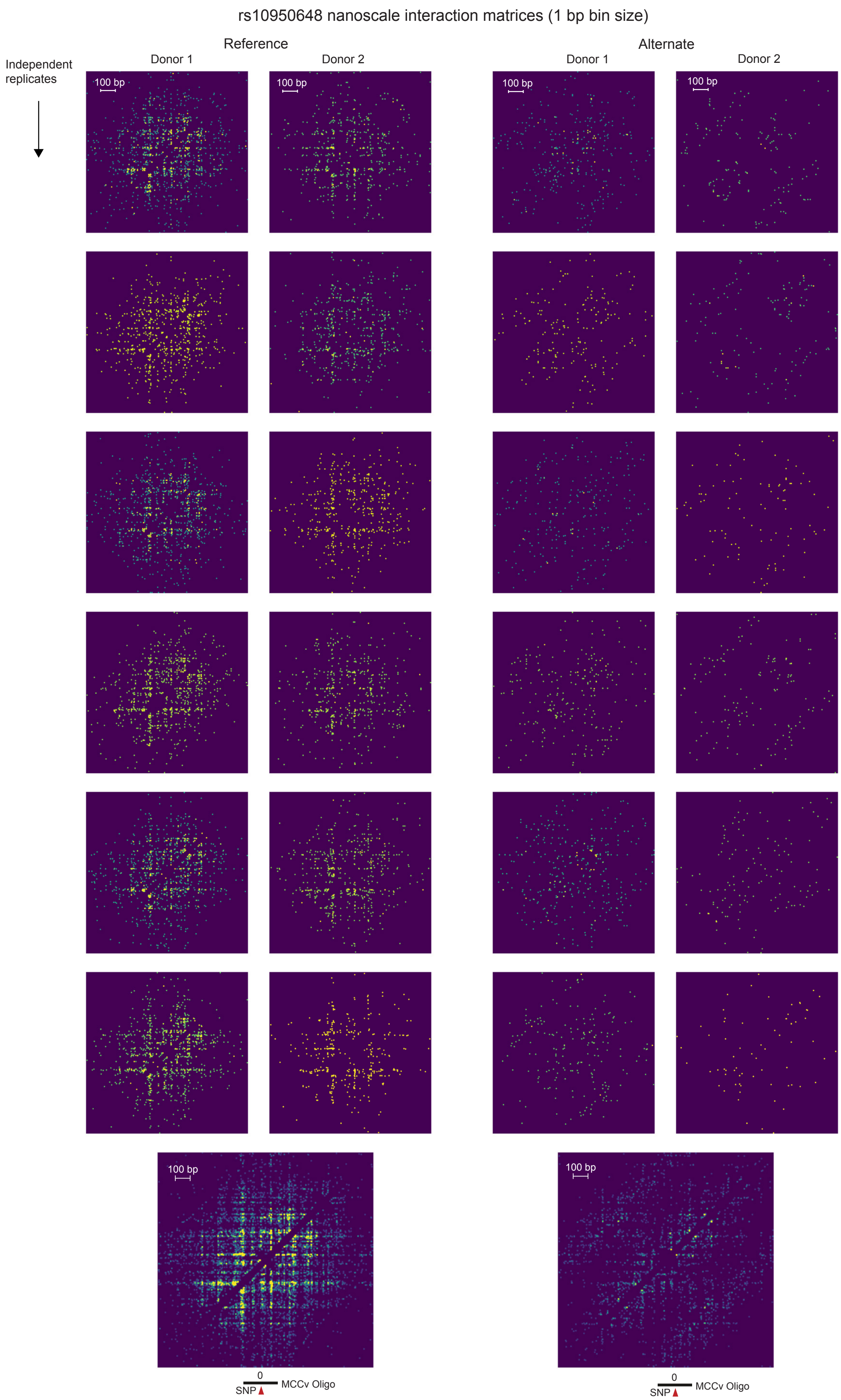
rs10950648 nanoscale interaction matrices (1 bp bin size)
Reference
Alternate
Donor 1
Donor 2
Donor 1
Donor 2
Independent replicates
100 bp
0
SNP
MCCv Oligo
0
SNP
MCCv Oligo

Figure R3. Nanoscale interaction matrices (400 bp window) from rs10950648 variant alleles (reference and alternate), including independent MCC replicates from the same donor (n = 6) across two donors (total n = 12). The final merged matrices, which are shown throughout the study, are included for comparison.

We agree that robust statistical testing of the maps across genotype is required in order to increase the confidence in our conclusions as well as for benchmarking. During the first submission we were in the process of developing a dedicated package to statistically compare base pair resolution matrices (basepairC), which we have now completed and applied. This is now described in the methods ("Statistical analysis of MCCv nanoscale matrices").

Briefly, this package:

I. Normalises the matrices using Trimmed Mean of M-values (TMM) to account for coverage differences between sample matrices,
II. An independent generalised linear model for each allele was used to test for allele-specific differences using a quasipoisson distribution to account for count overdispersion,
III. Corrects for multiple testing using Bonferroni correction.

We applied basepairC to both our Jurkat control variants and T cell disease associated variants. 47.6% of our variants tested (n = 286) had both statistically and practically significant differences between reference and alternate alleles when applying a stringent effective significance cut off at an absolute β-coefficient (effect size) ≥ 0.2 and $P_{adj} \leq 0.01$.

As an internal reproducibility control, we ran basepairC iteratively on random combinations of replicates combined from reference and alternate alleles. This confirms that the majority of nanoscale changes are not driven by replicate-to-replicate variability, but more likely due to genotype differences.

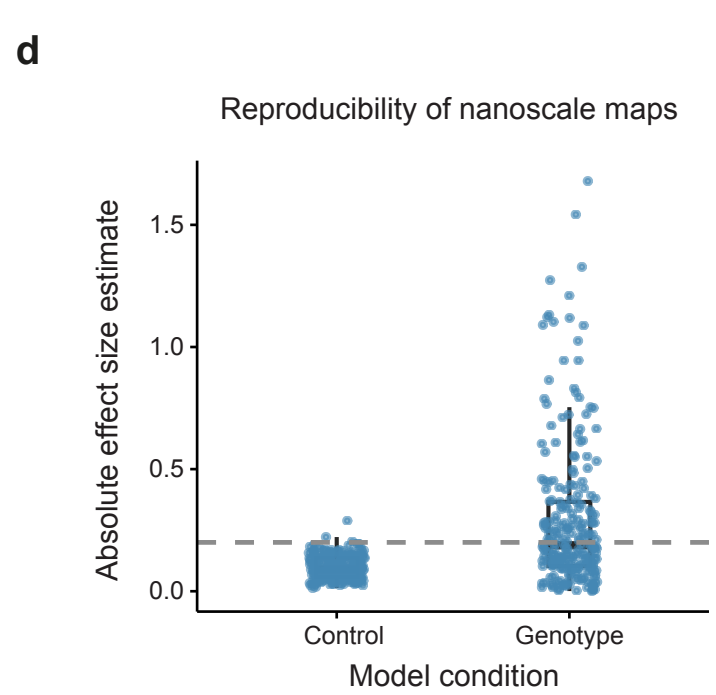

Extended Data Fig. 5d. Effect size estimate of GLM from basepairC package at tested SNPs (n = 231) (bottom 20% of variants based on matrix read counts were filtered). Estimates were done between maps of different genotypes (i.e. allele-specific) (Genotype, mean β-coefficient (effect size) = 0.293). As a control random combinations of 6 reference and 6 alternate allele sample matrices were compared.

Taken together, the availability of all nanoscale data, direct demonstration of biological reproducibility across donors, and the introduction of a formal statistical framework provide a robust and transparent evaluation of allele-specific nanoscale chromatin patterns.

Extended Figure 1h (MYB/TAL1) also looks very noisy, and reproducibility is not documented - even the nucleosome stripes seem virtually absent: how do the authors explain this?

We thank the reviewer for highlighting this point. We have now included the statistical analysis of the difference in these maps. We have also included a density plot of ligation junction distances, which demonstrates the nucleosomal pattern in the inactive reference allele compared to the MYB-indel allele.
This indel has a more subtle effect than some of the other examples in the manuscript probably because the enhancer is partially active even in the absence of the indel. Interestingly, in the reference allele the region contains GATA and RUNX motifs, which line up with the potentially active region on the reference allele (towards the right off-centre – shown in the figure below, and added to Extended Data Fig. 1h). In the Mansour et al., paper (Science 2014) they show that GATA3 and RUNX1 bind this site. This is in keeping with the ATAC-seq data, which skews only 68% toward the indel and provides evidence of partial accessibility in the reference allele.

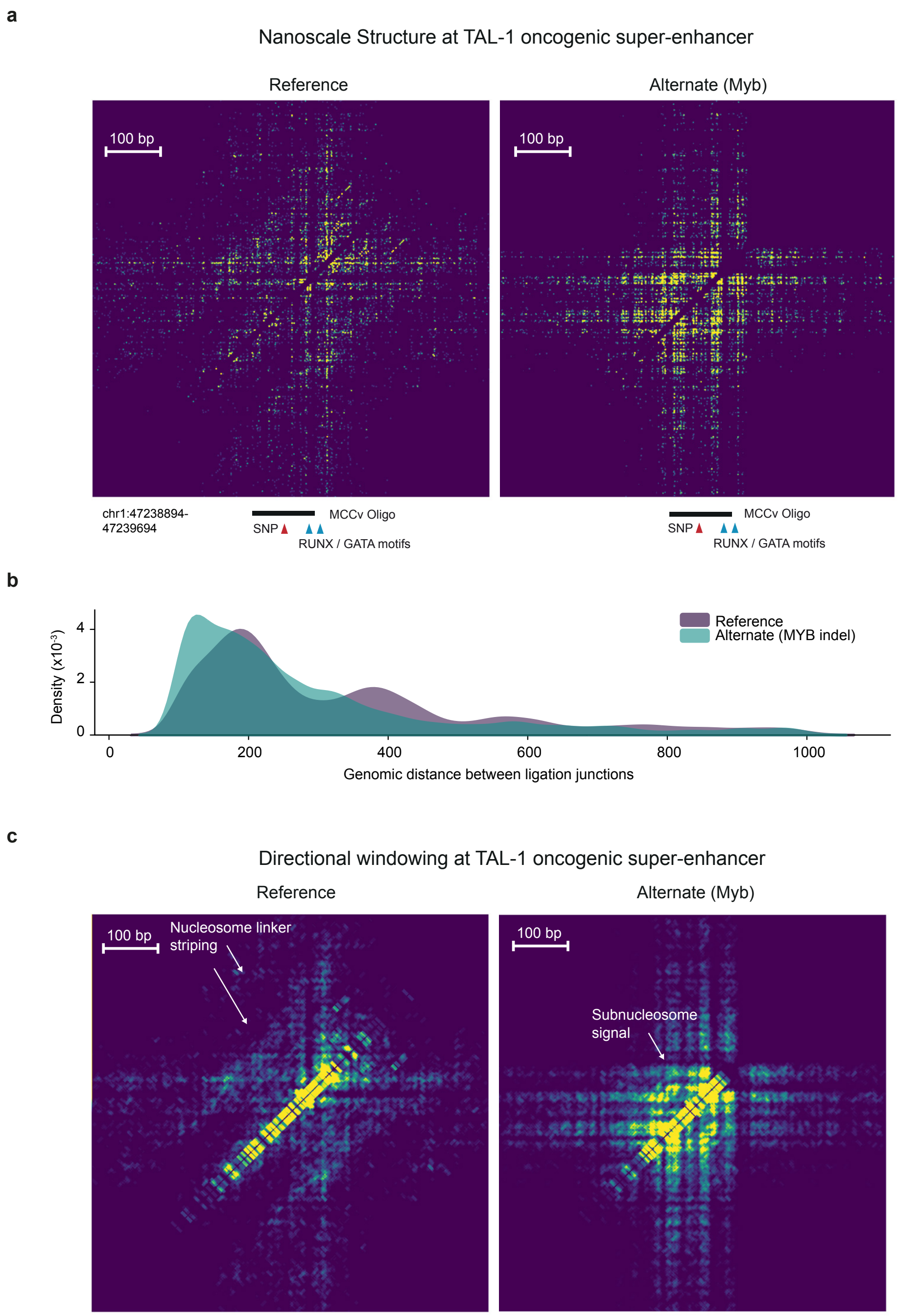

Figure R4. ***a,*** *Nanoscale heatmap visualisation of the oncogenic MYB indel (1 bp bin size). Heatmap data is mirrored on the diagonal. Oligo and SNP positions are shown represented by the black bar and red triangle respectively (basepairC $\beta = 1.02$, $P < 0.0001$) .* ***b,*** *Kernel density estimate (KDE) plot of genomic distance between two base-pairs which make up short-range ligation junctions for both reference and alternate alleles of the MYB indel.* ***c,*** *As in* ***a*** *with directional windowing (10 bp bin size).* ***d,*** *As in* ***c*** *with axis centred on horizontal analogous to more standard Hi-C like visualisations of 3C data.*

2b. Conceptually, the starting point of figure 1 seems set up as a self-fulfilling prophecy. The 45 SNPs were chosen based on very strong allelic differences in ATAC-seq signals (Extended Figure 1c). Given that the active allele typically has a nucleosome-free region, it is not so surprising that MCCv sees a difference in the fine cross-linking pattern. The

real test is: if no prior selection was done based on ATAC-seq, would nano-scale patterns be discernable and useful to identify functional regulatory elements? Again, this would require an objective statistical scoring system to gauge the nano-patterns. It would also require rigorous benchmarking against ATAC-seq as method to detect allelic skews at nano-scale, because ATAC-seq is a much simpler assay than MCCv.

The rationale for choosing variants which display evidence of a gain-of-function on the alternate allele in ATAC-seq, was to ensure all initial variants chosen were likely to be functional in regard to enhancer activity and circumvent the possibility that preferential mapping to the reference allele might contribute to changes. These acted as positive controls, so that we could test to see if MCCv was sensitive enough to pick up 3C changes at sites where we had evidence of variant causality. Although we agree there is an element of circularity in the argument, it served as a benchmarking experiment for rest of the study, and we have clarified the motivation in the text.

Figure 3 details our exploration of disease-associated variants where no prior selection was done based on ATAC skew. Here a lower percentage of SNPs displayed MCCv changes (vs. the positive Jurkat controls) both in terms of long-range contacts (54% vs 61.9%) and nanoscale patterns (41.0% vs 85.7%). This is to be expected, as some variants may be in a disease associated haplotype and intersect ATAC but be non-functional.

j

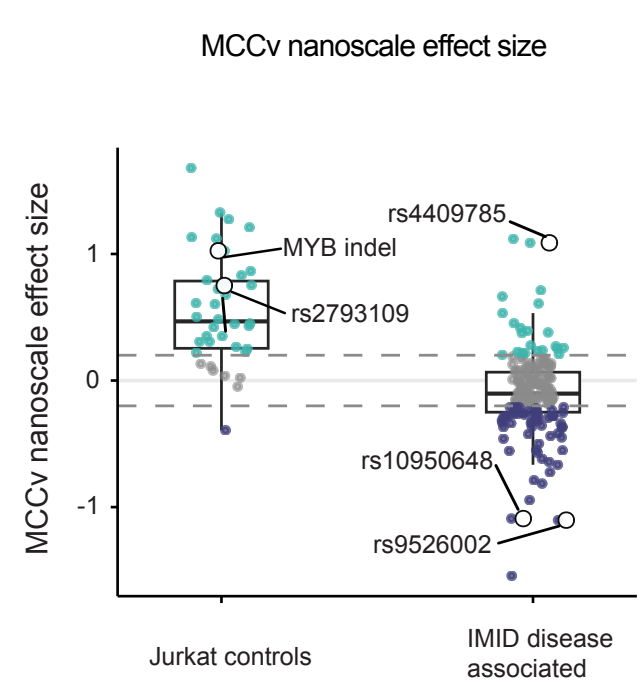

Extended Data Fig. 3j. Effect size from basepairC matrix difference modelling in Jurkat controls (prioritised using ATAC skew) vs. T cell disease associated enhancers (prioritised by open chromatin intersection only). Variants within the lowest 10% of read counts for MCCv nanoscale maps were filtered from the correlation to remove outliers based on read counts. Gain-of-function (MCCv nanoscale effect size ≥ 0.2) and loss-of-function (MCCv nanoscale effect size ≤ -0.2) variants are highlighted in green and purple respectively. Variants highlighted throughout the study are indicated on the plot.

In terms of benchmarking against ATAC-seq we have now shown correlation between the effect size estimate of our nanoscale maps from basepairC and ATAC-seq skew. This demonstrates that both methods are able to assign regulatory function to variants. We agree with the reviewer that ATAC-seq is a much simpler assay and therefore will still remain a powerful way to identify functional variants. However, we see MCCv as being highly complementary, as not only does it also assign functionality, but it can identify the interacting gene simultaneously, which is not possible from ATAC-seq data alone.

k

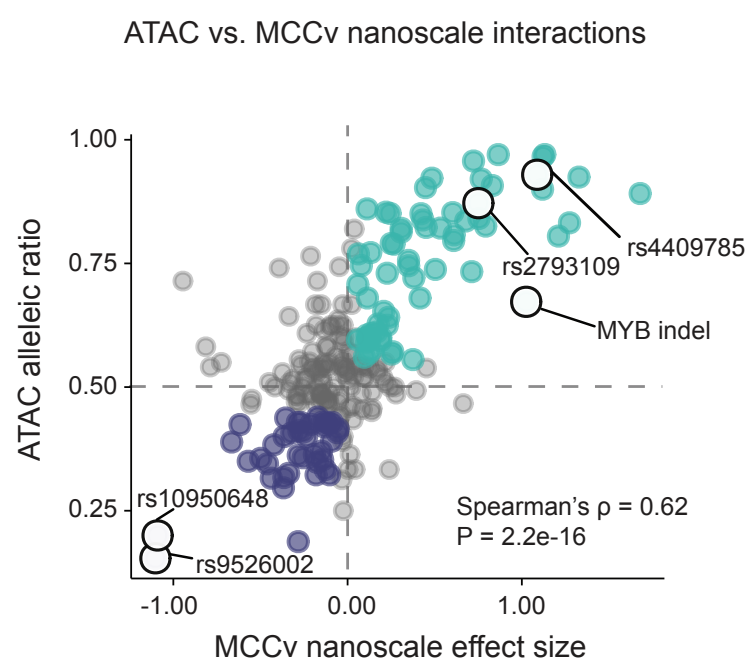

Extended Data Fig. 1k. Allelic ratio from cell ATAC sequencing versus effect size from basepairC matrix difference modelling (n = 201, Spearman's ρ = 0.63, $p = 2.2 \times 10^{-16}$). Variants within the lowest 10% of read counts for either ATAC or MCCv nanoscale maps were filtered from the correlation to remove outliers based on read counts. Gain-of-function (ATAC allelic ratio ≥ 0.55 & MCCv nanoscale effect size ≥ 0.05) and loss-of-function (ATAC allelic ratio ≤ 0.45 & MCCv nanoscale effect size ≤ -0.05) variants are highlighted in green and purple respectively.

The other important advantage of MCCv is the ability to generate fine scale structures within the nucleosome depleted region at the *cis*-regulatory element, which potentially allow transcription factor binding sites to be identified. In response to the reviewer's comment we have now compared ATAC-seq footprinting with MCCv. With conventional ATAC-seq, after filtering of reads for the variant, only ~10 reads map on average to each allele. To assist comparison, we used oligonucleotide capture of the ATAC-seq library from one of the heterozygous donors for rs10950648 and rs9526002 to generate data with around 1000 PCR-deduplicated ATAC reads (which exhausts the ATAC-seq library complexity). The ATAC-seq data were divided into reference and alternate alleles. Even with target enrichment the ATAC-seq has limited utility for identifying allele specific transcription factor binding sites. Interestingly, visualisation of the 3C component of the MCCv data greatly strengthens the ability to visualise changes between alleles compared to conventional footprinting (which only uses the collapsed ligation junction positions).

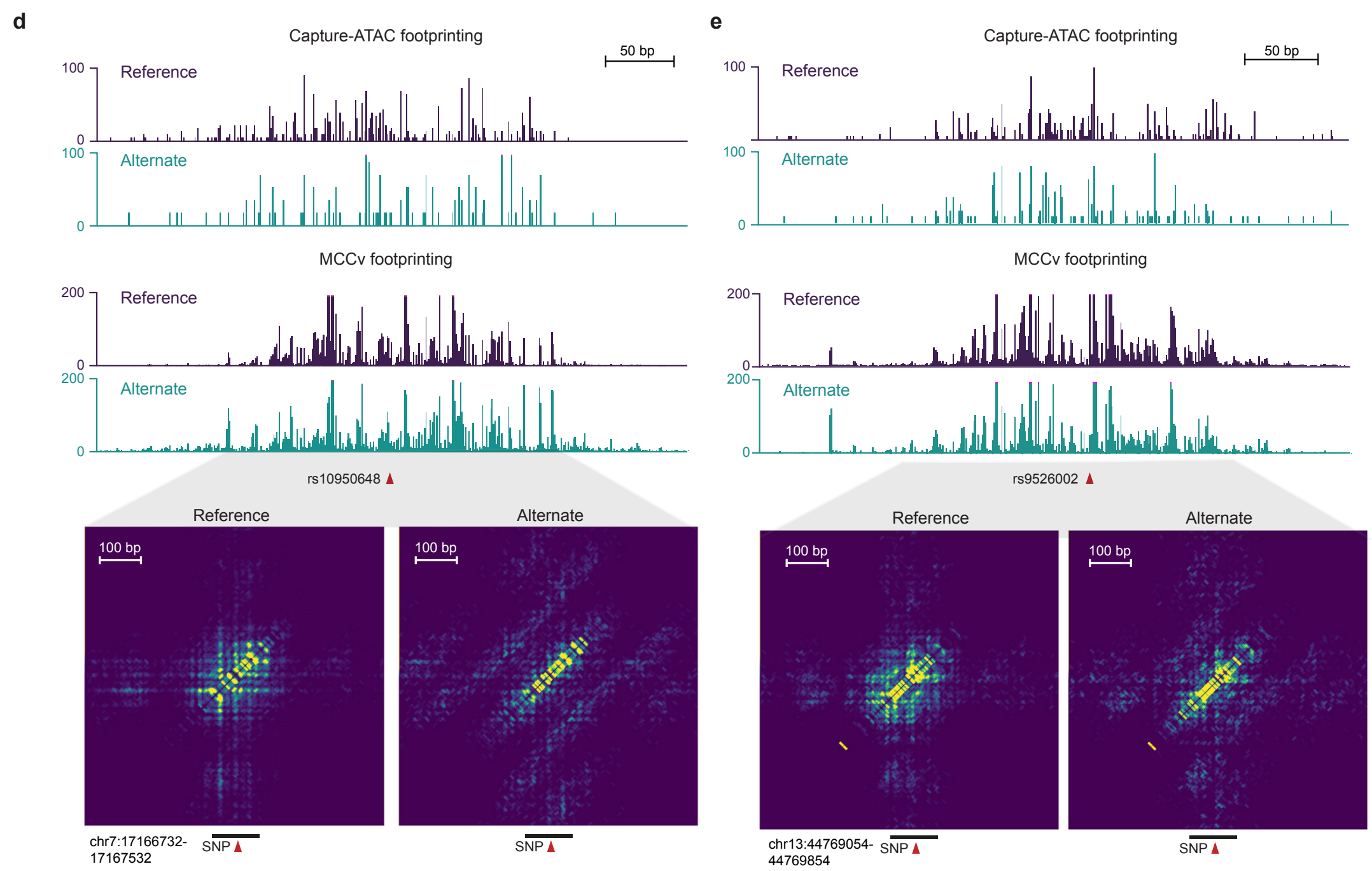

Extended Data Fig. 4d,e. d, Footprinting data from ATAC-seq combined with MCCv based capture enrichment (above) and MCCv footprinting for both reference and alternate alleles of rs10950648. SNP is indicated with red triangle. Below is the direction windowed heatmap for these alleles using the 3C component of the ultra-short ligation junctions, which reveals clearer allele-specific differences. **e,** As **d** for rs9526002.

3. Figure 1g: FDR scores of peaks are calculated (by which method? please specify), but it is not clear whether the underlying statistical model takes reproducibility between independent biological replicates into account.

We apologise that this wasn't clear from the manuscript, the FDRtools - (r package - https://doi.org/10.1093/bioinformatics/btn209) was used to estimate local FDR with default parameters. This estimates empirical null distributions from the observed test statistics to control the false discovery rate. Peaks were first identified on merged MCC replicates to define quantification windows between alleles, but statistical testing explicitly incorporated reproducibility by quantifying junction counts from at least six independent replicates per allele and comparing alleles using a paired *t*-test; the resulting *p*-values were then corrected for multiple testing using FDRtools as described to obtain corrected *p*-values.

4. Figure 1i: It is not clear how many biological replicates this analysis is based on. If the error bars indicate the distribution of values of the 15 or 16 variants, then a t-test is not appropriate, because these variants are linked and hence not independent measurements (independence is a requirement for statistical testing). Instead, multiple biological replicates are needed, variant data from a single gene should be somehow combined/averaged per replicate, and then a t-test (or other test) across biological replicates should assess statistical significance. Same applies to figures 3i and 5h.
The error bars reflect the distribution of RNA-seq reads from phased variants within the gene across replicates. These variants are separated by a mean of 6.8 kb (median 1.8 kb)

within the gene and we removed variants that were separated by < 150 base-pairs to prevent reads containing more than one variant from being included as independent datapoints. Each measurement of the RNA-seq depth at a given variant is therefore likely to be derived from independent RNA fragments in different parts of the gene body. This approach is analogous to quantifying expression at multiple independent positions across a gene body (e.g. qPCR amplicons at distinct loci). We have also changed the analysis so that we now use a Wilcoxon rank sign test (as was used for analysis of other RNA data in Figure 3i and 5h). We have clarified the details of this analysis in the methods section.

5. In figure 3k about half of the significant genes have a ref/alt skew, the other half a alt/ref skew. Does this match the skews in the nano-patterns (e.g. as shown in 3f,g)?

Among SNP-gene pairs showing statistically significant allelic imbalance (FDR < 0.05) where the SNP has either evidence of long-range changes in the MCCv profile or MCCv nanoscale maps there is concordance in the directionality with RNA-seq at 90% of the primary nominated genes. When using the nanoscale patterns in isolation the directionality matches at 70% of loci.

6. Please comment in the Discussion on the throughput limits of MCCv. What is the maximum number of SNPs that can be tested in parallel? In some experiments as many as 6 technical x 3 biological replicates were done. What does this mean in terms of labor, costs and scalability?

We agree with the reviewer that such a discussion point is important for the wider community. In summary, we cite previous work from our laboratory (https://doi.org/10.1038/s41467-020-20809-6), which demonstrates the upper limit of our capture system is around 1000 probes/SNPs. However, we perform parallel PCRs during library prep to ensure we have sufficient material to allow 10-100 different captures (each with 1000 oligonucleotides) to be performed on each library, allowing in theory 10,000-100,000 SNPs to be analysed. In terms of the captures themselves, 6 independent technical replicates (separate MNase digestion/ligations) can be pooled and captured in 1 tube. Therefore 3 captures (e.g. 3 donors) can be done in 3 tubes and in parallel, thus minimally impacting labour. Each SNP requires ~100,000 reads per library (technical replicate), so increasing the number of SNPs will increase the cost of sequencing but this is a minor consideration compared to the overall costs of the reagents.

Minor points:

7. Legend Fig 1c: "Note that the active (alternate) alleles do not follow a nucleosomal pattern" - to my eye there is a clear nucleosomal pattern, it is just weaker. In the "nano-structure" heatmaps it may also be useful to add arrows or other marks to the structures that the reader should pay attention to; it took me several minutes to spot the (putative) differences.

We thank the reviewer for pointing this out - we have changed this to read "...displays weaker nucleosomal patterning." In addition, we have added additional annotation of this figure, which we hope improves clarity.

8. Line 136: text: it is not clear whether the MYB/TAL1 observation was part of the same set of 45 SNPs, or a newly designed experiment altogether.

The MYB/TAL1 observation is from the same experiment and we have clarified this in the manuscript.

9. Extended Figure 2f does not speak for itself, and the legend is too brief. From this info I could not gather how the algorithm works.

Thank you for pointing this out. We have now improved the figure legend for Figure 2f.

10. Lines 211-213: "This approach revealed interactions between transcription factor binding sites on the reference allele": over-interpretation, at this point there is no evidence that these are TF binding sites.

We have adjusted the wording here to avoid over-interpretation.

11. Figure 3k [related to major point above] : the text and figure legend are a bit confusing: main text says "We extended this analysis to all genes with an MCCv contact", legend says "allelic expression of genes prioritised by MCC contact data". So is it MCC or MCCv? Please also spell out what "prioritised" means.

Thank you for picking this up – this should read "MCC" not "MCCv" since we looked at all SNP-gene pairs with an MCC contact, rather than only contacts which demonstrate allele-specific changes. We have also improved the clarity of the figure legend.

12. Throughout the manuscript some elements are referred to "enhancer" even though their enhancer function is not shown (yet). Better to refer to them as "putative enhancer" or similar wording.

We have adjusted the manuscript in line with this suggestion.

13. The comparisons to conventional microC seem not entirely fair, as it depends largely on the relative sequencing depth. What if the microC samples were sequenced much deeper? If this is cost-prohibitive, then it would be good to explain what number of reads would be required to match the depth of MCCv.

Thank you for this suggestion. In the major MCCv experiment reported in this manuscript we define on average 182,663 ligation junctions from a single view-point (max = 696,369), which is 120 bp wide. This equates to over 1500 junctions from each base pair.
We think that the Micro-C papers are excellent and there is no doubt that the data are impressive in scale (Krietenstein et al., 2020 reports 2.5-4.5 billion reads per sample and Hsieh et al., 2020 report 3.3 billion reads from combining 38 samples). In our Mirco-C

data we report 698,913,431 valid pairs, which equates to around 0.227 junctions per base-pair.
However, from any individual site in the genome the sensitivity and resolution of Micro-C is poor compared to MCC. This is because 3 billion read pairs equates to approximately one read pair per base pair in the human or mouse genome (Krietenstein et al., are explicit in their paper that they report ~150 read pairs from each nucleosome). One would have to sequence the Micro-C libraries with approximately 4 trillion reads to obtain data of similar depth (the equivalent of sequencing around 10,000 whole human genomes for each sample).
In addition, to sequencing there are technical improvements in MCC that enable higher resolution data to be generated compared to Micro-C, so even sequencing Micro-C libraries with extreme depth or the use of oligo capture (as in RCMC) does not generate data with resolution below 200 bp. This includes the use digitonin to prevent disruption of nuclear architecture and differences in the 3C library preparation, which allow the whole read fragment to be sequenced and reconstructed to enable ligation junctions to be identified at base pair resolution.

14. It may be appropriate to cite and discuss PMID 39289357.

Thank you for this suggestion, we have now included this in the discussion section of the paper.

We would like to thank the reviewers for their helpful and thoughtful comments and hope these new experiments and revisions address their comments. We believe that the revised manuscript has been greatly strengthened by the review process and we are optimistic that this work will serve as a timely and impactful contribution to the field and to *Nature Genetics*.

Thank you for reviewing our revised manuscript, ‘*Single-allele nanoscale mapping of regulatory variant function*’. We very much appreciate the time you have taken to review our revised study and your constructive comments, which we believe have resulted in further improvements to the manuscript.

Point by point response to reviewer’s comments (in blue)

Reviewer 1 (Remarks to the Author):

The authors have made numerous improvements and addressed all of my comments.

We thank the reviewer for their positive comments regarding our manuscript throughout the review process.

Additional suggestions:

• For the new Fig 2h, could the authors add “closest gene” (and perhaps “2nd closest gene”) as a baseline for comparison?

We thank the reviewer for this suggestion and have now added the enrichment scores for the “closest” and “2nd closest” genes within Figure 2h.

• Re: Point 3, thanks for the explanation. Please add this explanation to Methods?

We have added this point to the Methods under section “MCCv peak quantification comparison”.

Reviewer #1 (Remarks on code availability):

The code repositories are now available and have some documentation. I did not attempt to run code.

Reviewer #2 (Remarks to the Author):

The authors have appropriately addressed all my comments.

We thank the reviewer for their positive comments regarding our manuscript throughout the review process.

Reviewer #2 (Remarks on code availability):

I did not review the code as it goes beyond my own expertise. Of note: this research group has an outstanding reputation when it comes to developing new analysis software for C-technologies.

We thank the reviewer for their comments regarding our previous work developing software for 3C based techniques.

Reviewer #3 (Remarks to the Author):

The revised manuscript has improved substantially, see specific points below. Whether the SESN3 part should be included is indeed up to the Editor, but the description of the MCCv method and data is now very condensed and not always easy to understand. More room for this key part would benefit the overall clarity.

We thank the reviewer for recognising the improvement in the revised manuscript. We have expanded the description of the MCCv methodology and data within the results section.

One major remark: Title and Abstract very strongly state that this is a variant-to-function assay, and that target genes from 405 cis-regulatory elements are identified. This is rather misleading; strictly speaking, it is a variant-to-contact assay, and it seems that functional contacts are detected at rather low frequencies and with rather high FDR. I recommend that title and abstract clarify this by providing specific numbers; please see point 1 below.

We thank the reviewer for this comment and have revised the title, abstract and manuscript to add specificity to claims of functionality. We have also expanded the discussion, which we hope presents the capabilities and limitations of the platform more clearly.
We understand the reviewer's concern that MCCv primarily measures chromatin contacts. However, in our view, "functional" variants include those that alter regulatory element structure or the contact frequency between the variant and other *cis*-regulatory elements in addition to those that detectably alter RNA expression. In contrast, non-functional variants should be completely inert. In this study, we show that the platform can provide evidence of a variant influencing all three of these outcomes, including nanoscale chromatin architecture (effect on enhancer), long-range chromatin contacts and, in a subset of cases, allelic imbalance of target genes.
We note that restricting the assignment of function solely to variants with detectable changes in allelic RNA imbalance may miss biologically relevant variants, when there is clear evidence that they alter regulatory architecture. Many SNPs identified by GWAS confer small changes in disease risk that occur over many years and are expected to have modest, context-dependent effects that may not be detectable with RNA-seq.
In contrast to other methods (such as Mendelian randomisation or colocalization-based eQTL analysis), our platform is able to identify variants within the haplotype that are likely to cause the regulatory effect. This is important because it allows downstream validation of the SNP (for example, using MCC combined with genome editing) and we believe it is important to go on to validate the gene itself (hence the extensive characterisation of *SESN3* in this paper). This was the primary motivation for describing MCCv as a variant-

to-function platform. We have now included in the discussion that variants and their linked genes require orthogonal validation.

Regarding previous major comments (numbering as in previous report):

1. I am pleased that a quantitative analysis was included figure 3k. But the main text glosses over this critical aspect: it is mentioned in only one sentence (lines 266-269) that does not clarify which proportion of tested elements can now be assigned to a target gene, at which estimated FDR. The figure legend is also rather cryptic, and the Methods do not seem to spell out how figure 3k was obtained. So, I am still struggling to understand: which proportion of enhancer variants with allelic imbalance of promoter contacts shows a significant RNA imbalance of the inferred target gene, at which FDR? This is key to the MCCv method and should be spelled out in the main text, to support the strong claims in Title and Abstract (see above).

If I understood it correctly, starting with 405 unique enhancer variants, 251 could be linked to a primary nominated gene (lines 207-209). But then the figure legend says that only 48 primary nominated genes for variants with evidence of MCCv changes were tested in figure 3k… Please clarify. Furthermore, figure 3k shows that only ~29% of these 48 show allele-specific expression, i.e., only 14 genes? And of the 25 secondary nominated genes ~24%, so 6 genes? Hence, starting with 405 enhancer variants, 20 genes could be linked to a target gene with an approximate FDR of ~25% (considering the random background of 7-9%)?? If I am mistaken with these calculations - then please spell out the correct calculations in the main text. If I am correct, then also please spell out in the main text (and abstract) that MCCv finds that roughly 1 in 20 enhancer variants can be assigned functionally to a target gene, at an FDR of ~25%. These numbers are key!

Let me emphasize that I would not be disappointed if only 1 out of 20 enhancer variants has a detectable effect. This may be real biology, and such estimates are enormously useful for the community. An FDR of 25% is borderline acceptable, and perhaps the authors could indicate how they expect this to be improved in future versions of MCCv.

We are very grateful to the reviewer for taking the time to review our data so carefully and for the clear arithmetic and guidance. We have tried to summarise the logic and numbers below:

1. We nominated 251 protein coding genes from 405 variants. These were derived from the level of MCC contacts only, without utilising the single-allele components of the data and we have clarified this in the abstract. This nomination is based on the frequency of contact with the gene promoter. The drop to 251 is a mixture of three things – firstly, we could not detect significant contacts between 14% of SNPs and a gene promoter (due to a mixture of poor oligo probe capture efficiency, inclusion of CTCF variants, which often do not make contacts with promoters (e.g. *SESN3* variant) and inclusion of some non-T cell enhancer variants as controls). Of the remaining 347 variants, 77 (22.2%) contacted the same gene

leaving 270 primary-nominated genes based on bulk 3C contact data and of these 251 are protein coding.

2. Figure 3 then details our exploration of single-allele analysis applied to the 241 (out of 405) variants, which were heterozygous in at least one donor.
3. For Figure 3i, we tested for allelic imbalance in paired RNA-seq data at all primary and secondary nominated genes linked to a heterozygous SNP in an enhancer. We tested 102 SNP gene pairs (from 88 SNPs) as certain variants had both primary and secondary genes pass phasing requirements. The drop from 241 heterozygous variants is due to our strict requirements for successful phasing of the enhancer variant with the gene-body haplotype (which required at least 25 gene-body SNPs separated by 250 bp). It is from this point that we can calculate the number of enhancer variants with a detectable effect on gene expression.
4. Figure 3j details our exploration of whether variants with changes in 3C contact between alleles make it more likely that they will be linked to allelically imbalanced gene expression beyond background signal. First we wanted to calculate a genome-wide background. For this, we calculated that within our donors an average of 10.0% of genes displayed allelic imbalance within the RNA-seq dataset. We also calculated a second background by estimating the percentage of genes which displayed allelic imbalance, which contacted variants with no evidence of allelic MCCv skew (8.9%, 17 out of 190 genes). This rate is similar if other criteria are used (for example, at loci where there is a detectable RNA skew and a change chromatin structure with the primary or secondary gene, the rate of allelic RNA imbalance for other, more weakly contacting genes is 9.5%).
5. We then calculated the percentage of variant-gene pairs with changes in allelic MCC contacts and calculated enrichment scores relative to the genome-wide background. 125 of 241 heterozygous variants displayed evidence of 3C skew. These were intersected with the 102 MCC linked genes at which we could potentially detect RNA skew (i.e. contained at least 25 gene body SNPs). 56 of these genes were contacting a SNP with a change in MCC contacts and 46 to unchanged SNPs. Overall, 35.5% (16 of 45, 1 in 2.81) of variants with a change in MCC contacts were associated with RNA skew at either the primary or secondary gene (30.3% of primary nominated and 26.1% of secondary nominated genes). Using the percentage of primary nominated genes with RNA skew linked to SNPs with 3C changes, this represents a 3.0 -fold enrichment over the genome-wide background.
6. We agree with the reviewer that it is informative to calculate whether MCCv contact changes enrich for genes with allelic imbalance relative to genome-wide skew. However, we think it is difficult to calculate the FDR for SNP-to-gene assignments from these data.

   A key issue is that an FDR calculation requires a well-defined null, i.e. a background rate that reflects truly non-functional events. In this case, the observed ~10% genome-wide rate of allelic skew is unlikely to represent purely technical noise and likely includes a substantial proportion of biologically

meaningful regulatory variation. As a result, this background rate cannot be directly interpreted as a false positive rate.
This distinction materially affects the inferred FDR. For example, if the full 10% background signal were assumed to be noise, the implied FDR would be ~33%. However, if only half of this background signal were truly null, the FDR would be ~16.6%, and if only 10% were null, the FDR would fall to ~3%. Thus, the FDR estimate is highly sensitive to assumptions about the proportion of background signal that is truly null, which is not directly observable from these data. For this reason, we feel that it would be safest to purely report the enrichment relative to the background.
In principle, it might be possible to estimate a null background from non-contacting genes in the vicinity of the variant when a candidate gene has been defined for the FDR of the SNP-to-gene assignment. However, we worry that this relies on additional assumptions (e.g. independence of regulatory effects across neighbouring genes and absence of shared regulatory influences) and may be confounded by local genomic context. To obtain an FDR of 0% in the context of a perfect assay, no skew could be detected in any of the genes surrounding the SNP which are not contacting the gene, which is unlikely given the pervasive nature of regulatory variation.
Nevertheless, if the reviewer feels that an approximate FDR estimate would be helpful, we would be happy to include this with appropriate caveats regarding the underlying assumptions.

We think that the key limiting factor in this type of analysis is the sensitivity and noise of the allelic RNA data, and we have recently pre-printed an improved intron-rich nascent RNA technique for this purpose (https://www.medrxiv.org/content/10.64898/2025.12.18.25342557v1).

We hope this breakdown of the logic and numbers is clear to the reviewer. Notably, some of the numbers will have slightly changed from the last submission due to a change in our phasing algorithm, which is discussed in Review point 4. We amended the abstract to avoid confusion, lengthened the results section related to Figures 3i and j to improve detail, clarified the figure legend, and finally added a new Methods section "Estimating background allelic expression for MCCv enrichment" detailing this analysis.

2a. Addition of the new statistical test is a major improvement. The "gallery" (now much expanded) of patterns is now available from Zenodo; it would be good to refer to this supplementary dataset in the main text.

We have now referenced the Zenodo repository in the Discussion of the main text.

2b. OK!

3. OK!

4. Even though the SNPs are covered by distinct RNA fragments, the fragments are still from the same gene and hence they are not independent (if the second half of the gene produces more RNA, then of course the first half does this too). Averaging multiple SNPs in a gene certainly adds robustness to the allele-specific RNA estimate (per replicate) but it cannot substitute for statistical analysis across multiple independent replicates. So, I still think the p-values are incorrect and overly optimistic... BTW: Figure panel 1i now only has labels, no data.

We thank the reviewer for this comment. The p-value reported for these genes reflects the statistical confidence in detecting allelic imbalance within the donor rather than across biological replicates (as would be the case for analyses such as eQTL studies). We therefore do not interpret these as causal effects, but rather as evidence of donor-specific allelic skew in the gene contacted by the variant. We have clarified this explicitly in the manuscript.

To address the concern regarding non-independence, we have further assessed the effective independence of signals across SNPs within genes and reanalysed the data to increase the robustness. The RNA-seq data were derived from ~5 million cells, so each read likely reflects an independent transcriptional event from a large population of cells. We analysed how the difference in the ratio of allelic imbalance between all phased heterozygous SNPs within individual genes changes with distance within and between donors. We initially used a separation of 153 bp but have revised this to 250 bp to ensure that SNPs are only used which are on different RNA molecules.

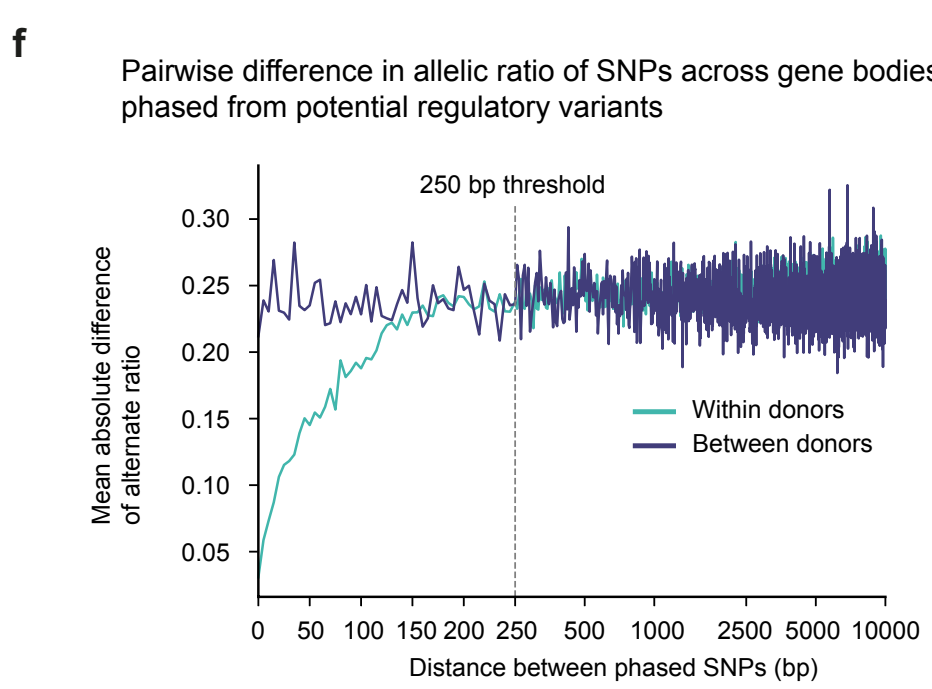

Extended Data Figure 5f. The absolute difference in alternate ratio (alternate counts/total counts) between pairs of SNPs with different distances within the same gene body. This was compared within single donors (green) and between donors (purple). The distance threshold cutoff used in the RNA skew analysis to define independent SNPs is shown as a dashed grey line. For each SNP-gene pair, all pairwise combinations of SNPs in the gene body were analysed within and between donors, and the absolute difference in the alternate ratio read fraction was computed for each pair. Pairs were binned by inter-SNP distance in 5 bp windows, points represent the mean absolute difference per bin.

We apologise for the error in Figure 1i, which has been corrected; the data were inadvertently missing in the previous revision due to a PDF rendering error.

5. I could not find the results mentioned in the response in the revised manuscript. Which figure?

Thank you for pointing this out – this is now included in the text of the manuscript.

6. I found part of this response in the Discussion; perhaps the details can be added to the Methods?

We have added these details to the Methods in the section "Oligonucleotide hybridisation, capture and sequencing".

New minor points:
- Color key missing in figures 1c,d

We have added colour bars to all heatmaps associated with manuscript figures.

- Figure 1i only has labels, data is missing

We apologise, this was an issue with PDF rendering and has now been fixed.

- Legend extended fig 1h erroneously says i instead of h

Fixed.

- line 125: text should state where the 42 maps can be found

We have added the Zenodo directory link to the Discussion as it contains maps for the Jurkat variants and the disease associated variants captured from T cells.

=======Reviewed by Bas van Steensel. Review assignment accepted: 4 Feb 2026; completed: 14 Feb 2026. It is my standard policy to sign *all* my manuscript reviews, regardless of my comments and recommendations. All correspondence should go via the editor. PLEASE DO NOT REMOVE THIS DISCLOSURE. =============

Reviewer #3 (Remarks on code availability):

I could not access the code without registering on an Oxford University site. While I disclose my identity as a reviewer I do not think it is appropriate to require this. Code should be made available through Github, which is the standard platform.

The core MCC code is subject to a commercial license held by the University of Oxford so we cannot deposit the single-allele analysis code to GitHub. We agree with the reviewer that it would be inappropriate for reviewers to disclose their identity, hence why we included the licensed code as zipped GitHub repos within the supplementary material on manuscript central for the purpose of review. The same repositories can be accessed from Oxford University Innovation, and this will be available freely to academics. The SNP prioritisation pipeline is not under this licensing agreement and the

Github repository link was provided (https://github.com/Davies-Genomics-and-Genome-Editing-Lab/snp_prioritization_pipeline).

Response to Reviewer Comments

Reviewer #3:
Remarks to the Author:
0 (not numbered in previous exchange): Whether it is appropriate to call MCCv a variant-to-function assay: Consider the parallel with protein biology. A protein has a certain folding, which we usually refer to as the structure of the protein, not its function. Likewise, enhancer structure and chromatin folding are structural features, not function. But I leave it up to the editor to decide whether structural features can be called "function", and hence whether it is appropriate to call MCCv a variant-to-*function* assay, even though only a minority of identified variants show effects on RNA expression when tested.

We have further reviewed the text in light of this comment.

Point 1. The explanation clarifies the calculations for this reviewer, but not really for the reader? It is a complex series of filters, admittedly too complex for the main text. But presently, the Results do not make it easy for the reader to understand this. The main text could perhaps highlight the key numbers in a simple short summary (e.g., we started with 405 variants; for various technical reasons ** dropped out; in the end we tested ** variants and found 45 variants a change in MCC contacts, of which 16 were confirmed by allele-specific RNA-seq). Note that I may mis-quote the numbers; I had to gather them from different parts of the Results text. A more detailed step-by-step explanation as in the rebuttal could then be provided as SuppInfo.
The argument that the FDR cannot be estimated weakens the case for the reliability of MCCv, to be honest. It seems best to adhere to the most conservative estimate, which is 33% based on the background measurements -- I do think that the reader should be made explicitly aware of this FDR estimate and how it is determined. Once again, I do not think that publication should depend on whether these numbers are high or low, but full clarity and transparency are important.

We thank the reviewer for this comment. We reviewed the results section to try to improve clarity. We felt it was important to give provide further details in light of the reviewer's comment and have therefore created a supplemental note **(Supplementary Note 1)** which details the series of filters and includes explanation of the methodologies, including explicit discussion of the FDR calculations.

2-6 and minor points: OK.
